# Supplementary material for: Factors leading to disparity in lung cancer diagnosis among black/African American communities in the USA: a qualitative study
Source: BMJ Open. 2023 Oct 29;13(10):e073886. doi: 10.1136/bmjopen-2023-073886 (PMC10619042; doi:10.1136/bmjopen-2023-073886)
Supplement: Supplementary data [file bmjopen-2023-073886supp001.pdf]

Appendix 1. Interview guides.

Semi-Structured Interview Guide

The study has two aims: Aim 1: Explore pitfalls, suboptimal experiences, and discriminatory practices in cancer diagnosis pathways for African Americans with metastatic lung cancer and oncogenic alterations. Aim 2: Identify etiologies of health disparity in cancer diagnosis for African Americans with metastatic lung cancer.

Cancer diagnosis

a. Tell me about your cancer diagnosis story, starting from when you noticed your first symptoms.

b. Patient phase
   i. What symptoms did you have that led to the cancer diagnosis?
   ii. How did the symptoms change over time before diagnosis?
   iii. Did anyone else notice your symptoms?
   iv. What did you think was the cause of your symptoms?
   v. How concerned were you about your symptoms and why?
   vi. What prompted you to seek medical care the first time?
   vii. Where did you seek care for the first time? Who else did you see?
   viii. How long do you think your symptoms were present before you sought care?
   ix. In your opinion, what contributed to that time (delay) between symptoms starting and seeking medical care?

c. PCP phase
   i. Describe your first interaction with your healthcare providers when you presented with symptoms.
   ii. What took place during the visit?
   iii. How did the provider explain your symptoms?
   iv. What were the steps taken to investigate your symptoms?
   v. What was the follow-up plan?
   vi. Were you offered any treatment for your symptoms? What was it?
   vii. Were you referred anywhere else? To whom?
   viii. How long after your doctor's visit did you get your first imaging test?
   ix. How was the decision made to get an imaging test?
   x. How were the findings on the imaging tests explained to you?
   xi. Was there any other tests ordered? What are they?
   xii. How much wait time did you have between your doctor's visit and getting the tests?
   xiii. In your opinion, what contributed to that time (delay) between seeking medical care and getting imaging tests?

d. Specialty care phase
   i. How was the decision made that you should see a specialist?
   ii. Describe the interactions with the specialists (pulmonologist, surgeon, radiologist, etc.) that led to getting a biopsy to confirm the diagnosis.
   iii. How were the results explained to you?
   iv. What other tests or procedures did you have early on?
   v. Share about your first interaction with the oncologist.
   vi. How much wait time did you have between receiving the cancer diagnosis and seeing an oncologist?
   vii. How was the *diagnosis* explained to you? What did they say about the disease you have?

viii. How was the *prognosis* explained to you? What did they say about what to expect will happen?
ix. What did they explain about the treatment options?
x. How was the decision made to get molecular testing?
xi. How were the tests explained to you?
xii. How were the results explained to you?
xiii. What treatment did you receive first?
xiv. How was the decision made to get a specific treatment?
xv. What other treatments have you received?
xvi. How much wait time did you have between your oncologist visit and starting treatment?

e. Before cancer
   i. How was your health before cancer?
   ii. Share about your lifestyle before cancer.
   iii. Have you smoked? If so, have you been offered smoking cessation?
   iv. How much did you smoke? If significant amount, have you been offered lung cancer screening?
   v. Have you had exposure to any carcinogen?
   vi. Share about encounters with healthcare before cancer.
   vii. What health issues did you deal with before cancer?
   viii. Did you have a PCP? Tell me about them? if not, why?

f. Reflections on etiologies of potential delays
   i. You mentioned, [example of possible inadequate/delay/suboptimal], from your vantage point, why do you think this happened?
   ii. What can be done to avoid such pitfalls from happening to other people who maybe in similar positions to yours?
   iii. (rephrase questions of focus group to ask patients/caregivers to identify factors and structures contributed to less than optimal outcomes for them or other people in their situation?)

Demographics

- What is your age?
  - Under 18
  - 18-24
  - 25-44
  - 45-64
  - Over 65
- What is your gender?
  - Male
  - Female
  - Other (please specify)
  - Prefer not to say
- What is your race/ethnicity?
  - White
  - Hispanic/Latino
  - Black/African American
  - Native American/American Indian
  - Asian/Pacific Islander
  - Other

- What is the highest level of school you’ve completed?
    - Less than a high school diploma
    - High school diploma or equivalent
    - Some college
    - Bachelor’s degree
    - Master’s degree
    - Doctorate
    - Other (please specify)
- What is your current employment status?
    - Employed Full-time (40 hours per week)
    - Employed Part-time (Less than 40 hours per week)
    - Unemployed
    - Student
    - Retired
    - Self-employed
    - Unable to work
- What is your home zip code?
- What is your marital status?
    - Single (Never married)
    - Married
    - Divorced
    - Widowed
- What is your household income?
    - Below $10K
    - $10K – $50K
    - $50K – $100K
    - $100K - $150K
    - Over $150K
- Cancer date of diagnosis:____
- Type of cancer:____
- Stage at time of diagnosis:____
- Stage now if not IV at time of diagnosis:____

Health Care Providers and Community Advocates

- We define cancer health disparity as the health difference, on the basis of one or more health outcomes, that adversely affects black/african americans. Specifically, We are interested in Identifying etiologies of health disparity around cancer diagnosis for African Americans with advanced lung cancer and oncogenic alterations.
- We want you help understanding the factors that cause this disparity. We are using the NIMHD model for health disparity. According to this framework, we examining the issue across 5 domains and 4 levels. See figure.

Domains of Influence (Over the Lifecourse)	Levels of Influence*: Individual	Interpersonal	Community	Societal
Biological	Biological Vulnerability and Mechanisms	Caregiver–Child Interaction<br>Family Microbiome	Community Illness Exposure<br>Herd Immunity	Sanitation<br>Immunization<br>Pathogen Exposure
Behavioral	Health Behaviors<br>Coping Strategies	Family Functioning<br>School/Work Functioning	Community Functioning	Policies and Laws
Physical/Built Environment	Personal Environment	Household Environment<br>School/Work Environment	Community Environment<br>Community Resources	Societal Structure
Sociocultural Environment	Sociodemographics<br>Limited English<br>Cultural Identity<br>Response to Discrimination	Social Networks<br>Family/Peer Norms<br>Interpersonal Discrimination	Community Norms<br>Local Structural Discrimination	Social Norms<br>Societal Structural Discrimination
Health Care System	Insurance Coverage<br>Health Literacy<br>Treatment Preferences	Patient–Clinician Relationship<br>Medical Decision-Making	Availability of Services<br>Safety Net Services	Quality of Care<br>Health Care Policies
Health Outcomes	Individual Health	Family/ Organizational Health	Community Health	Population Health

This is the cancer diagnosis pathway.

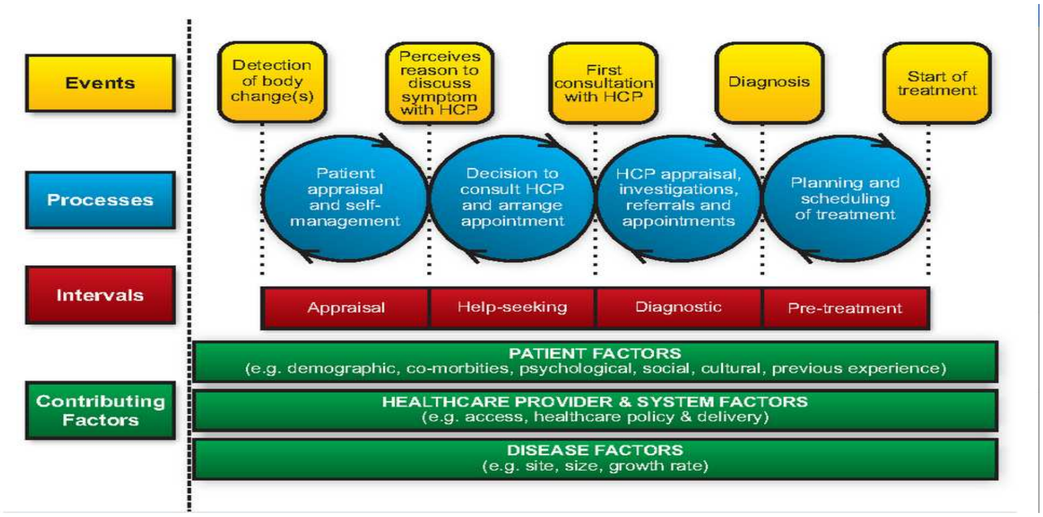

1. What are factors and structures that lead to delays in detecting first symptoms?

2. What are factors and structures that lead to inappropriate appraisal of symptoms?
3. What are factors and structures that lead to spending longer time in self-management phase as opposed to seeking help?
4. What are factors and structures that lead to not perceiving reasons to discuss symptoms with health care providers in a timely way?
5. What are factors and structures that lead to delays in consulting health care providers?
6. What are factors and structures that lead to not being able to arrange for appointments?
7. What are factors that lead to inadequate appraisal of symptoms by the health care provider on early visits?
8. What are factors and structures that lead to not ordering appropriate investigations in a timely manner?
9. What are factors and structures that lead to not arranging for referrals once suspicion of cancer become high (i.e., delays in getting to be seen by a specialist)?
10. What are factors and structures that lead to failing to obtain molecular testing
11. What are factors and structures that lead to not getting molecular testing?
12. What are factors and structures that lead to delays in getting molecular testing?
13. What are factors and structures that lead to delays in starting appropriate targeted therapy?

Caregiver's interviews are similar but rephrased to get the perspectives of the significant other on the health/experience of the patient.

Demographics

- What is your age?
  - Under 18
  - 18-24
  - 25-44
  - 45-64
  - Over 65
- What is your gender?
  - Male
  - Female
  - Other (please specify)
  - Prefer not to say
- What is your race/ethnicity?
  - White
  - Hispanic/Latino
  - Black/African American
  - Native American/American Indian
  - Asian/Pacific Islander
  - Other
- What is the highest level of school you've completed?
  - Less than a high school diploma
  - High school diploma or equivalent
  - Some college

    - Bachelor's degree
    - Master's degree
    - Doctorate
    - Other (please specify)
- What is your current employment status?
    - Employed Full-time (40 hours per week)
    - Employed Part-time (Less than 40 hours per week)
    - Unemployed
    - Student
    - Retired
    - Self-employed
    - Unable to work
- What is your home zip code?
- What is your marital status?
    - Single (Never married)
    - Married
    - Divorced
    - Widowed
- What is your household income?
    - Below $10K
    - $10K – $50K
    - $50K – $100K
    - $100K - $150K
    - Over $150K
