# Supplementary material for: Factors leading to disparity in lung cancer diagnosis among black/African American communities in the USA: a qualitative study
Source: BMJ Open. 2023 Oct 29;13(10):e073886. doi: 10.1136/bmjopen-2023-073886 (PMC10619042; doi:10.1136/bmjopen-2023-073886)
Supplement: Author's manuscript [file bmjopen-2023-073886.draft_revisions.pdf]

BMJ Open

BMJ Open is committed to open peer review. As part of this commitment we make the peer review history of every article we publish publicly available.

When an article is published we post the peer reviewers' comments and the authors' responses online. We also post the versions of the paper that were used during peer review. These are the versions that the peer review comments apply to.

The versions of the paper that follow are the versions that were submitted during the peer review process. They are not the versions of record or the final published versions. They should not be cited or distributed as the published version of this manuscript.

BMJ Open is an open access journal and the full, final, typeset and author-corrected version of record of the manuscript is available on our site with no access controls, subscription charges or pay-per-view fees (http://bmjopen.bmj.com).

If you have any questions on BMJ Open's open peer review process please email info.bmjopen@bmj.com

BMJ Open

Factors Leading To Disparity In Lung Cancer Diagnosis Among Black/ African American Communities in the United States: A Qualitative Study

Journal:	*BMJ Open*
Manuscript ID	bmjopen-2023-073886
Article Type:	Original research
Date Submitted by the Author:	21-Mar-2023
Complete List of Authors:	Thuo , Nicholas; University of Washington Seattle Campus<br>Martins, Tanimola; University of Exeter, Primary Care; University of Exeter , Primary Care<br>Manley Jr. , Eugene ; LUNGevity Foundation<br>Standifer, Maisha ; Morehouse School of Medicine<br>Sultan , Dawood ; Mercer University<br>Hill , Angela ; University of South Florida<br>Faris , Nick ; Baptist Memorial Health Care Corp<br>Thompson, Matthew; University of Washington, Department of Family Medicine<br>Jeremiah, Rohan ; University of Illinois at Chicago,<br>Al Achkar, Morhaf; University of Washington, Family Medicine ;
Keywords:	Public health < INFECTIOUS DISEASES, Adult oncology < ONCOLOGY, Respiratory tract tumours < ONCOLOGY

SCHOLARONE™
Manuscripts

I, the Submitting Author has the right to grant and does grant on behalf of all authors of the Work (as defined in the below author licence), an exclusive licence and/or a non-exclusive licence for contributions from authors who are: i) UK Crown employees; ii) where BMJ has agreed a CC-BY licence shall apply, and/or iii) in accordance with the terms applicable for US Federal Government officers or employees acting as part of their official duties; on a worldwide, perpetual, irrevocable, royalty-free basis to BMJ Publishing Group Ltd ("BMJ") its licensees and where the relevant Journal is co-owned by BMJ to the co-owners of the Journal, to publish the Work in this journal and any other BMJ products and to exploit all rights, as set out in our licence.

The Submitting Author accepts and understands that any supply made under these terms is made by BMJ to the Submitting Author unless you are acting as an employee on behalf of your employer or a postgraduate student of an affiliated institution which is paying any applicable article publishing charge ("APC") for Open Access articles. Where the Submitting Author wishes to make the Work available on an Open Access basis (and intends to pay the relevant APC), the terms of reuse of such Open Access shall be governed by a Creative Commons licence – details of these licences and which Creative Commons licence will apply to this Work are set out in our licence referred to above.

Other than as permitted in any relevant BMJ Author's Self Archiving Policies, I confirm this Work has not been accepted for publication elsewhere, is not being considered for publication elsewhere and does not duplicate material already published. I confirm all authors consent to publication of this Work and authorise the granting of this licence.

For peer review only

Factors Leading To Disparity In Lung Cancer Diagnosis Among Black/ African American Communities in the United States: A Qualitative Study

Nicholas Thuo[1], Tanimola Martins[2], Eugene Manley, Jr.[3], Maisha Standifer[4], Dawood H. Sulta[5], Nick Faris[6], Angel Hill[7], Matthew Thompson [1], Rohan D. Jeremiah[8], Morhaf Al Achkar[1]

1. Department of Family Medicine, University of Washington, Seattle, WA;

2. University of Exeter Medical School

3. LUNGevity Foundation, Chicago, IL;

4. Morehouse School of Medicine, Satcher Health Leadership Institute, Atlanta, GA

5. Mercer University, Macon, Georgia

6. Baptist Memorial Health System, Memphis, TN;

7. University of South Florida, Tampa, FL;

8. University of Illinois Chicago, College of Nursing, Chicago, IL;

Corresponding author

Morhaf Al Achkar

Department of Medicine, University of Washington, Seattle, WA;

Tel: +2068009480

Email: alachkar@uw.edu

Keywords: Lung cancer, disparity, sub-optimal experiences, NIMHD model for health disparity

Abstract word count: 244

For peer review only - http://bmjopen.bmj.com/site/about/guidelines.xhtml

Word Count: 3407

Tables: 3

Figures: 1

References: 37

For peer review only

For peer review only - http://bmjopen.bmj.com/site/about/guidelines.xhtml

ABSTRACT

Objective: To explore experiences related to lung cancer diagnosis among African American patients, caregivers, advocates, and health providers and identify contributing factors.

Methods: We conducted in-depth interviews with individuals knowledgeable about African Americans diagnosed with lung cancer. Participants included patients, caregivers, advocates, and healthcare providers. Thematic analysis identified suboptimal experiences at patient, primary care, and specialist levels, along with contributing factors according to the National Institute of Minority Health and Health Disparities (NIMHD) health disparity model.

Results: From March to November 2021, we conducted individual interviews with 19 participants, including 9 patients/caregivers and 10 providers/advocates. The median age group of participants was 45-64 years. Participants reported recurring and increased pain before seeking treatment, treatment for non-cancer illnesses, delays in diagnostic tests and referrals, poor communication and bias when dealing with specialists and primary care providers. Factors contributing to suboptimal experiences included reluctance by insurers to cover costs, provider unwillingness to conduct comprehensive testing, provider bias in recommending treatment, high healthcare costs, and lack of healthcare facilities and qualified staff to provide necessary support. Some participants reported positive experiences due to their insurance, availability of services, and having an empowered support structure.

Conclusions: Suboptimal experiences were common among African American patients and caregivers, and factors contributing to these experiences are consistent with the NIMHD model for health disparity. However, additional research is needed to fully capture the extent of these experiences and identify ways to improve African American experiences in the lung cancer diagnosis pathway.

For peer review only

For peer review only - http://bmjopen.bmj.com/site/about/guidelines.xhtml

KEY MESSAGES

What is already known on this topic:

Black/African Americans have the highest morbidity and mortality rates for most cancers compared to any other racial group. Addressing the root causes of sub-optimal experiences for African Americans in cancer diagnosis pathways can help mitigate such disparities.

What this study adds:

Our study delves into the experiences of lung cancer patients, caregivers, and healthcare providers along the diagnostic and treatment pathways, highlighting the factors contributing to sub-optimal experiences.

How this study might affect research, practice, or policy:

A deeper understanding of these experiences and the factors influencing them could lead to positive changes in provider-patient interactions, identify areas for improvement in provider training and health systems, and improve cancer diagnosis and treatment pathways for African Americans.

For peer review only

INTRODUCTION

Lung cancer disproportionately affects Black/African American (BAA) communities.(1-5) Compared to other races, BAA men have a higher lung cancer death rate of 55.4 per 100,000, versus 46.9 in men of other races.(6) In 2022, BAA had a lower estimated 5-year survival rate (20%) compared to Whites (22%).(5) BAA have a lower rate of early-stage detection for lung cancer, at only 16% (compared to 20% among all races), with 53% being diagnosed at distant locations (compared to 49% for non-Hispanic whites).(5, 6) BAAs are less likely to be staged during diagnosis, and to receive curative treatment at the most optimal point in the course of the disease.(2, 7-9) These disparities appear to be unrelated to health insurance, as BAA Medicare beneficiaries with lung cancer experience higher mortality rates than non-Hispanic Whites.(10)

The NIMHD research framework offers guidance on considering the domains and levels of influence of factors that may account for why lung cancer disproportionately affects BAAs.(11) Differences in nicotine metabolism, which are linked to genetics and heredity, have been identified as a potential biological vulnerability.(1, 12, 13). Documented behavioral factors include smoking patterns, exposure to secondhand smoke, and care avoidance due to beliefs and fears.(1, 14) Environmental factors, including greater exposure to radon and indoor pollutants, living near smelting factories, and working in coal mines, have been identified as potential contributing factors to these disparities.(15) Lower educational attainment, rurality, poverty, and limited access to healthcare due to employment are significant sociocultural factors.(16) Lower educational attainment, rurality, poverty, and limited access to healthcare due to employment are significant implicated sociocultural factors.(17, 18) Research further indicates that systemic racism leading to inequitable access to quality healthcare is the critical factor for the gap in outcomes when a BAA develops lung cancer.(19, 20)

Routine use of molecular testing and targeted cancer therapy for late-stage non-small cell lung cancer (NSCLC) has significantly reduced mortality rates.(21, 22) Patients who receive molecular testing within 60 days of diagnosis have better survival rates compared to those who don't.(23) Studies have found that BAAs have similar rates of oncogenic alterations as non-Hispanic Whites and at least one in every three BAA patients have an actionable mutation.(24) Nevertheless, BAAs to receive molecular testing or targeted treatment less frequently than Whites.(23) Compared to non-Hispanic Whites, BAA patients over 65 years old with NSCLC had lower odds ratios of 0.53-0.63 for receiving targeted therapy. (25, 26) White patients with NSCLC were more likely to receive next-generation sequencing, compared to BAA patients (50.1% vs 39.8%, p<0.0001).(27) Limited access to testing among BAA patients may be contributing to disparities in outcomes.(28) Studies have shown that when given access to care, BAA lung cancer patients have the same outcomes as White patients.(24, 29)

Extensive literature exists on the roots of disparities and how to eliminate them for patients with early-stage lung cancers. However, little is known about the etiologies of disparity for patients with late-stage lung cancer. While it is possible that etiologies of disparities in treating metastatic diseases share some commonalities with early-stage cancers, they may also have unique attributes contributing to the complex and long-term treatment paradigm of metastatic diseases.

For peer review only - http://bmjopen.bmj.com/site/about/guidelines.xhtml

This study aimed to achieve two objectives: the first was to explore the pitfalls, suboptimal experiences, and discriminatory practices in diagnostic pathways for BAAs with lung cancer. The second objective was to identify the etiologies of health disparity in cancer diagnosis for BAAs with lung cancer.

METHODS

Study Setting, Population, and Design

We utilized a phenomenology research design to guide qualitative interviews conducted with patients, caregivers, healthcare professionals, and community advocates.(30) Patient and caregiver interviews provided patient-level accounts to identify diagnostic pathways and challenges encountered. We identified suboptimal experiences during the diagnostic phase. Healthcare professional and community advocate interviews produced reflections and observations to contextualize possible causes of observed challenges for individuals in BAA communities. The study was co-designed and conducted with the help of a community advisory group (CAG) called Project RADICAL - Racial DIsparities in CAncer of the Lung - formed in 2020. The CAG, consisting of patients, caregivers, lung cancer researchers, and community advocates, met biweekly to develop the study protocol, inform study methods and interview guides, and interpret findings. The University of Washington's Institutional Review Board approved the study.

Patient and Public Involvement

We used a purposive sampling strategy to identify participants who met the inclusion criteria, which included patients aged 18 years or older who self-identified as BAA and had been diagnosed with metastatic lung cancer in the United States. Caregivers were individuals providing primary care for a BAA lung cancer patient. Healthcare providers (HCPs) and community advocates were practicing physicians providing oncology or primary care services, or advocates for BAA communities. To identify participants, we used snowball sampling methods through the community network of Project RADICAL CAG members, collaborators' networks in cancer organizations, online support groups such as ALK-positive, and academic list servers focused on disparities.

Data Collection

The research team developed the interview guides using the Anderson's model of pathways to cancer treatment.(31) Based on the findings of our previous work(31), the model was refined to organize patients' diagnostic experiences into three phases: 1) patient phase (from first symptom recognition to the first doctor's visit in primary care), 2) primary care phase (from the first primary care visit to presumptive diagnosis based on imaging), and 3) secondary care phase (from referrals to biopsy until identifying actionable genes and starting treatment).(32) The patient and caregiver interviews began with open-ended questions about their experiences with a cancer diagnosis. HCP and community advocate interviews aimed to identify factors contributing to suboptimal experiences along the diagnosis pathway (see Appendix 1 for interview guides). An experienced qualitative researcher (MA) conducted all interviews via phone or video

For peer review only - http://bmjopen.bmj.com/site/about/guidelines.xhtml

conference, with most conducted via Zoom. Interviews were audio recorded and transcribed verbatim. Participants received a $50 gift card for participating in the 30-60 minute interviews.

Analysis

An experienced qualitative researcher (NT) coded the interviews using Dedoose (Dedoose Inc., Manhattan Beach, California) for analysis. The senior author (MA) provided peer debriefing and supervised NT's coding. Transcripts were analysed using inductive and deductive strategies, with codes developed highlighting suboptimal experiences and factors leading to disparity. The NIMHD research framework was used to organize the emerging factors contributing to disparity, and patient and caregiver narratives were triangulated with community advocates and HCPs' reflections. Findings were presented to the CAG and co-authors, and inputs were used to iteratively develop themes.

Researchers' Characteristics and Reflexivity

The research team brings diverse backgrounds, experiences, and perspectives to this qualitative study. MA is a male, PhD-trained qualitative researcher, identifying as an Arab immigrant, with personal experience living with advanced lung cancer. MS is a female, PhD-trained qualitative researcher, identifying as BAA, and has served as a caregiver for a BAA patient who succumbed to advanced lung cancer. NT, an MPH-trained and experienced qualitative researcher, identifies as Black African from Kenya. TM is a male, PhD-trained qualitative researcher, identifying as Black African from Nigeria. RDJ is a male, PhD-trained researcher with expertise in qualitative research, identifying as BAA. DHS is a male, PhD-trained health service researcher, identifying as BAA. NF holds a Master of Divinity and serves as a research manager and health equity leader; he identifies as a white male. AH is a female, PhD-trained pharmacist with experience in disparity research, identifying as BAA, and has had a family member who succumbed to advanced lung cancer. MT is a male, PhD-trained family doctor and researcher, who has also experienced the loss of a family member to lung cancer.

RESULTS

The demographic characteristics of the participants are presented in Table 1. Participants were aged 25–64 years old, with 55% female,78% black, and 58% were patients or caregivers. At the time of the interview, 94% were in full-time employment We will describe the themes that emerged in two main areas, namely suboptimal experiences and factors leading to disparities.

For peer review only - http://bmjopen.bmj.com/site/about/guidelines.xhtml

Table 1. Participants' demographics.

ID	Age Group	Gender	Race/Ethnicity	Education	Employment	Zipcode	Marital status	income (000 $)	Role
101	25-44	Female	black	Doctorate	Fulltime	11203	Single	<50	patient
102	45-64	Female	Black	some college	Fulltime	33705	Married	50-100	Caregiver
103	45-64	male	Black	Doctorate	Fulltime	46268	Married	100-150	Researcher
104	>65	Female	Black	Doctorate	Retired	60621	Divorced	50-100	patient
105	>65	female	Black	Masters	Fulltime	60652	Married	50-100	Caregiver
106	25-44	Female	Black	Bachelors	Fulltime	10701	Single	100-150	Patient
107	45-64	Female	Black	Bachelors	Fulltime	46234	Married	100-150	Patient
108	25-44	Female	Black	Bachelors	Fulltime	46268	Married	50-100	Advocate
109	45-64	male	Black	some college	Fulltime	91367	Single	50-100	Patient
110	45-64	male	Black	Masters	Fulltime	46278	Married	100-150	caregiver
111	45-64	female	Black	Doctorate	Fulltime	46214	Married	>150	caregiver
112	Unknown	male	Black	Doctorate	Fulltime	Unkown	Married	>150	oncologist
113	25-44	Female	Black	Doctorate	Fulltime	31008	Married	>150	Provider
114	45-64	Female	Black	Doctorate	Fulltime	15146	Married	>150	Provider
115	45-64	male	white	Doctorate	Fulltime	43452	Married	>150	Provider
116	45-64	male	white	Doctorate	retired	80111	Married	>150	provider
117	45-64	male	white	Doctorate	Fulltime	98115	Married	>150	provider
118	45-64	male	white	Doctorate	Fulltime	41042	Married	>151	Nurse Navigator
119	45-64	Female	Black	Doctorate	Fulltime	33579	Married	>150	caregiver

For peer review only

Suboptimal experiences along the diagnosis pathways

We organized the suboptimal experiences within the patient phase, the PCP/hospital/ER/urgent care phase, and the specialist phase. Figure 1 depicts suboptimal experiences throughout the diagnosis pathway. Tables 2 includes supportive quotes.

Patient Phase

- **Not being alarmed by the symptoms as they appeared.** Some patients did not seek care right away as they were not concerned about the symptoms either because of their mild nature or the failure to appraise their seriousness.
- **Explaining symptoms as caused by other conditions.** Patients and caregivers at times attributed symptoms to less serious health conditions, such as common cold and allergies, delaying seeking medication consultation.
- **Prolonging self-managing of symptoms.** Patients resorted to home remedies or over-the-counter treatment for cancer-related symptoms (e.g., cough and pain) instead of seeking medical care.
- **Seeking care only as symptoms became severe.** The threshold to seek medical advice was not crossed until symptoms were too serious to ignore or especially bothersome (e.g., severe pain).

PCP/Hospital/ER/Urgent Care Phase

- **Being treated for common, more benign conditions.** Patients frequently received treatment for more common and less serious conditions, such as sinus infections or acid reflux. Patients then ignored the symptoms without clear instructions or a follow-up plan.
- **Delayed or not being offered diagnostic imaging.** Some patients were not offered diagnostic tests due to the HCP's low levels of suspicion for lung cancer.
- **Not being informed of abnormal findings on tests.** Some patients were not informed about abnormal findings on their diagnostic tests and were left without any follow-up. On occasions, patients were not informed about the findings or the plan and they discovered them by reading the handouts.
- **Communicated information poorly and left with uncertainty.** Patients were left in the dark regarding the plans of care. Sometimes they were not told how they could receive help if they needed urgent care or had questions.

Secondary Care Phase

- **Difficulty finding an appointment to see the specialist.** Some patients, especially in underserved areas, had difficulty finding timely appointments to see specialists.
- **Poor delivery of information by the HCP.** Some patients were hassled during their care, receiving sub-par communication or little guidance to make decisions.
- **Not receiving molecular testing.** At times, patients were not offered molecular testing—a standard of care—and as a result, missed an opportunity to receive novel treatments.

For peer review only - http://bmjopen.bmj.com/site/about/guidelines.xhtml

- **Feeling steered to receive unnecessary chemotherapy.** Some patients felt they were pushed to take the only option of chemotherapy, while other more-novel treatment options were not discussed.

For peer review only

- Table 2. Supportive Quotes for The Suboptimal Experiences Along The Diagnosis Pathways

Patient phase	PCP/hospital/ER/urgent care phase	Specialist phase
**Not being alarmed by the symptoms as they appeared.**	**Being treated for common, more benign conditions.**	**Difficulty finding appointment to see the specialist.**
time went on and cough never got better, but it did not seem to get worse. [patient (107)]	They'll say, "Hey, it's sinus or allergies," they'll give them an antibiotic. [Nurse navigator (118)]	If you live in a rural community and you're the only doctor in that community and the nearest specialist is 2 hours or 45 minutes away that can have an effect on your referral process and your ability to refer at times. [Provider (113)]
	My primary care doctor diagnosed me with having allergies as well as acid reflux. I was treated with Protonix. I was treated with Claritin, over-the-counter stuff and eventually I would just ignore it. [patient (107)]	
**Explaining symptoms as caused by other conditions.**	**Delayed or not being offered diagnostic imaging.**	**Poor delivery of information by the provider.**
they had similar symptoms before and they resolved on their own without seeking help or with conservative measures; may be that if they have ignored in the past that can lead to worst consequence for them. [Provider (106)]	I went to another doctor and the first thing the doctor asked me “had you had an x-ray?” I was like, “No. Nobody sent me for an x-ray.” [Patient (106)]	It was just another bad experience where I was sitting in the doctors for my appointment. He was perpetually on the telephone talking to other people as I was sitting there and then kind of squeezing in like when he was on hold or between calls telling me something really quick. [Patient (109)]
she had a cough for quite a while and we thought that it was allergies. Then, unfortunately, she was in a head-on collision with a drunk driver and it broke the transverse process of her C7 vertebrae. [Caregiver (104)]	You can't do chest CTs in every person, that's not a reasonable approach. But I think that's, in part, where some diagnostic delays happen is that perhaps they didn't have a high enough pretest probability to do the subsequent downstream test. [Provider (117)]	I would think knowing that I am also in a medical field that you would have at least tried to explain to me. I did not think that I should have had to be like, I'm not leaving until somebody explains what happens. I do not think that I should have had to do that. [Patient (101)]
**Prolonging self-managing of symptoms.**	**Not being informed of abnormal findings on tests.**	**Not receiving molecular testing.**
he could not go to sleep at night so he would be taking NyQuil and it was more of a consistent thing. [caregiver (102)]	When I looked at that hospital discharge summary and saw that there were lung nodules there. I had not seen that before or ever been told or followed up about that. [caregiver (102)]	I had one or two patients diagnosed with lung cancer who went to a smaller local facility for their follow-up, and I brought them back into the office to go through things. And I said, "Well, I don't see any molecular testing on your biopsy."

For peer review only - http://bmjopen.bmj.com/site/about/guidelines.xhtml

		You've lost that opportunity [Thoracic oncologist (115)]
**Seeking care only as symptoms became severe.**	**Communicated information poorly and left with uncertainty.**	**Feeling steered to receive unnecessary chemotherapy.**
one night I woke up because I felt like my left side of the chest was closing down on me, I felt that my left side was not getting enough air in. [Patient (101)]	Nobody talked to her about the mass, but she was reading her orders and she was like, "What is this in my orders?" [Caregiver (111)]	They were very pushy with the chemotherapy which made me even more skeptical. I was just saying, "Wait a minute. I would like a second opinion." I had texted the doctor and said, "they want to do chemo immediately." She said, "No, let us wait. I will get him in next week and we can do a genetic test that can see if chemo is the best for him or targeted medicine." That was the first time I had heard about targeted medicine. [Caregiver (102)]
	So who do I call?Which doctor's office do I call? If I'm having a problem, do I go to the ER? Do I go to Urgent Care? Do I go to the pulmonologist because my cousin goes to one? Or do I go to my primary care doctor? [Provider (113)]	

For peer review only

Factors Leading To Disparity In Lung Cancer Diagnosis

Herein, we describe the factors that lead to suboptimal experiences along the pathways to diagnosis and treatment for BAA patients. These factors work at multiple individual, interpersonal, community, and societal levels. Tables 3 includes supportive quotes.

Individual Factors

- **Not seeking timely care.** Some patients reported an aversion to seeking timely medical care for fear of finding serious illnesses or having to deal with adverse outcomes.
- **Inadequate reporting of relevant symptoms**. The patients' experiences with disease symptoms influence their decisions on which symptoms to report. They may not feel comfortable reporting certain symptoms and may understate the details.
- **Logistic constraints to accessing healthcare.** Patients living in poorer areas tend to have poorer healthcare outcomes due to affordability and income issues. At times, patients live far from the health facility and are unable to access transportation or assistance to travel for treatment.
- **Avoiding medical care due to a previous encounter involving racial discrimination**. Perceptions of the HCP based on previous experiences often lead to mistrust and hesitancy among patients to avoid possible dismissals or maltreatment.
- **Cultural identity**. People are often influenced by their community's behavior and religious beliefs regarding healthcare.
- **Lack of health literacy**. At times, patients are unable to appreciate the severity of a diagnosis or are unaware of what to do when they experience health changes.
- **Not knowing which HCP to contact**. Information on HCPs and their specialties are not readily available, making it hard for the patient to determine who to reach out to and where.

Interpersonal Factors

- **Lack of compassionate family support.** For some individuals, weaker family ties lead to socioeconomic isolation, which obscures the management of their healthcare needs.
- **Doctors' schedules not accommodating patients.** In certain areas, the doctors' offices or health facilities are only open during weekdays, when many people are at work and are therefore unable to access healthcare.
- **Societal pressure to seek non-medical approaches.** It is often observed that the patient's social networks encourage the use of alternative treatment methods, including herbal and non-regulated remedies. As a result, some patients may choose spiritual practices or herbs over medical care.
- **Patients' surroundings normalize tolerating symptoms.** Patients seldom internalize the need to endure the disease and fear the loss of dignity through their dependence on others, especially where the symptoms are common around them.
- **HCPs indulging in discriminatory practices**. It has been observed that HCPs tend to neglect, miss, or dismiss symptoms reported by BAA patients, especially pain. Furthermore, they may not execute appropriate referrals.

For peer review only - http://bmjopen.bmj.com/site/about/guidelines.xhtml

- **Patients anticipating prejudice and discrimination**. Patients are concerned about how HCPs may treat them based on their personal attributes, especially race. Others, especially ex-smokers, avoid seeking medical care due to the fear of being blamed for their illness and the associated stigma of causing cancer.

Community Factors

- **Cultural practices concerning healthcare.** Certain therapies may be preferred in a community, for example, obtaining painkillers instead of identifying the cause of the ailment.
- **Availability of clinical services.** There is a dearth of hospitals, diagnostic facilities, primary care providers (PCPs), and specialists within certain communities and areas. This is especially true for smaller communities living in poor areas.
- **Disjointed clinical services**. Referrals and visits to different departments and hospitals may force patients to drop out of treatment and follow up on results. Furthermore, communicating the results to PCPs after visiting the emergency room is left to the patient and not communicated directly, which often leads to missed opportunities for follow-up on diagnosis and treatment.
- **Delays in obtaining appointments**. Although booking systems for hospitals are now online, navigating these systems is complex and coupled with organizational constraints in the availability of appropriate staff. A patient's diagnosis may be delayed due to a lack of appointment slots.
- **Organizational demands on HCPs to see more patients.** For profitable practice, some hospitals and clinics set patient and charge goals for HCPs, which leads to poor service quality. Also, forcing HCPs to attend to more patients within a stipulated time may overwhelm them, reducing their quality of care.

Societal Factors

- **Working in lower-paying jobs.** The relegation of certain communities to low-paying jobs limits their access to resources for managing health and disease impacts. This economic disempowerment leaves personal healthcare needs competing for priority with one's family's needs. Often, family commitments deny the patient the appropriate time to seek treatment.
- **Cost of receiving care**. Seeking diagnosis and treatment is costly for individuals, especially when they face job insecurity and can take limited time off from work. This issue, coupled with the inflated costs of medical care, makes healthcare services inaccessible or undesirable.
- **Limited or variable insurance coverage**. Limited options within Medicaid coverage, cost of insurance, and the inability to obtain insurance led to suboptimal access to timely diagnosis and treatment. Besides, the unwillingness of insurance companies to pay for certain services, either in full or part, may limit one's options for quality care.
- **Policies and laws determining investment in healthcare**. The existing policies and laws related to healthcare and resources available to communities affect access, education, and living conditions for the community.

For peer review only - http://bmjopen.bmj.com/site/about/guidelines.xhtml

- **Limited management using telehealth**. Only using telehealth and the absence of physical testing and comprehensive workups lead to missed diagnoses.
- **Practitioner's inexperience**. Underprivileged communities are often subjected to care by undertrained practitioners and a shortage of specialists. A lack of adequate skill and experience to manage chronic diseases may lead to lower suspicion or missing critical symptoms.
- **Complex health systems not designed for vulnerable patients**. Moving through complex online hospital systems and departments to book and access essential services requires internet access either by computer or phone, which can be challenging for many.

For peer review only

Table 3. Supportive Quotes for The Factors Leading to Disparity in Lung Cancer Diagnosis

Individual Factors	Interpersonal Factors	Community Factors	Societal Factors
**Not seeking timely care.**	**Lack of compassionate family support.**	**Cultural practices concerning health care.**	**Working in lower-paying jobs.**
Yeah. I mean, I think it's related to, "I hope that things will get better!" It can also be related to a form of denial So, in fact, they know it might be lung cancer and are not going in as a form of denial. [Provider (117)]	If you were in the majority in a community, you're more likely to have a wider network within that community. If you have a cough, you're more likely to be talking to somebody about it and somebody said, "So-and-so had a cough. This was what it was. You should get it checked out." If you are more isolated within the community because of race or economic issues [you won't have] that network of people that will provide advice to you outside of the medical setting [provider (115)]	some people take their symptoms more seriously than others, right? That's a personality-based thing amongst individuals, and I don't know whether this is more or less among African Americans, but to the extent to which symptoms are minimized or ignored, that's on that kind of more personal or personal cultural, right? [provider (117)]	You have a lot of, between African-American and Hispanic Community, a lot of them do not have insurance, rather because they cannot afford healthcare insurance. Because they are making minimum wage jobs or it just not on the priority list of "I have never been sick [Advocate (108)]
**Inadequate reporting of relevant symptoms.**	**Doctors' schedules not accommodating patients.**	**Availability of clinical services.**	**Cost of receiving care.**
What's important to the patient and what sometimes important to the doctor are two different things. So, they may not consider their shortness of breath and issue because they've been having it over a period of time and it's manageable as long as things are manageable, people will ignore them.' [Provider (113)]	A lot of jobs, you know, the timing, like the doctor's offices, closes at five o'clock, and they normally take their last patient at 4.30. [advocate(108)]	I think it's also where is the setting of the low-dose CT scans. Most of them are in hospitals, or some patients just won't go to a hospital for care. And so, the place of the low-dose CT scans could potentially be a barrier [Provider (114)]	They're thinking, "Oh, it's just a cold or the flu." They're just trying to come up with another reason. Also, they try to wait it out because they have a job, they can't take off work without taking PTL, or just they're doing something else for their family. [nurse navigator (118)]
**Logistic constraints to accessing healthcare.**	**Societal pressure to seek non-medical approaches.**	**Disjointed clinical services.**	**Limited or variable insurance coverage.**

For peer review only - http://bmjopen.bmj.com/site/about/guidelines.xhtml

Even if I want to get somebody screened for lung cancer screening, they may have to travel to a hospital 30 minutes away and not have even the gas money to do that. If I want to get somebody who's screen diagnosed in for a biopsy and for specialty care, they're potentially looking at traveling an hour.' [provider (115)]	Sometimes patients will say, "well you know, God heals and God will heal me" or they'll say "I want to try an herbal natural remedy. [provider(113)]	Then with a diagnosis just sending her home with that order, I think was irresponsible. Somebody should have said, "Hey, do you know you have this on your lung", and nobody did. And even her primary care doc circled back with them and said, "Why didn't you discuss this with my patient?" And they said, "Oh well, we thought you would. [caregiver(111)]	Well, this is really being driven by the insurance company. Your doctor really making some of these decisions. The insurance company made it for them. And that doesn't fit with your "medical condition" at the moment [caregiver (110)].
**Avoiding medical care due to a previous encounter involving racial discrimination**.	**The patient's surroundings normalize tolerating symptoms.**	**Delays in obtaining appointments**.	**Policies and laws determining investment in healthcare**.
People have shared that they felt, perhaps, their expression of their pain level maybe wasn't taken as seriously, because there might be some unconscious biases on the part of the healthcare provider about what they think about Black people and their threshold for pain and tolerance. '[109]	I think sometimes people don't think things are serious because it's all perception. So, if you have a family member, who let's say, everybody in your household smokes and everybody complains of shortness of breath, you probably think that it's normal to be short of breath. [113]	So just figuring that out and the next step of scheduling an appointment having to get on the phone and wait to schedule an appointment. it's not as easy as getting on to your computer sometimes, depending on the doctor's office and the Health Care system.' [113]	it relates to what resources are available in your area. If you live in a rural community and you're the only doctor in that community and the nearest specialist is 2 hours or 45 minutes away that can have an effect on your referral process and your ability to refer at times [113]
**Cultural identity**.	**HCPs indulging in discriminatory practices**.	**Organizational demands on HCPs to see more patients.**	**Limited management using telehealth**.
But another reason that I find for our patients is that sometimes it is just simply cultural beliefs around certain things. They don't want to admit that they have a problem. I mean, that could be something as simple as they have a pain somewhere. You know, I'll just take Tylenol or I'm coughing I won't go to the doctor, or they'll say things like you know. [provider (113)]	So, at the doctor level and, again, across cultures, they see an African-American person and they may or may not be obese, but there's so much emphasis on "You need to lose weight. You need to exercise. You need to eat right." And again, might be not investigating the extent by which the drinking and the smoking. The focus has been on quantity and not as much quality care. [caregiver (119)]	So, if you have a new patient and someone telling you have to see patients every 15 minutes and you're at the end of the day or the middle of day wherever you are in your day and you're just trying to get through it. Sometimes, the provider can feel rushed and glazed over the patient's symptoms' [provider (113)]	he ended up going to get some antibiotics via telemedicine from a doctor. But I think I noticed more more persistent coughing. [caregiver (102)]

For peer review only - http://bmjopen.bmj.com/site/about/guidelines.xhtml

**Lack of health literacy**.	**Patients anticipating prejudice and discrimination**.		**Practitioner's inexperience**.
If there's no context of understanding of medicine, medical illness would be deemed as something that's annoying, like a cough. Patients not understanding risk factors for disease. And so, based on either smoking or family history or other people with those symptoms, not understanding the importance of when to seek care or not to seek care.' [provider (114)]	It was awful. I mean, it was terrible. I mean if he had taken the opportunity to send me for an x-ray and I had great insurance. So, it's not like I was like, hey, let's penny-pinch. Like, let's penny-pinching. I do not want to do this, or I do not want to do that." I followed all of the instructions that he had given me, down to the tee and it was never an option for him to send me for an x-ray. He just never opted to do that [patients (106)]		I truly believe that if you recognize something you order it in a timely manner, but if you don't recognize it, I think part of it is recognition of the problem if you don't recognize it, it can delay you'. [provider (113)]
**Not knowing which HCP to contact**.			**Complex health systems not designed for vulnerable patients**.
I have got to find a doctor and the system for finding a doctor is so messed up. You get no information on a doctor. You are given a list of names and you are supposed to pick one. Well, how am I supposed to pick them based on the name? That is the first barrier. [researcher (103)]			you have been in a hospital before. It is almost impossible to navigate a hospital because parking is difficult. Getting in the maze. Knowing where to go and where you cannot go is, it is difficult. So, these are all barriers that make me not want to pursue or to follow up on this thing [researcher (103)]

For peer review only - http://bmjopen.bmj.com/site/about/guidelines.xhtml

DISCUSSION

This study is one of the first to report on the suboptimal experiences of BAA patients along the lung cancer care pathway. These experiences were identified by BAA patients, caregivers, and HCPs. The study found that some patients delayed seeking care, while others had their concerns ignored or were not offered molecular testing despite qualifying for it. Factors contributing to these suboptimal experiences included difficulties with insurance coverage, provider unwillingness to conduct comprehensive testing, provider bias in recommending treatment, high healthcare costs, and a lack of healthcare facilities and qualified staff to provide necessary tests and support. The study captured firsthand the individual and caregiver experiences, which were corroborated by community advocates and some healthcare provider experiences, particularly those with the lived experience of being BAAs.

Our findings indicate that the majority of patients experienced delays in both the diagnosis and initiation of treatment. Many patients received treatment for other common diseases before tests revealed their lung cancer. These delays are consistent with the 2021 State of Lung Cancer (SOLC) report, which found that BAAs were 16% less likely to receive an early diagnosis and 7% more likely to go untreated compared to White Americans.(33) HCPs' actions in offering diagnostic testing play a critical role in patient experiences. Positive relationships with HCPs can motivate patients to seek care, but we found that delays were sometimes caused by patients' concerns about discrimination from providers who may not refer them for timely diagnosis and targeted treatment. The literature shows that BAA patients often perceive communication with physicians as less supportive, less collaborative, and less informative.(34) Our findings on communication and information sharing align with this, as we observed instances of critical information being withheld, findings being communicated in ways that were difficult for patients to understand, and information on available treatment options not being shared.

The factors we identified as contributing to delays in lung cancer diagnosis may also lead to poorer outcomes throughout the cancer care process.(35) Patients presenting at later stages of illness may experience delays for several reasons, such as limited health literacy and self-assessment of symptoms. Previous experiences with discrimination may also impact patients' decisions about seeking care. Additionally, patients have reported feeling that their providers did not consider their symptoms to be serious, resulting in the treatment of other possible infections instead of additional testing.(36, 37) On the other hand, Patients reported using various approaches to overcome the barriers they faced in their healthcare journey, including utilizing their knowledge of health systems, seeking information on diagnostic and treatment options, and engaging with their insurance companies and HCPs to access comprehensive tests and treatment options. This underscores the importance of patient empowerment in advocating for their rights with insurance companies and HCPs, as well as the significance of receiving sufficient information from healthcare providers to make informed decisions about their healthcare. Patient navigators may also assist patients and families in navigating the health system and overcoming socioeconomic challenges. Addressing racism and prejudices through multilevel strategies, including retraining doctors, engaging policymakers, and educating nonclinical staff on prejudice, systemic biases, and racism, is crucial.

For peer review only - http://bmjopen.bmj.com/site/about/guidelines.xhtml

Strengths of this study included leveraging the perspectives of lung cancer survivors, caregivers, and frontline HCPs to provide diverse, real-life insights. Robust frameworks, including the cancer diagnosis pathway and NIMHD research framework, were used to organize the findings. The voices of BAA patients were prioritized to provide transparent, authentic perspectives. Limitations included a highly educated, insured population and a small sample size, potentially missing the views of diverse and harder-to-reach patients. However, the study's in-depth exploration enabled us to capture genuine care pathway experiences. Future research should aim to collect experiences of disparity from a more representative population and develop community-led interventions targeting individual and systemic factors perpetuating disparity. Evidence-based research methods should also focus on collecting patient perspectives relevant to experiences of racism, prejudice, and perceived injustices.

CONCLUSION

This study revealed suboptimal experiences and factors contributing to the disparities in lung cancer care among BAAs. Delays in diagnosis and initiation of treatment were reported, and patients faced various barriers, including insurance coverage, provider bias, and lack of healthcare facilities. Patients' empowerment and engagement with HCPs and insurance companies were important in navigating these barriers. Multilevel strategies are needed to address racism and prejudices in the healthcare system. Future research should focus on developing culturally-tailored community-centered interventions and evidence-based initiatives to collect, analyze, and disseminate patient perspectives on experiences of racism and perceived injustices. Although there were limitations to the study, the triangulation of perspectives and the use of robust frameworks strengthened the findings.

For peer review only

CONTRIBUTORSHIP STATEMENT

The listed authors, Nicholas Thuo (NT), Tanimola Martins (TM), Eugene Manley, Jr. (EMJ), Maisha Standifer (MS), Dawood H. Sulta (DHS), Nick Faris (NF) Angel Hill (AH), Matthew Thompson (MT), Rohan D. Jeremiah (RDJ), and Morhaf Al Achkar (MA) contributed to the conception, design, acquisition of data, or analysis and interpretation of data, drafting the article or critically revising it for important intellectual content, and final approval of the published version. NT and MA conceptualized this article and wrote the first draft along. NT, MA, TM, EMJ, NF, RDJ, MS, DHS, AH, and MT contributed to the revision and additional changes for the article to accurately reflect the research findings. MA affirms that the manuscript is an accurate representation of the issues covered, that there are no significant omissions or discrepancies. All authors have full responsibility for the work and decision to publish it..

COMPETING INTERESTS

All authors report no competing interests.

SOURCE OF FUNDING:

Funding: This research is linked to the CanTest Collaborative, which is funded by Cancer Research UK [C8640/A23385], of which author Matthew Thompson is associate directors and Co-investigator. The project was also partially funded by a gift from LUNGevity Foundation of which author Eugene Manley Jr., Director of STEM Workforce Initiatives. Information, conclusions and opinions expressed in this presentation are of the authors and no endorsement is intended or should be inferred.

DATA SHARING STATEMENT

The authors confirm that the data supporting the findings of this study are available within the article and its supplementary materials. The data will become available immediately after publication, with no end date for anyone who wishes access.

ETHICAL APPROVAL

Ethics approval was obtained from the University of Washington (UW) Human Subjects Division Reference number STUDY00005438.

For peer review only

BIBLIOGRAPHY

1. Ryan BM. Lung cancer health disparities. Carcinogenesis. 2018;39(6):741-51.
2. Soneji S, Tanner NT, Silvestri GA, Lathan CS, Black W. Racial and Ethnic Disparities in Early-Stage Lung Cancer Survival. Chest. 2017;152(3):587-97.
3. Robbins HA, Engels EA, Pfeiffer RM, Shiels MS. Age at cancer diagnosis for blacks compared with whites in the United States. Journal of the National Cancer Institute. 2015;107(3):dju489.
4. Xu G, Cui P, Zhang C, Lin F, Xu Y, Guo X, et al. Racial disparities in bone metastasis patterns and targeted screening and treatment strategies in newly diagnosed lung cancer patients. Ethnicity & health. 2022;27(2):329-42.
5. Society AC. Cancer Facts & Figures for African Americans 2022-2024. . 2022.
6. Surveillance E, and End Results (SEER) Program (www.seer.cancer.gov) SEER*Stat Database: Mortality - All COD, Aggregated With State, Total U.S. (1969-2017) <Katrina/Rita Population Adjustment>, National Cancer Institute, DCCPS, Surveillance Research Program, released December 2019. Underlying mortality data provided by NCHS (www.cdc.gov/nchs).
7. Lathan CS, Neville BA, Earle CC. The effect of race on invasive staging and surgery in non–small-cell lung cancer. Journal of Clinical Oncology. 2006;24(3):413-8.
8. Hardy D, Liu CC, Xia R, Cormier JN, Chan W, White A, et al. Racial disparities and treatment trends in a large cohort of elderly black and white patients with nonsmall cell lung cancer. Cancer. 2009;115(10):2199-211.
9. Bach PB, Cramer LD, Warren JL, Begg CB. Racial differences in the treatment of early-stage lung cancer. New England Journal of Medicine. 1999;341(16):1198-205.
10. Onega T, Duell EJ, Shi X, Demidenko E, Goodman DC. Race versus place of service in mortality among medicare beneficiaries with cancer. Cancer: Interdisciplinary International Journal of the American Cancer Society. 2010;116(11):2698-706.
11. Alvidrez J, Castille D, Laude-Sharp M, Rosario A, Tabor D. The national institute on minority health and health disparities research framework. American Journal of Public Health. 2019;109(S1):S16-S20.
12. Dempsey DA, Meyers MJ, Oh SS, Nguyen EA, Fuentes-Afflick E, Wu AH, et al. Determination of tobacco smoke exposure by plasma cotinine levels in infants and children attending urban public hospital clinics. Archives of pediatrics & adolescent medicine. 2012;166(9):851-6.
13. Haiman CA, Stram DO, Wilkens LR, Pike MC, Kolonel LN, Henderson BE, et al. Ethnic and racial differences in the smoking-related risk of lung cancer. New England Journal of Medicine. 2006;354(4):333-42.
14. Ho JT, Tyndale RF, Baker TB, Amos CI, Chiu A, Smock N, et al. Racial disparities in intensity of smoke exposure and nicotine intake among low-dependence smokers. Drug and Alcohol Dependence. 2021;221:108641.
15. Samet JM, Avila-Tang E, Boffetta P, Hannan LM, Olivo-Marston S, Thun MJ, et al. Lung cancer in never smokers: clinical epidemiology and environmental risk factors. Clinical Cancer Research. 2009;15(18):5626-45.
16. Hendryx M, O'Donnell K, Horn K. Lung cancer mortality is elevated in coal-mining areas of Appalachia. Lung Cancer. 2008;62(1):1-7.
17. Jonnalagadda S, Lin JJ, Nelson JE, Powell CA, Salazar-Schicchi J, Berman AR, et al. Racial and ethnic differences in beliefs about lung cancer care. Chest. 2012;142(5):1251-8.
18. Williams CD, Salama JK, Moghanaki D, Karas TZ, Kelley MJ. Impact of race on treatment and survival among US veterans with early-stage lung cancer. Journal of Thoracic Oncology. 2016;11(10):1672-81.
19. Zheng L, Enewold L, Zahm SH, Shriver CD, Zhou J, Marrogi A, et al. Lung cancer survival among black and white patients in an equal access health system. Cancer Epidemiology and Prevention Biomarkers. 2012;21(10):1841-7.

For peer review only - http://bmjopen.bmj.com/site/about/guidelines.xhtml

20. Brzezniak C, Satram-Hoang S, Goertz H-P, Reyes C, Gunuganti A, Gallagher C, et al. Survival and racial differences of non-small cell lung cancer in the United States Military. Journal of general internal medicine. 2015;30(10):1406-12.
21. Palazzo LL, Sheehan DF, Tramontano AC, Kong CY. Disparities and trends in genetic testing and erlotinib treatment among metastatic non–small cell lung cancer patients. Cancer Epidemiology and Prevention Biomarkers. 2019;28(5):926-34.
22. Li T, Kung H-J, Mack PC, Gandara DR. Genotyping and genomic profiling of non–small-cell lung cancer: implications for current and future therapies. Journal of Clinical Oncology. 2013;31(8):1039.
23. Kehl KL, Lathan CS, Johnson BE, Schrag D. Race, poverty, and initial implementation of precision medicine for lung cancer. JNCI: Journal of the National Cancer Institute. 2019;111(4):431-4.
24. Cote ML, Haddad R, Edwards DJ, Atikukke G, Gadgeel S, Soubani AO, et al. Frequency and type of epidermal growth factor receptor mutations in African Americans with non-small cell lung cancer. Journal of Thoracic Oncology. 2011;6(3):627-30.
25. Palazzo LL, Sheehan DF, Tramontano AC, Kong CY. Disparities and Trends in Genetic Testing and Erlotinib Treatment among Metastatic Non–Small Cell Lung Cancer PatientsGenetic Testing and Erlotinib Treatment. Cancer Epidemiology, Biomarkers & Prevention. 2019;28(5):926-34.
26. Lynch JA, Berse B, Rabb M, Mosquin P, Chew R, West SL, et al. Underutilization and disparities in access to EGFR testing among Medicare patients with lung cancer from 2010–2013. BMC cancer. 2018;18(1):1-13.
27. Bruno DS, Hess LM, Li X, Su EW, Zhu YE, Patel M. Racial disparities in biomarker testing and clinical trial enrollment in non-small cell lung cancer (NSCLC). Wolters Kluwer Health; 2021.
28. Harrison S, Judd J, Chin S, Ragin C. Disparities in lung cancer treatment. Current oncology reports. 2022;24(2):241-8.
29. Campbell JD, Lathan C, Sholl L, Ducar M, Vega M, Sunkavalli A, et al. Comparison of prevalence and types of mutations in lung cancers among black and white populations. JAMA oncology. 2017;3(6):801-9.
30. Alhazmi AA, Kaufmann A. Phenomenological qualitative methods applied to the analysis of cross-cultural experience in novel educational social contexts. Frontiers in Psychology. 2022;13:785134.
31. Walter F, Webster A, Scott S, Emery J. The Andersen Model of Total Patient Delay: a systematic review of its application in cancer diagnosis. Journal of health services research & policy. 2012;17(2):110-8.
32. Al Achkar M, Suchsland MZ, Walter FM, Neal RD, Goulart BH, Thompson MJ. Experiences along the diagnostic pathway for patients with advanced lung cancer in the USA: a qualitative study. BMJ open. 2021;11(4):e045056.
33. American Lung Cancer Association. State of lung cancer 2021. 2021.
34. Gordon HS, Street Jr RL, Sharf BF, Kelly PA, Souchek J. Racial differences in trust and lung cancer patients' perceptions of physician communication. Journal of clinical oncology. 2006;24(6):904-9.
35. Zuniga PVS, Ost DE. Impact of delays in lung cancer treatment on survival. Chest. 2021;160(5):1934-58.
36. Lubuzo B, Ginindza T, Hlongwana K. Exploring barriers to lung cancer patient access, diagnosis, referral and treatment in Kwazulu-Natal, South Africa: The health providers' perspectives. Translational Lung Cancer Research. 2019;8(4):380.
37. Carter-Harris L, Hermann CP, Draucker CB. Pathways to a lung cancer diagnosis. Journal of the American Association of Nurse Practitioners. 2015;27(10):576-83.

For peer review only - http://bmjopen.bmj.com/site/about/guidelines.xhtml

Figure1. suboptimal experiences throughout the diagnosis pathway.

For peer review only

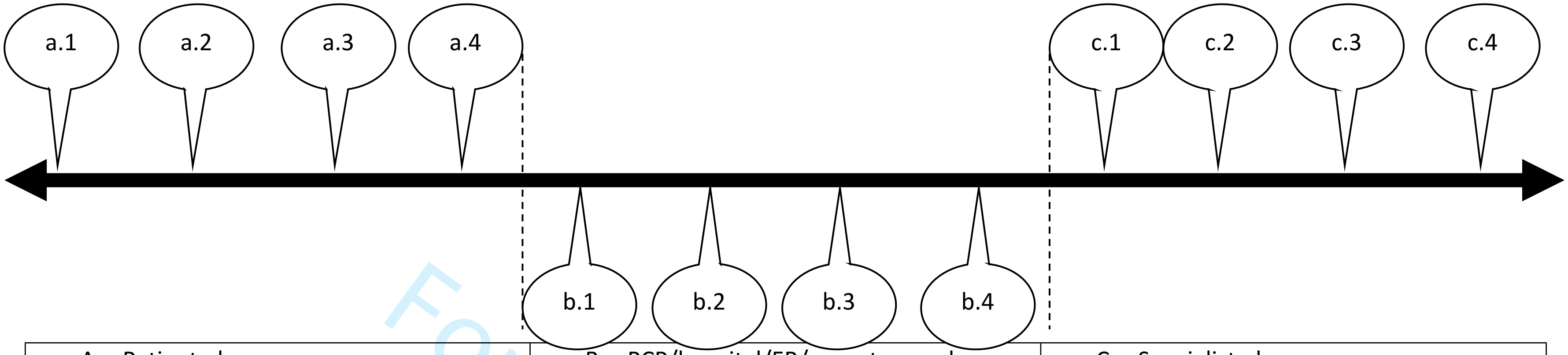

A. Patient phase	B. PCP/hospital/ER/urgent care phase	C. Specialist phase
a.1. Not being alarmed by the symptoms as they appeared	b.1. Being treated for common, more benign conditions	c.1. Difficulty finding an appointment to see the specialist
a.2. Explaining symptoms as caused by other conditions	b.2. Not offered diagnostic imaging	c.2. Poor delivery of information by the provider
a.3. Prolonging self-managing of symptoms	b.3. Not being informed of abnormal findings on tests	c.3. Not offered indicated molecular testing
a.4. Seeking care only as symptoms becamse severe	b.4. Poor communication by providers leaving the patient with with uncertainty	c.4. Steering patients to receive unnecessary chemotherapy

For peer review only

Appendix 1. Interview guides.

For peer review only

Semi-Structured Interview Guide

The study has two aims: Aim 1: Explore pitfalls, suboptimal experiences, and discriminatory practices in cancer diagnosis pathways for African Americans with metastatic lung cancer and oncogenic alterations. Aim 2: Identify etiologies of health disparity in cancer diagnosis for African Americans with metastatic lung cancer.

Cancer diagnosis

a. Tell me about your cancer diagnosis story, starting from when you noticed your first symptoms.

b. Patient phase
  i. What symptoms did you have that led to the cancer diagnosis?
  ii. How did the symptoms change over time before diagnosis?
  iii. Did anyone else notice your symptoms?
  iv. What did you think was the cause of your symptoms?
  v. How concerned were you about your symptoms and why?
  vi. What prompted you to seek medical care the first time?
  vii. Where did you seek care for the first time? Who else did you see?
  viii. How long do you think your symptoms were present before you sought care?
  ix. In your opinion, what contributed to that time (delay) between symptoms starting and seeking medical care?

c. PCP phase
  i. Describe your first interaction with your healthcare providers when you presented with symptoms.
  ii. What took place during the visit?
  iii. How did the provider explain your symptoms?
  iv. What were the steps taken to investigate your symptoms?
  v. What was the follow-up plan?
  vi. Were you offered any treatment for your symptoms? What was it?
  vii. Were you referred anywhere else? To whom?
  viii. How long after your doctor's visit did you get your first imaging test?
  ix. How was the decision made to get an imaging test?
  x. How were the findings on the imaging tests explained to you?
  xi. Was there any other tests ordered? What are they?
  xii. How much wait time did you have between your doctor's visit and getting the tests?
  xiii. In your opinion, what contributed to that time (delay) between seeking medical care and getting imaging tests?

d. Specialty care phase
  i. How was the decision made that you should see a specialist?
  ii. Describe the interactions with the specialists (pulmonologist, surgeon, radiologist, etc.) that led to getting a biopsy to confirm the diagnosis.
  iii. How were the results explained to you?
  iv. What other tests or procedures did you have early on?
  v. Share about your first interaction with the oncologist.
  vi. How much wait time did you have between receiving the cancer diagnosis and seeing an oncologist?
  vii. How was the *diagnosis* explained to you? What did they say about the disease you have?

For peer review only

viii. How was the *prognosis* explained to you? What did they say about what to expect will happen?
ix. What did they explain about the treatment options?
x. How was the decision made to get molecular testing?
xi. How were the tests explained to you?
xii. How were the results explained to you?
xiii. What treatment did you receive first?
xiv. How was the decision made to get a specific treatment?
xv. What other treatments have you received?
xvi. How much wait time did you have between your oncologist visit and starting treatment?

e. Before cancer
   i. How was your health before cancer?
   ii. Share about your lifestyle before cancer.
   iii. Have you smoked? If so, have you been offered smoking cessation?
   iv. How much did you smoke? If significant amount, have you been offered lung cancer screening?
   v. Have you had exposure to any carcinogen?
   vi. Share about encounters with healthcare before cancer.
   vii. What health issues did you deal with before cancer?
   viii. Did you have a PCP? Tell me about them? if not, why?

f. Reflections on etiologies of potential delays
   i. You mentioned, [example of possible inadequate/delay/suboptimal], from your vantage point, why do you think this happened?
   ii. What can be done to avoid such pitfalls from happening to other people who maybe in similar positions to yours?
   iii. (rephrase questions of focus group to ask patients/caregivers to identify factors and structures contributed to less than optimal outcomes for them or other people in their situation?)

Demographics

- What is your age?
  - Under 18
  - 18-24
  - 25-44
  - 45-64
  - Over 65
- What is your gender?
  - Male
  - Female
  - Other (please specify)
  - Prefer not to say
- What is your race/ethnicity?
  - White
  - Hispanic/Latino
  - Black/African American
  - Native American/American Indian
  - Asian/Pacific Islander
  - Other

For peer review only

- What is the highest level of school you've completed?
  - Less than a high school diploma
  - High school diploma or equivalent
  - Some college
  - Bachelor's degree
  - Master's degree
  - Doctorate
  - Other (please specify)
- What is your current employment status?
  - Employed Full-time (40 hours per week)
  - Employed Part-time (Less than 40 hours per week)
  - Unemployed
  - Student
  - Retired
  - Self-employed
  - Unable to work
- What is your home zip code?
- What is your marital status?
  - Single (Never married)
  - Married
  - Divorced
  - Widowed
- What is your household income?
  - Below $10K
  - $10K – $50K
  - $50K – $100K
  - $100K - $150K
  - Over $150K
- Cancer date of diagnosis:____
- Type of cancer:____
- Stage at time of diagnosis:____
- Stage now if not IV at time of diagnosis:____

For peer review only

Health Care Providers and Community Advocates

- We define cancer health disparity as the health difference, on the basis of one or more health outcomes, that adversely affects black/african americans. Specifically, We are interested in Identifying etiologies of health disparity around cancer diagnosis for African Americans with advanced lung cancer and oncogenic alterations.
- We want you help understanding the factors that cause this disparity. We are using the NIMHD model for health disparity. According to this framework, we examining the issue across 5 domains and 4 levels. See figure.

		Levels of Influence*			
		Individual	Interpersonal	Community	Societal
Domains of Influence (*Over the Lifecourse*)	**Biological**	Biological Vulnerability and Mechanisms	Caregiver–Child Interaction Family Microbiome	Community Illness Exposure Herd Immunity	Sanitation Immunization Pathogen Exposure
	**Behavioral**	Health Behaviors Coping Strategies	Family Functioning School/Work Functioning	Community Functioning	Policies and Laws
	**Physical/Built Environment**	Personal Environment	Household Environment School/Work Environment	Community Environment Community Resources	Societal Structure
	**Sociocultural Environment**	Sociodemographics Limited English Cultural Identity Response to Discrimination	Social Networks Family/Peer Norms Interpersonal Discrimination	Community Norms Local Structural Discrimination	Social Norms Societal Structural Discrimination
	**Health Care System**	Insurance Coverage Health Literacy Treatment Preferences	Patient–Clinician Relationship Medical Decision-Making	Availability of Services Safety Net Services	Quality of Care Health Care Policies
**Health Outcomes**		Individual Health	Family/ Organizational Health	Community Health	Population Health

This is the cancer diagnosis pathway.

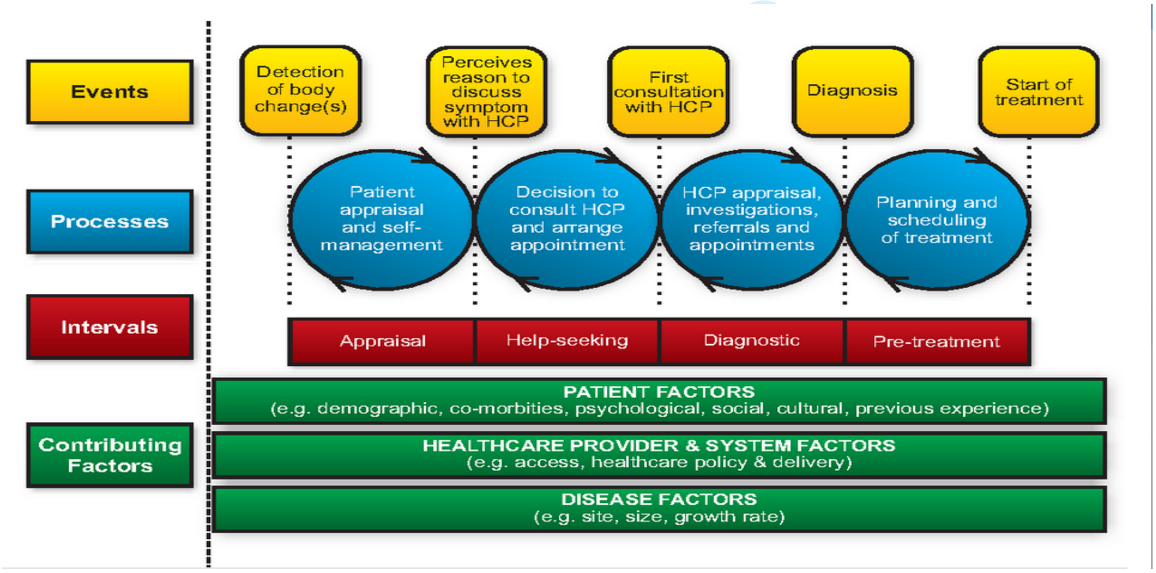

1. What are factors and structures that lead to delays in detecting first symptoms?

2. What are factors and structures that lead to inappropriate appraisal of symptoms?
3. What are factors and structures that lead to spending longer time in self-management phase as opposed to seeking help?
4. What are factors and structures that lead to not perceiving reasons to discuss symptoms with health care providers in a timely way?
5. What are factors and structures that lead to delays in consulting health care providers?
6. What are factors and structures that lead to not being able to arrange for appointments?
7. What are factors that lead to inadequate appraisal of symptoms by the health care provider on early visits?
8. What are factors and structures that lead to not ordering appropriate investigations in a timely manner?
9. What are factors and structures that lead to not arranging for referrals once suspicion of cancer become high (i.e., delays in getting to be seen by a specialist)?
10. What are factors and structures that lead to failing to obtain molecular testing
11. What are factors and structures that lead to not getting molecular testing?
12. What are factors and structures that lead to delays in getting molecular testing?
13. What are factors and structures that lead to delays in starting appropriate targeted therapy?

Caregiver's interviews are similar but rephrased to get the perspectives of the significant other on the health/experience of the patient.

Demographics

- What is your age?
  - Under 18
  - 18-24
  - 25-44
  - 45-64
  - Over 65
- What is your gender?
  - Male
  - Female
  - Other (please specify)
  - Prefer not to say
- What is your race/ethnicity?
  - White
  - Hispanic/Latino
  - Black/African American
  - Native American/American Indian
  - Asian/Pacific Islander
  - Other
- What is the highest level of school you've completed?
  - Less than a high school diploma
  - High school diploma or equivalent
  - Some college

For peer review only

  - Bachelor's degree
  - Master's degree
  - Doctorate
  - Other (please specify)
- What is your current employment status?
  - Employed Full-time (40 hours per week)
  - Employed Part-time (Less than 40 hours per week)
  - Unemployed
  - Student
  - Retired
  - Self-employed
  - Unable to work
- What is your home zip code?
- What is your marital status?
  - Single (Never married)
  - Married
  - Divorced
  - Widowed
- What is your household income?
  - Below $10K
  - $10K – $50K
  - $50K – $100K
  - $100K - $150K
  - Over $150K

For peer review only

Standards for Reporting Qualitative Research (SRQR)*

http://www.equator-network.org/reporting-guidelines/srqr/

Title and abstract

	Page/line no(s).
**Title** - Concise description of the nature and topic of the study Identifying the study as qualitative or indicating the approach (e.g., ethnography, grounded theory) or data collection methods (e.g., interview, focus group) is recommended	1
**Abstract** - Summary of key elements of the study using the abstract format of the intended publication; typically includes background, purpose, methods, results, and conclusions	3

Introduction

**Problem formulation** - Description and significance of the problem/phenomenon studied; review of relevant theory and empirical work; problem statement	5
**Purpose or research question** - Purpose of the study and specific objectives or questions	6

Methods

**Qualitative approach and research paradigm** - Qualitative approach (e.g., ethnography, grounded theory, case study, phenomenology, narrative research) and guiding theory if appropriate; identifying the research paradigm (e.g., postpositivist, constructivist/ interpretivist) is also recommended; rationale**	6
**Researcher characteristics and reflexivity** - Researchers' characteristics that may influence the research, including personal attributes, qualifications/experience, relationship with participants, assumptions, and/or presuppositions; potential or actual interaction between researchers' characteristics and the research questions, approach, methods, results, and/or transferability	7
**Context** - Setting/site and salient contextual factors; rationale**	6
**Sampling strategy** - How and why research participants, documents, or events were selected; criteria for deciding when no further sampling was necessary (e.g., sampling saturation); rationale**	6
**Ethical issues pertaining to human subjects** - Documentation of approval by an appropriate ethics review board and participant consent, or explanation for lack thereof; other confidentiality and data security issues	6
**Data collection methods** - Types of data collected; details of data collection procedures including (as appropriate) start and stop dates of data collection and analysis, iterative process, triangulation of sources/methods, and modification of procedures in response to evolving study findings; rationale**	6

For peer review only - http://bmjopen.bmj.com/site/about/guidelines.xhtml

For peer review only

**Data collection instruments and technologies** - Description of instruments (e.g., interview guides, questionnaires) and devices (e.g., audio recorders) used for data collection; if/how the instrument(s) changed over the course of the study	6
**Units of study** - Number and relevant characteristics of participants, documents, or events included in the study; level of participation (could be reported in results)	7
**Data processing** - Methods for processing data prior to and during analysis, including transcription, data entry, data management and security, verification of data integrity, data coding, and anonymization/de-identification of excerpts	7
**Data analysis** - Process by which inferences, themes, etc., were identified and developed, including the researchers involved in data analysis; usually references a specific paradigm or approach; rationale**	7
**Techniques to enhance trustworthiness** - Techniques to enhance trustworthiness and credibility of data analysis (e.g., member checking, audit trail, triangulation); rationale**	7

Results/findings

**Synthesis and interpretation** - Main findings (e.g., interpretations, inferences, and themes); might include development of a theory or model, or integration with prior research or theory	7-11
**Links to empirical data** - Evidence (e.g., quotes, field notes, text excerpts, photographs) to substantiate analytic findings	7 (tables)

Discussion

**Integration with prior work, implications, transferability, and contribution(s) to the field** - Short summary of main findings; explanation of how findings and conclusions connect to, support, elaborate on, or challenge conclusions of earlier scholarship; discussion of scope of application/generalizability; identification of unique contribution(s) to scholarship in a discipline or field	11-12
**Limitations** - Trustworthiness and limitations of findings	

Other

**Conflicts of interest** - Potential sources of influence or perceived influence on study conduct and conclusions; how these were managed	13
**Funding** - Sources of funding and other support; role of funders in data collection, interpretation, and reporting	13

*The authors created the SRQR by searching the literature to identify guidelines, reporting standards, and critical appraisal criteria for qualitative research; reviewing the reference lists of retrieved sources; and contacting experts to gain feedback. The SRQR aims to improve the transparency of all aspects of qualitative research by providing clear standards for reporting qualitative research.

For peer review only

**The rationale should briefly discuss the justification for choosing that theory, approach, method, or technique rather than other options available, the assumptions and limitations implicit in those choices, and how those choices influence study conclusions and transferability. As appropriate, the rationale for several items might be discussed together.

Reference:

O'Brien BC, Harris IB, Beckman TJ, Reed DA, Cook DA. **Standards for reporting qualitative research: a synthesis of recommendations.** *Academic Medicine*, Vol. 89, No. 9 / Sept 2014 DOI: 10.1097/ACM.0000000000000388

For peer review only

BMJ Open

Factors Leading To Disparity In Lung Cancer Diagnosis Among Black/ African American Communities in the United States: A Qualitative Study

Journal:	*BMJ Open*
Manuscript ID	bmjopen-2023-073886.R1
Article Type:	Original research
Date Submitted by the Author:	23-Aug-2023
Complete List of Authors:	Thuo , Nicholas; University of Washington Seattle Campus<br>Martins, Tanimola; University of Exeter, Primary Care; University of Exeter , Primary Care<br>Manley Jr. , Eugene ; LUNGevity Foundation<br>Standifer, Maisha ; Morehouse School of Medicine<br>Sultan , Dawood ; Mercer University<br>Faris , Nick ; Baptist Memorial Health Care Corp<br>Hill , Angela ; University of South Florida<br>Thompson, Matthew; University of Washington, Department of Family Medicine<br>Jeremiah, Rohan ; University of Illinois at Chicago,<br>Al Achkar, Morhaf; University of Washington, Family Medicine ;
**Primary Subject Heading**:	Oncology
Secondary Subject Heading:	Public health
Keywords:	Public health < INFECTIOUS DISEASES, Adult oncology < ONCOLOGY, Respiratory tract tumours < ONCOLOGY

SCHOLARONE™
Manuscripts

BMJ

I, the Submitting Author has the right to grant and does grant on behalf of all authors of the Work (as defined in the below author licence), an exclusive licence and/or a non-exclusive licence for contributions from authors who are: i) UK Crown employees; ii) where BMJ has agreed a CC-BY licence shall apply, and/or iii) in accordance with the terms applicable for US Federal Government officers or employees acting as part of their official duties; on a worldwide, perpetual, irrevocable, royalty-free basis to BMJ Publishing Group Ltd ("BMJ") its licensees and where the relevant Journal is co-owned by BMJ to the co-owners of the Journal, to publish the Work in this journal and any other BMJ products and to exploit all rights, as set out in our licence.

The Submitting Author accepts and understands that any supply made under these terms is made by BMJ to the Submitting Author unless you are acting as an employee on behalf of your employer or a postgraduate student of an affiliated institution which is paying any applicable article publishing charge ("APC") for Open Access articles. Where the Submitting Author wishes to make the Work available on an Open Access basis (and intends to pay the relevant APC), the terms of reuse of such Open Access shall be governed by a Creative Commons licence – details of these licences and which Creative Commons licence will apply to this Work are set out in our licence referred to above.

Other than as permitted in any relevant BMJ Author's Self Archiving Policies, I confirm this Work has not been accepted for publication elsewhere, is not being considered for publication elsewhere and does not duplicate material already published. I confirm all authors consent to publication of this Work and authorise the granting of this licence.

For peer review only

Factors Leading To Disparity In Lung Cancer Diagnosis Among Black/ African American Communities in the United States: A Qualitative Study

Nicholas Thuo[1], Tanimola Martins[2], Eugene Manley, Jr.[3], Maisha Standifer[4], Dawood H. Sultan[5], Nick Faris[6], Angela Hill[7], Matthew Thompson [1], Rohan D. Jeremiah[8], Morhaf Al Achkar[1]

1. Department of Family Medicine, University of Washington, Seattle, WA;

2. University of Exeter Medical School

3. LUNGevity Foundation, Chicago, IL;

4. Morehouse School of Medicine, Satcher Health Leadership Institute, Atlanta, GA

5. Mercer University, Atlanta, Georgia

6. Baptist Memorial Health System, Memphis, TN;

7. University of South Florida, Tampa, FL;

8. University of Illinois Chicago, College of Nursing, Chicago, IL;

Corresponding author

Morhaf Al Achkar

Department of Medicine, University of Washington, Seattle, WA;

Tel: +2068009480

Email: alachkar@uw.edu

Keywords: Lung cancer, disparity, sub-optimal experiences, NIMHD model for health disparity

For peer review only - http://bmjopen.bmj.com/site/about/guidelines.xhtml

Abstract word count: 284

Word Count: 3471

Tables: 3

Figures: 1

References: 38

For peer review only

For peer review only - http://bmjopen.bmj.com/site/about/guidelines.xhtml

ABSTRACT

Objective: This study has two objectives: first, to explore the diagnostic experiences of Black/African American (BAA) patients with lung cancer to pinpoint pitfalls, suboptimal experiences, and instances of discrimination leading to disparities in outcomes. The second objective is to identify the underlying causes contributing to health disparities in the diagnosis of lung cancer among African American patients.

Methods: We conducted in-depth interviews with individuals knowledgeable about BAA diagnosed with lung cancer. Participants included patients, caregivers, advocates, and healthcare providers. We performed thematic analysis to identify experiences at patient, primary care and specialist levels. Contributing factors were identified using the National Institute of Minority Health and Health Disparities (NIHMD) health disparity model.

Results: From March to November 2021, we conducted individual interviews with 19 participants, including 9 patients/caregivers and 10 providers/advocates. The median age group of participants was 45-64 years. Participants reported recurring and increased pain before seeking treatment, treatment for non-cancer illnesses, delays in diagnostic tests and referrals, poor communication and bias when dealing with specialists and primary care providers. Factors contributing to suboptimal experiences included reluctance by insurers to cover costs, provider unwillingness to conduct comprehensive testing, provider bias in recommending treatment, high healthcare costs, and lack of healthcare facilities and qualified staff to provide necessary support. However, some participants reported positive experiences due to their insurance, availability of services, and having an empowered support structure.

Conclusions: Suboptimal experiences were remarkable among BAA patients and caregivers, and the NIMHD model is a useful framework to organize factors contributing to these experiences that maybe leading to health disparities. Additional research is needed to fully capture the extent of these experiences and identify ways to improve African American experiences in the lung cancer diagnosis pathway.

For peer review only

STRENGTHS AND LIMITATIONS OF THIS STUDY

- Strengths of this study included leveraging the perspectives of lung cancer survivors, caregivers, and frontline HCPs to provide diverse, real-life insights.
- Robust frameworks, including the cancer diagnosis pathway and NIMHD research framework, were used to organize the findings.
- The voices of BAA patients were prioritized to provide transparent, authentic perspectives.
- Limitations included a highly educated, insured population and a small sample size, potentially missing the views of diverse and underserved patients.

KEY MESSAGES

What is already known on this topic:

Black/African Americans have the highest morbidity and mortality rates for most cancers compared to any other racial group. Addressing the root causes of sub-optimal experiences for African Americans in cancer diagnosis pathways can help mitigate such disparities.

What this study adds:

Our study delves into the experiences of lung cancer patients, caregivers, and healthcare providers along the diagnostic and treatment pathways, highlighting the factors contributing to sub-optimal experiences.

How this study might affect research, practice, or policy:

A deeper understanding of these experiences and the factors influencing them could lead to positive changes in provider-patient interactions, identify areas for improvement in provider training and health systems, and improve cancer diagnosis and treatment pathways for African Americans.

For peer review only - http://bmjopen.bmj.com/site/about/guidelines.xhtml

INTRODUCTION

Lung cancer disproportionately affects Black/African American (BAA) communities.(1-5) In 2022, BAA had a lower estimated 5-year survival rate (20%) compared to Whites (22%).(5) BAA have a lower rate of early-stage detection for lung cancer, at only 16% (compared to 20% among all races), with 53% being diagnosed at distant locations (compared to 49% for non-Hispanic Whites). (5) BAAs are less likely than patients from other racial/ethnic groups to be staged during diagnosis, and to receive curative treatment at the most optimal point in the course of the disease. (2, 6, 7) These disparities appear to be unrelated to health insurance, as BAA Medicare beneficiaries with lung cancer experience higher mortality rates than non-Hispanic Whites.(8)

The NIMHD research framework offers guidance on considering the domains and levels of influence of factors that may account for why lung cancer disproportionately affects BAAs.(9) Differences in nicotine metabolism, which are linked to genetics and heredity, have been identified as a potential biological vulnerability.(1, 10, 11). Documented behavioral factors include smoking patterns, exposure to secondhand smoke, and care avoidance due to beliefs and fears.(1, 12) Environmental factors, including greater exposure to radon and indoor pollutants, living near smelting factories, and working in coal mines, have been identified as potential contributing factors to these disparities.(13) Lower educational attainment, rurality, poverty, and limited access to healthcare due to employment are significant implicated sociocultural factors.(14-16) Research further indicates that systemic racism leading to inequitable access to quality healthcare is the critical factor for the gap in outcomes when a BAA develops lung cancer.(17, 18)

Routine use of molecular testing and targeted cancer therapy for late-stage non-small cell lung cancer (NSCLC) has significantly reduced mortality rates.(19, 20) Patients who receive molecular testing within 60 days of diagnosis have better survival rates compared to those who do not.(21) Studies have found that BAAs have similar rates of oncogenic alterations as non-Hispanic Whites and at least one in every three BAA patients have an actionable mutation.(22) Nevertheless, BAAs tend to receive molecular testing or targeted treatment less frequently than Whites.(21) Even in Medicare populations, compared to non-Hispanic Whites, BAA patients over 65 years old with NSCLC had lower odds ratios of 0.53-0.63 for receiving targeted therapy.(23, 24) White patients with NSCLC were more likely to receive next-generation sequencing, compared to BAA patients (50.1% vs 39.8%, $p<0.0001$).(25) Limited access to testing among BAA patients may be contributing to disparities in outcomes.(26) Studies have shown that when given access to care, BAA lung cancer patients have the same outcomes as White patients.(22, 27)

Extensive literature exists on the roots of disparities and how to eliminate them for patients with early-stage lung cancers.(2, 28-30) However, little is known about the etiologies of disparity for patients with late-stage lung cancer. While it is possible that etiologies of disparities in treating metastatic diseases share some commonalities with early-stage cancers, they may also have unique attributes contributing to the complex and long-term treatment paradigm of metastatic

For peer review only - http://bmjopen.bmj.com/site/about/guidelines.xhtml

diseases. This study aimed to achieve two objectives: the first was to explore the diagnostic pathways for BAAs with lung cancer to identify pitfalls, suboptimal experiences, and discriminatory practices occurred. The second objective was to identify the etiologies of health disparity in cancer diagnosis for BAAs with lung cancer.

METHODS

Study Setting, Population, and Design

We utilized a phenomenological research design to guide qualitative interviews conducted with patients, caregivers, healthcare professionals, and community advocates.(31) Patient and caregiver interviews provided patient-level accounts to explore experiences and identify diagnostic pathways and challenges encountered, from which we identified suboptimal experiences during the diagnostic phase. Healthcare professional and community advocate interviews produced reflections and observations to contextualize possible causes of observed challenges for individuals in BAA communities. The study was co-designed and conducted with the help of a community advisory group (CAG) called Project RADICAL - Racial DIsparities in CAncer of the Lung - formed in 2020. The CAG, consisting of patients, caregivers, lung cancer researchers, and community advocates, met biweekly to develop the study protocol, inform study methods and interview guides, and interpret findings. The University of Washington's Institutional Review Board approved the study.

Patient and Public Involvement

We used a purposive sampling strategy to identify participants who met the inclusion criteria, which included patients aged 18 years or older who self-identified as BAA and had been diagnosed with metastatic lung cancer in the United States. Caregivers were individuals providing primary care for a BAA lung cancer patient. Healthcare providers (HCPs) and community advocates were practicing physicians providing oncology or primary care services, or advocates for BAA communities. To identify participants, we used snowball sampling methods through the community network of Project RADICAL CAG members, collaborators' networks in cancer organizations, online support groups such as ALK-positive, and academic list servers focused on disparities.

Data Collection

The research team developed the interview guides using the Anderson's model of pathways to cancer treatment.(32) Based on the findings of our previous work(31), the model was refined to organize patients' diagnostic experiences into three phases: 1) patient phase (from first symptom recognition to the first doctor's visit in primary care), 2) primary care phase (from the first primary care visit to presumptive diagnosis based on imaging), and 3) secondary care phase (from referrals to biopsy until identifying actionable genes and starting treatment).(33) The patient and caregiver interviews began with open-ended questions about their experiences with a cancer diagnosis. HCP and community advocate interviews aimed to identify factors contributing to suboptimal experiences along the diagnosis pathway (see Appendix 1 for interview guides). An experienced qualitative researcher (MA) conducted all interviews via phone or video

For peer review only - http://bmjopen.bmj.com/site/about/guidelines.xhtml

conference, with most conducted via Zoom. Interviews were audio recorded and transcribed verbatim. Participants received a $50 gift card for participating in the 30-60 minute interviews.

Analysis

An experienced qualitative researcher (NT) coded the interviews using Dedoose (Dedoose Inc., Manhattan Beach, California) for analysis. The senior author (MA) provided peer debriefing and supervised NT's coding. Transcripts were analysed using inductive and deductive strategies, with codes developed highlighting suboptimal experiences and factors leading to disparity. The NIMHD research framework was used to organize the emerging factors contributing to disparity, and patient and caregiver narratives were triangulated with community advocates and HCPs' reflections. Findings were presented to the CAG and co-authors, and inputs were used to iteratively develop themes.

Researchers' Characteristics and Reflexivity

The research team brings diverse backgrounds, experiences, and perspectives to this qualitative study. MA is a male, PhD-trained qualitative researcher, identifying as an Arab immigrant, with personal experience living with advanced lung cancer. MS is a female, PhD-trained qualitative researcher, identifying as BAA, and has served as a caregiver for a BAA patient who succumbed to advanced lung cancer. NT, an MPH-trained and experienced qualitative researcher, identifies as Black African from Kenya. TM is a male, PhD-trained qualitative researcher, identifying as Black African from Nigeria. RDJ is a male, PhD-trained researcher with expertise in qualitative research, identifying as BAA. DHS is a male, PhD-trained health service researcher, identifying as BAA. NF holds a Master of Divinity and serves as a research manager and health equity leader; he identifies as a White male. AH is a female, Pharm.D with experience in disparity research, identifying as BAA, and has had a family member who died of advanced lung cancer. EMJ is a male, PhD-trained molecular and cell biologist and researcher, identifying as BAA, and has studied lung cancer therapeutics and is a patient advocate. MT is a male, PhD-trained family doctor and researcher, who has also experienced the loss of a family member to lung cancer.

RESULTS

The demographic characteristics of the participants are presented in Table 1. Participants were aged 25–64 years old, with 55% female,78% black, and 58% were patients or caregivers. At the time of the interview, 94% were in full-time employment. We will describe the themes that emerged in two main areas, namely suboptimal experiences and factors leading to disparities.

For peer review only

Table 1. Participants' demographics.

ID	Age Group	Gender	Race /Ethnicity	Education	Zipcode	Marital status	income (000 $)	Role
101	25-44	Female	black	Doctorate	11203	Single	<50	Patient
102	45-64	Female	Black	some college	33705	Married	50-100	Caregiver
103	45-64	Male	Black	Doctorate	46268	Married	100-150	Researcher
104	>65	Female	Black	Doctorate	60621	Divorced	50-100	Patient*
105	>65	Female	Black	Masters	60652	Married	50-100	Caregiver
106	25-44	Female	Black	Bachelors	10701	Single	100-150	Patient
107	45-64	Female	Black	Bachelors	46234	Married	100-150	Patient
108	25-44	Female	Black	Bachelors	46268	Married	50-100	Advocate
109	45-64	Male	Black	some college	91367	Single	50-100	Patient
110	45-64	Male	Black	Masters	46278	Married	100-150	caregiver
111	45-64	Female	Black	Doctorate	46214	Married	>150	caregiver
112	Unknown	Male	Black	Doctorate	Unknown	Married	>150	oncologist
113	25-44	Female	Black	Doctorate	31008	Married	>150	Provider
114	45-64	Female	Black	Doctorate	15146	Married	>150	Provider
115	45-64	Male	White	Doctorate	43452	Married	>150	Provider
116	45-64	Male	White	Doctorate	80111	Married	>150	Provider*
117	45-64	Male	White	Doctorate	98115	Married	>150	provider
118	45-64	Female	White	Master's	41042	Single	$50k-$100k	Nurse Navigator
119	45-64	Female	Black	Doctorate	33579	Married	>150	caregiver

* Indicates participants who were retired, all other participants were in full-time employment

For peer review only - http://bmjopen.bmj.com/site/about/guidelines.xhtml

Suboptimal experiences along the diagnosis pathways

We organized the suboptimal experiences within the patient phase, the PCP/hospital/ER/urgent care phase, and the specialist phase. Figure 1 depicts suboptimal experiences throughout the diagnosis pathway. Table 2 includes supportive quotes.

Patient Phase

- **Not being alarmed by the symptoms as they appeared.** Some patients did not seek care right away as they were not concerned about the symptoms either because of their mild nature or the failure to appraise their seriousness.
- **Explaining symptoms as caused by other conditions.** Patients and caregivers at times attributed symptoms to less serious health conditions, such as common cold and allergies, delaying seeking medication consultation.
- **Prolonging self-managing of symptoms.** Patients resorted to home remedies or over-the-counter treatment for cancer-related symptoms (e.g., cough and pain) instead of seeking medical care.
- **Seeking care only as symptoms became severe.** The threshold to seek medical advice was not crossed until symptoms were too serious to ignore or especially bothersome (e.g., severe pain).

PCP/Hospital/ER/Urgent Care Phase

- **Being treated for common, more benign conditions.** Patients frequently received treatment for more common and less serious conditions, such as sinus infections or acid reflux. Patients then ignored the symptoms without clear instructions or a follow-up plan.
- **Delayed or not being offered diagnostic imaging.** Some patients were not offered diagnostic tests due to the HCP's low levels of suspicion for lung cancer.
- **Not being informed of abnormal findings on tests.** Some patients were not informed about abnormal findings on their diagnostic tests and were left without any follow-up. On occasions, patients were not informed about the findings or the plan and they discovered them by reading the handouts.
- **Communicated information poorly and left with uncertainty.** Patients were kept uninformed regarding the plans of care. Sometimes they were not told how they could receive help if they needed urgent care or had questions.

Secondary Care Phase

- **Difficulty finding an appointment to see the specialist.** Some patients, especially in underserved areas, had difficulty finding timely appointments to see specialists.
- **Poor delivery of information by the HCP.** Some patients were hassled during their care, receiving sub-par communication or little guidance to make decisions.
- **Not receiving molecular testing.** At times, patients were not offered molecular testing—a standard of care—and as a result, missed an opportunity to receive novel treatments.

For peer review only - http://bmjopen.bmj.com/site/about/guidelines.xhtml

- **Feeling steered to receive unnecessary chemotherapy.** Some patients felt they were pushed to take the only option of chemotherapy, while other more-novel treatment options were not discussed.

For peer review only

Table 2. Supportive Quotes for The Suboptimal Experiences Along The Diagnosis Pathways

Patient phase	PCP/hospital/ER/urgent care phase	Specialist phase
**Not being alarmed by the symptoms as they appeared.**	**Being treated for common, more benign conditions.**	**Difficulty finding appointment to see the specialist.**
Time went on and cough never got better, but it did not seem to get worse. [patient (107)]	They'll say, "Hey, it's sinus or allergies," they'll give them an antibiotic. [Nurse navigator (118)]	If you live in a rural community and you're the only doctor in that community and the nearest specialist is 2 hours or 45 minutes away that can have an effect on your referral process and your ability to refer at times. [Provider (113)]
	My primary care doctor diagnosed me with having allergies as well as acid reflux. I was treated with Protonix. I was treated with Claritin, over-the-counter stuff and eventually I would just ignore it. [patient (107)]	
**Explaining symptoms as caused by other conditions.**	**Delayed or not being offered diagnostic imaging.**	**Poor delivery of information by the provider.**
They had similar symptoms before and they resolved on their own without seeking help or with conservative measures; may be that if they have ignored in the past that can lead to worst consequence for them. [Provider (106)]	I went to another doctor and the first thing the doctor asked me "had you had an x-ray?" I was like, "No. Nobody sent me for an x-ray." [Patient (106)]	It was just another bad experience where I was sitting in the doctors for my appointment. He was perpetually on the telephone talking to other people as I was sitting there and then kind of squeezing in like when he was on hold or between calls telling me something really quick. [Patient (109)]
She had a cough for quite a while and we thought that it was allergies. Then, unfortunately, she was in a head-on collision with a drunk driver and it broke the transverse process of her C7 vertebrae. [Caregiver (104)]	You can't do chest CTs in every person, that's not a reasonable approach. But I think that's, in part, where some diagnostic delays happen is that perhaps they didn't have a high enough pretest probability to do the subsequent downstream test. [Provider (117)]	I would think knowing that I am also in a medical field that you would have at least tried to explain to me. I did not think that I should have had to be like, I'm not leaving until somebody explains what happens. I do not think that I should have had to do that. [Patient (101)]
**Prolonging self-managing of symptoms.**	**Not being informed of abnormal findings on tests.**	**Not receiving molecular testing.**
He could not go to sleep at night so he would be taking NyQuil and it was more of a consistent thing. [caregiver (102)]	When I looked at that hospital discharge summary and saw that there were lung nodules there. I had not seen that before or ever been told or followed up about that. [caregiver (102)]	I had one or two patients diagnosed with lung cancer who went to a smaller local facility for their follow-up, and I brought them back into the office to go through things. And I said, "Well, I don't see any molecular testing on your biopsy."

For peer review only - http://bmjopen.bmj.com/site/about/guidelines.xhtml

		You've lost that opportunity [Thoracic oncologist (115)]
**Seeking care only as symptoms became severe.**	**Communicated information poorly and left with uncertainty.**	**Feeling steered to receive unnecessary chemotherapy.**
One night I woke up because I felt like my left side of the chest was closing down on me, I felt that my left side was not getting enough air in. [Patient (101)]	Nobody talked to her about the mass, but she was reading her orders and she was like, "What is this in my orders?" [Caregiver (111)]	They were very pushy with the chemotherapy which made me even more skeptical. I was just saying, "Wait a minute. I would like a second opinion." I had texted the doctor and said, "they want to do chemo immediately." She said, "No, let us wait. I will get him in next week and we can do a genetic test that can see if chemo is the best for him or targeted medicine." That was the first time I had heard about targeted medicine. [Caregiver (102)]
	So who do I call? Which doctor's office do I call? If I'm having a problem, do I go to the ER? Do I go to Urgent Care? Do I go to the pulmonologist because my cousin goes to one? Or do I go to my primary care doctor? [Provider (113)]	

For peer review only - http://bmjopen.bmj.com/site/about/guidelines.xhtml

For peer review only

Factors Leading To Disparity In Lung Cancer Diagnosis

Herein, we describe the factors that lead to suboptimal experiences along the pathways to diagnosis and treatment for BAA patients. These factors work at multiple individual, interpersonal, community, and societal levels. Tables 3 includes supportive quotes.

Individual Factors

- **Not seeking timely care.** Some patients reported an aversion to seeking timely medical care for fear of finding serious illnesses or having to deal with adverse outcomes.
- **Inadequate reporting of relevant symptoms**. The patients' experiences with disease symptoms influence their decisions on which symptoms to report. They may not feel comfortable reporting certain symptoms and may understate the details.
- **Logistic constraints to accessing healthcare.** Patients living in poorer areas tend to have poorer healthcare outcomes due to affordability and income issues. At times, patients live far from the health facility and are unable to access transportation or assistance to travel for treatment.
- **Avoiding medical care due to a previous encounter involving racial discrimination**. Perceptions of the HCP based on previous experiences often lead to mistrust and hesitancy among patients to avoid possible dismissals or maltreatment.
- **Cultural identity**. People are often influenced by their community's behavior and religious beliefs regarding healthcare.
- **Lack of health literacy**. At times, patients are unable to appreciate the severity of a diagnosis or are unaware of what to do when they experience health changes.
- **Not knowing which HCP to contact**. Information on HCPs and their specialties are not readily available, making it hard for the patient to determine who to reach out to and where.

Interpersonal Factors

- **Lack of compassionate family support.** For some individuals, weaker family ties lead to socioeconomic isolation, which obscures the management of their healthcare needs.
- **Doctors' schedules not accommodating patients.** In certain areas, the doctors' offices or health facilities are only open during weekdays, when many people are at work and are therefore unable to access healthcare.
- **Societal pressure to seek non-medical approaches.** It is often observed that the patient's social networks encourage the use of alternative treatment methods, including herbal and non-regulated remedies. As a result, some patients may choose spiritual practices or herbs over medical care.
- **Patients' surroundings normalize tolerating symptoms.** Patients seldom internalize the need to endure the disease and fear the loss of dignity through their dependence on others, especially where the symptoms are common around them.
- **HCPs indulging in discriminatory practices**. It has been observed that HCPs tend to neglect, miss, or dismiss symptoms reported by BAA patients, especially pain. Furthermore, they may not execute appropriate referrals.

For peer review only - http://bmjopen.bmj.com/site/about/guidelines.xhtml

- **Patients anticipating prejudice and discrimination**. Patients are concerned about how HCPs may treat them based on their personal attributes, especially race. Others, especially individuals who used to smoke, avoid seeking medical care due to the fear of being blamed for their illness and the associated stigma of causing cancer.

Community Factors

- **Cultural practices concerning healthcare.** Certain therapies may be preferred in a community, for example, obtaining painkillers instead of identifying the cause of the ailment.
- **Availability of clinical services.** There is a dearth of hospitals, diagnostic facilities, primary care providers (PCPs), and specialists within certain communities and areas. This is especially true for smaller communities living in poor areas.
- **Disjointed clinical services**. Referrals and visits to different departments and hospitals may force patients to drop out of treatment and follow up on results. Furthermore, communicating the results to PCPs after visiting the emergency room is left to the patient and not communicated directly, which often leads to missed opportunities for follow-up on diagnosis and treatment.
- **Delays in obtaining appointments**. Although booking systems for hospitals are now online, navigating these systems is complex and coupled with organizational constraints in the availability of appropriate staff. A patient's diagnosis may be delayed due to a lack of appointment slots.
- **Organizational demands on HCPs to see more patients.** For profitable practice, some hospitals and clinics set patient and charge goals for HCPs, which leads to poor service quality. Also, forcing HCPs to attend to more patients within a stipulated time may overwhelm them, reducing their quality of care.

Societal Factors

- **Working in lower-paying jobs.** The relegation of certain communities to low-paying jobs limits their access to resources for managing health and disease impacts. This economic disempowerment leaves personal healthcare needs competing for priority with one's family's needs. Often, family commitments deny the patient the appropriate time to seek treatment.
- **Cost of receiving care**. Seeking diagnosis and treatment is costly for individuals, especially when they face job insecurity and can take limited time off from work. This issue, coupled with the inflated costs of medical care, makes healthcare services inaccessible or undesirable.
- **Limited or variable insurance coverage**. Limited options within Medicaid coverage, cost of insurance, and the inability to obtain insurance led to suboptimal access to timely diagnosis and treatment. Besides, the unwillingness of insurance companies to pay for certain services, either in full or part, may limit one's options for quality care.
- **Policies and laws determining investment in healthcare**. The existing policies and laws related to healthcare and resources available to communities affect access, education, and living conditions for the community.

For peer review only - http://bmjopen.bmj.com/site/about/guidelines.xhtml

- **Limited management using telehealth**. Only using telehealth and the absence of physical testing and comprehensive workups lead to missed diagnoses.
- **Practitioner's inexperience**. Underprivileged communities are often subjected to care by undertrained practitioners and a shortage of specialists. A lack of adequate skill and experience to manage chronic diseases may lead to lower suspicion or missing critical symptoms.
- **Complex health systems not designed for vulnerable patients**. Moving through complex online hospital systems and departments to book and access essential services requires internet access either by computer or phone, which can be challenging for many.

For peer review only

For peer review only - http://bmjopen.bmj.com/site/about/guidelines.xhtml

Table 3. Supportive Quotes for The Factors Leading to Disparity in Lung Cancer Diagnosis

Individual Factors	Interpersonal Factors	Community Factors	Societal Factors
**Not seeking timely care.**	**Lack of compassionate family support.**	**Cultural practices concerning health care.**	**Working in lower-paying jobs.**
Yeah. I mean, I think it's related to, "I hope that things will get better!" It can also be related to a form of denial So, in fact, they know it might be lung cancer and are not going in as a form of denial. [Provider (117)]	In the majority in a community, you're more likely to have a wider network within that community. If you have a cough, you're more likely to be talking to somebody about it and somebody said, " You should get it checked out." If you are more isolated within the community because of race or economic issues [you won't have] that network [provider (115)]	Some people take their symptoms more seriously than others. I don't know whether this is more or less among African Americans, but to the extent to which symptoms are minimized or ignored, that's more personal or cultural. [provider (117)]	A lot in African-American and Hispanic Communities do not have insurance, rather because they cannot afford healthcare insurance. Because they are making minimum wage jobs or it just not on the priority list of "I have never been sick [Advocate (108)]
**Inadequate reporting of relevant symptoms.**	**Doctors' schedules not accommodating patients.**	**Availability of clinical services.**	**Cost of receiving care**.
What's important to the patient and to the doctor are two different things. So, they may not consider their shortness of breath and issue because they've been having it over a period of time. [Provider (113)]	A lot of jobs, you know, the timing, like the doctor's offices, closes at five o'clock, and they normally take their last patient at 4.30. [advocate(108)]	I think it's also where is the setting of the low-dose CT scans. Most of them are in hospitals, or some patients just won't go to a hospital for care. [Provider (114)]	They try to wait it out because they have a job, they can't take off work without taking PTL, or just they're doing something else for their family. [nurse navigator (118)]
**Logistic constraints to accessing healthcare.**	**Societal pressure to seek non-medical approaches.**	**Disjointed clinical services**.	**Limited or variable insurance coverage**.
If I want to get somebody screened for lung cancer, they may have to travel to a hospital 30 minutes away and not have even the gas money. If I want to get somebody for a biopsy and for specialty care, they're potentially looking at traveling an hour.' [provider (115)]	Sometimes patients will say, "well you know, God heals and God will heal me" or they'll say "I want to try an herbal natural remedy. [provider(113)]	Then with a diagnosis just sending her home with that order, I think was irresponsible. Somebody should have said, "Hey, do you know you have this on your lung", and nobody did.. [caregiver(111)]	Well, this is really being driven by the insurance company. Your doctor really making some of these decisions. The insurance company made it for them. And that doesn't fit with your "medical condition" at the moment [caregiver (110)].
**Avoiding medical care due to a previous encounter involving racial discrimination**.	**The patient's surroundings normalize tolerating symptoms.**	**Delays in obtaining appointments**.	**Policies and laws determining investment in healthcare**.

For peer review only - http://bmjopen.bmj.com/site/about/guidelines.xhtml

People have shared that they felt their expression of their pain level wasn't taken as seriously, because there might be some unconscious biases on the part of the healthcare provider about what they think about Black people and their threshold for pain tolerance. '[109]	I think sometimes people don't think things are serious because it's all perception. So, if you have a family member, who let's say, everybody in your household smokes and everybody complains of shortness of breath, you probably think that it's normal to be short of breath. [113]	Just figuring that out the next step of scheduling an appointment having to get on the phone and wait to schedule an appointment. It's not as easy as getting on to your computer sometimes, depending on the Health Care system.' [113]	It relates to what resources are available in your area. If you live in a rural community and you're the only doctor in that community and the nearest specialist is 2 hours away that can have an effect on your referral process [113]
**Cultural identity**.	**Hcps indulging in discriminatory practices**.	**Organizational demands on hcps to see more patients.**	**Limited management using telehealth**.
Sometimes it is just simply cultural beliefs. They don't want to admit that they have a problem. I mean, that could be something as simple as they have a pain somewhere. You know, i'll just take Tylenol or I'm coughing I won't go to the doctor, or they'll say things like you know. [provider (113)]	So, at the doctor level and, across cultures, they see an African-American person and they may or may not be obese, but there's so much emphasis on "You need to lose weight. You need to exercise. You need to eat right." And again, might be not investigating the extent by which the drinking and the smoking. [caregiver (119)]	If you have a new patient and someone telling you have to see patients every 15 minutes and you're at the end of the day or the middle of day and you're just trying to get through it. The provider can feel rushed and glazed over the patient's symptoms' [provider (113)]	He ended up going to get some antibiotics via telemedicine from a doctor. But I think I noticed more more persistent coughing. [caregiver (102)]
**Lack of health literacy**.	**Patients anticipating prejudice and discrimination**.		**Practitioner's inexperience**.
If there's no understanding of medicine, medical illness would be deemed as something annoying, like a cough. Not understanding risk factors for disease. Based on either smoking or family history or other people with those symptoms, not understanding when to seek care. [provider (114)]	It was awful. If he had taken the opportunity to send me for an x-ray and I had great insurance. It's not like I was like, hey, let's penny-pinch. I do not want to do this, or I do not want to do that." I followed all of the instructions that he had given me, down to the tee and it was never an option for him to send me for an x-ray. [patients (106)]		I truly believe that if you recognize something you order it in a timely manner, but if you don't recognize it, I think part of it is recognition of the problem if you don't recognize it, it can delay you'. [provider (113)]
**Not knowing which HCP to contact**.			**Complex health systems not designed for vulnerable patients**.
Finding a doctor is so messed up. You get no information on a doctor. You are given a list of names and you are supposed to pick one. Well, how am I supposed to pick them based on the name? That is the first barrier. [researcher (103)]			You have been in a hospital before. It is almost impossible to navigate a hospital because parking is difficult. Getting in the maze. Knowing where to go and where you cannot go is difficult. [researcher (103)]

For peer review only - http://bmjopen.bmj.com/site/about/guidelines.xhtml

DISCUSSION

This study is one of the first to report on the suboptimal experiences of BAA patients along the lung cancer care pathway. These experiences were identified by BAA patients, caregivers, and HCPs. The study found that some patients delayed seeking care, while others had their concerns ignored or were not offered molecular testing despite qualifying for it. Factors contributing to these suboptimal experiences included difficulties with insurance coverage, provider unwillingness to conduct comprehensive testing, provider bias in recommending treatment, high healthcare costs, and a lack of healthcare facilities and qualified staff to provide necessary tests and support. The study captured firsthand the individual and caregiver experiences, which were corroborated by community advocates and some healthcare provider experiences, particularly those with the lived experience of being BAAs.

Our findings indicate that at least some of the patients in the study, all BAA, experienced delays in both the diagnosis and initiation of treatment. Some patients received treatment for other common diseases before tests revealed their lung cancer. These delays are consistent with the 2021 State of Lung Cancer (SOLC) report, which found that BAAs patients were 16% less likely to receive an early diagnosis and 7% more likely to go untreated compared to their White American counterparts.(34) HCPs' actions in offering diagnostic testing play a critical role in patient experiences. Positive relationships with HCPs can motivate patients to seek care, but we found that delays were sometimes caused by patients' concerns about discrimination from providers who may not refer them for timely diagnosis and targeted treatment. The literature shows that BAA patients often perceive communication with physicians as less supportive, less collaborative, and less informative.(35) Our findings on communication and information sharing align with this, as we observed instances of critical information being withheld, findings being communicated in ways that were difficult for patients to understand, and information on available treatment options not being shared.

The factors we identified as contributing to delays in lung cancer diagnosis may also lead to poorer outcomes throughout the cancer care process.(36) BAA patients presenting at later stages of illness may experience delays for several reasons, such as limited health literacy and self-assessment of symptoms. Previous experiences with discrimination may also impact patients' decisions about seeking care. Additionally, patients have reported feeling that their providers did not consider their symptoms to be serious, resulting in the treatment of other possible infections instead of additional testing.(37, 38) On the other hand, patients in our study reported using various approaches to overcome the barriers they faced in their healthcare journey, including utilizing their knowledge of health systems, seeking information on diagnostic and treatment options, and engaging with their insurance companies and HCPs to access comprehensive tests and treatment options. This underscores the importance of patient empowerment in advocating for their rights with insurance companies and HCPs, as well as the significance of receiving sufficient information from healthcare providers to make informed decisions about their healthcare. Patient navigators may also assist patients and families in navigating the health system and overcoming socioeconomic challenges. Addressing racism and prejudices through multilevel strategies, including retraining doctors, engaging policymakers, and educating nonclinical staff on prejudice, systemic biases, and racism, is crucial.

For peer review only - http://bmjopen.bmj.com/site/about/guidelines.xhtml

Future research should aim to collect experiences of disparity from a more representative population and develop community-led interventions targeting individual and systemic factors perpetuating disparity. Evidence-based research methods should also focus on collecting patient perspectives relevant to experiences of racism, prejudice, and perceived injustices.

CONCLUSION

This study revealed suboptimal experiences and factors contributing to the disparities in lung cancer care among BAAs. Delays in diagnosis and initiation of treatment were reported, and patients faced various barriers, including insurance coverage, provider bias, and lack of healthcare facilities. BAA patients' empowerment and engagement with HCPs and insurance companies were important in navigating these barriers. Multilevel strategies are needed to address racism and prejudices in the healthcare system. Future research should focus on developing culturally-tailored community-centered interventions and evidence-based initiatives to collect, analyze, and disseminate patient perspectives on experiences of racism and perceived injustices. Although there were limitations to the study, the triangulation of perspectives and the use of robust frameworks strengthened the findings.

For peer review only - http://bmjopen.bmj.com/site/about/guidelines.xhtml

CONTRIBUTORSHIP STATEMENT

The listed authors, Nicholas Thuo (NT), Tanimola Martins (TM), Eugene Manley, Jr. (EMJ), Maisha Standifer (MS), Dawood H. Sultan (DHS), Nick Faris (NF) Angel Hill (AH), Matthew Thompson (MT), Rohan D. Jeremiah (RDJ), and Morhaf Al Achkar (MA) contributed to the conception, design, acquisition of data, or analysis and interpretation of data, drafting the article or critically revising it for important intellectual content, and final approval of the published version. NT and MA conceptualized this article and wrote the first draft along. NT, MA, TM, EMJ, NF, RDJ, MS, DHS, AH, and MT contributed to the revision and additional changes for the article to accurately reflect the research findings. MA affirms that the manuscript is an accurate representation of the issues covered, that there are no significant omissions or discrepancies. All authors have full responsibility for the work and decision to publish it.

COMPETING INTERESTS

All authors report no competing interests.

SOURCE OF FUNDING:

Funding: This research is linked to the CanTest Collaborative, which is funded by Cancer Research UK [C8640/A23385]. The project was also partially funded by a gift from LUNGevity Foundation.

DATA SHARING STATEMENT

The authors confirm that the data supporting the findings of this study are available within the article and its supplementary materials. The data will become available immediately after publication, with no end date for anyone who wishes access.

ETHICAL APPROVAL

Ethics approval was obtained from the University of Washington (UW) Human Subjects Division Reference number STUDY00005438.

For peer review only

BIBLIOGRAPHY

1. Ryan BM. Lung cancer health disparities. Carcinogenesis. 2018;39(6):741-51.
2. Soneji S, Tanner NT, Silvestri GA, Lathan CS, Black W. Racial and ethnic disparities in early-stage lung cancer survival. Chest. 2017;152(3):587-97.
3. Robbins HA, Engels EA, Pfeiffer RM, Shiels MS. Age at cancer diagnosis for blacks compared with whites in the United States. Journal of the National Cancer Institute. 2015;107(3):dju489.
4. Xu G, Cui P, Zhang C, Lin F, Xu Y, Guo X, et al. Racial disparities in bone metastasis patterns and targeted screening and treatment strategies in newly diagnosed lung cancer patients. Ethnicity & health. 2022;27(2):329-42.
5. American CancerSociety . Cancer Facts & Figures for African Americans 2022-2024. 2022.
6. Lathan CS, Neville BA, Earle CC. The effect of race on invasive staging and surgery in non–small-cell lung cancer. Journal of Clinical Oncology. 2006;24(3):413-8.
7. Hardy D, Liu CC, Xia R, Cormier JN, Chan W, White A, et al. Racial disparities and treatment trends in a large cohort of elderly black and white patients with nonsmall cell lung cancer. Cancer. 2009;115(10):2199-211.
8. Onega T, Duell EJ, Shi X, Demidenko E, Goodman DC. Race versus place of service in mortality among medicare beneficiaries with cancer. Cancer: Interdisciplinary International Journal of the American Cancer Society. 2010;116(11):2698-706.
9. Alvidrez J, Castille D, Laude-Sharp M, Rosario A, Tabor D. The national institute on minority health and health disparities research framework. American Journal of Public Health. 2019;109(S1):S16-S20.
10. Dempsey DA, Meyers MJ, Oh SS, Nguyen EA, Fuentes-Afflick E, Wu AH, et al. Determination of tobacco smoke exposure by plasma cotinine levels in infants and children attending urban public hospital clinics. Archives of pediatrics & adolescent medicine. 2012;166(9):851-6.
11. Haiman CA, Stram DO, Wilkens LR, Pike MC, Kolonel LN, Henderson BE, et al. Ethnic and racial differences in the smoking-related risk of lung cancer. New England Journal of Medicine. 2006;354(4):333-42.
12. Ho JT, Tyndale RF, Baker TB, Amos CI, Chiu A, Smock N, et al. Racial disparities in intensity of smoke exposure and nicotine intake among low-dependence smokers. Drug and Alcohol Dependence. 2021;221:108641.
13. Samet JM, Avila-Tang E, Boffetta P, Hannan LM, Olivo-Marston S, Thun MJ, et al. Lung cancer in never smokers: clinical epidemiology and environmental risk factors. Clinical Cancer Research. 2009;15(18):5626-45.
14. Jonnalagadda S, Lin JJ, Nelson JE, Powell CA, Salazar-Schicchi J, Berman AR, et al. Racial and ethnic differences in beliefs about lung cancer care. Chest. 2012;142(5):1251-8.
15. Williams CD, Salama JK, Moghanaki D, Karas TZ, Kelley MJ. Impact of race on treatment and survival among US veterans with early-stage lung cancer. Journal of Thoracic Oncology. 2016;11(10):1672-81.
16. Hendryx M, O'Donnell K, Horn K. Lung cancer mortality is elevated in coal-mining areas of Appalachia. Lung Cancer. 2008;62(1):1-7.
17. Zheng L, Enewold L, Zahm SH, Shriver CD, Zhou J, Marrogi A, et al. Lung cancer survival among black and white patients in an equal access health system. Cancer Epidemiology and Prevention Biomarkers. 2012;21(10):1841-7.

For peer review only - http://bmjopen.bmj.com/site/about/guidelines.xhtml

18. Brzezniak C, Satram-Hoang S, Goertz H-P, Reyes C, Gunuganti A, Gallagher C, et al. Survival and racial differences of non-small cell lung cancer in the United States Military. Journal of general internal medicine. 2015;30(10):1406-12.
19. Palazzo LL, Sheehan DF, Tramontano AC, Kong CY. Disparities and trends in genetic testing and erlotinib treatment among metastatic non–small cell lung cancer patients. Cancer Epidemiology and Prevention Biomarkers. 2019;28(5):926-34.
20. Li T, Kung H-J, Mack PC, Gandara DR. Genotyping and genomic profiling of non–small-cell lung cancer: implications for current and future therapies. Journal of Clinical Oncology. 2013;31(8):1039.
21. Kehl KL, Lathan CS, Johnson BE, Schrag D. Race, poverty, and initial implementation of precision medicine for lung cancer. JNCI: Journal of the National Cancer Institute. 2019;111(4):431-4.
22. Cote ML, Haddad R, Edwards DJ, Atikukke G, Gadgeel S, Soubani AO, et al. Frequency and type of epidermal growth factor receptor mutations in African Americans with non-small cell lung cancer. Journal of Thoracic Oncology. 2011;6(3):627-30.
23. Palazzo LL, Sheehan DF, Tramontano AC, Kong CY. Disparities and Trends in Genetic Testing and Erlotinib Treatment among Metastatic Non–Small Cell Lung Cancer PatientsGenetic Testing and Erlotinib Treatment. Cancer Epidemiology, Biomarkers & Prevention. 2019;28(5):926-34.
24. Lynch JA, Berse B, Rabb M, Mosquin P, Chew R, West SL, et al. Underutilization and disparities in access to EGFR testing among Medicare patients with lung cancer from 2010–2013. BMC cancer. 2018;18(1):1-13.
25. Bruno DS, Hess LM, Li X, Su EW, Zhu YE, Patel M. Racial disparities in biomarker testing and clinical trial enrollment in non-small cell lung cancer (NSCLC). Wolters Kluwer Health; 2021.
26. Harrison S, Judd J, Chin S, Ragin C. Disparities in lung cancer treatment. Current oncology reports. 2022;24(2):241-8.
27. Campbell JD, Lathan C, Sholl L, Ducar M, Vega M, Sunkavalli A, et al. Comparison of prevalence and types of mutations in lung cancers among black and white populations. JAMA oncology. 2017;3(6):801-9.
28. Evans III N, Grenda T, Alvarez NH, Okusanya OT. Narrative review of socioeconomic and racial disparities in the treatment of early stage lung cancer. Journal of Thoracic Disease. 2021;13(6):3758.
29. Morris AM, Rhoads KF, Stain SC, Birkmeyer JD. Understanding racial disparities in cancer treatment and outcomes. Journal of the American College of Surgeons. 2010;211(1):105-13.
30. Wisnivesky JP, McGinn T, Henschke C, Hebert P, Iannuzzi MC, Halm EA. Ethnic disparities in the treatment of stage I non–small cell lung cancer. American journal of respiratory and critical care medicine. 2005;171(10):1158-63.
31. Alhazmi AA, Kaufmann A. Phenomenological qualitative methods applied to the analysis of cross-cultural experience in novel educational social contexts. Frontiers in Psychology. 2022;13:785134.
32. Walter F, Webster A, Scott S, Emery J. The Andersen Model of Total Patient Delay: a systematic review of its application in cancer diagnosis. Journal of health services research & policy. 2012;17(2):110-8.

For peer review only - http://bmjopen.bmj.com/site/about/guidelines.xhtml

33. Al Achkar M, Suchsland MZ, Walter FM, Neal RD, Goulart BH, Thompson MJ. Experiences along the diagnostic pathway for patients with advanced lung cancer in the USA: a qualitative study. BMJ open. 2021;11(4):e045056.
34. American Lung Cancer Association. State of lung cancer 2021. 2021.
35. Gordon HS, Street Jr RL, Sharf BF, Kelly PA, Souchek J. Racial differences in trust and lung cancer patients' perceptions of physician communication. Journal of clinical oncology. 2006;24(6):904-9.
36. Zuniga PVS, Ost DE. Impact of delays in lung cancer treatment on survival. Chest. 2021;160(5):1934-58.
37. Lubuzo B, Ginindza T, Hlongwana K. Exploring barriers to lung cancer patient access, diagnosis, referral and treatment in Kwazulu-Natal, South Africa: The health providers' perspectives. Translational Lung Cancer Research. 2019;8(4):380.
38. Carter-Harris L, Hermann CP, Draucker CB. Pathways to a lung cancer diagnosis. Journal of the American Association of Nurse Practitioners. 2015;27(10):576-83.

For peer review only

For peer review only - http://bmjopen.bmj.com/site/about/guidelines.xhtml

Figure1. Suboptimal experiences throughout the diagnosis pathway.

For peer review only

For peer review only - http://bmjopen.bmj.com/site/about/guidelines.xhtml

Figure1. suboptimal experiences throughout the diagnosis pathway.

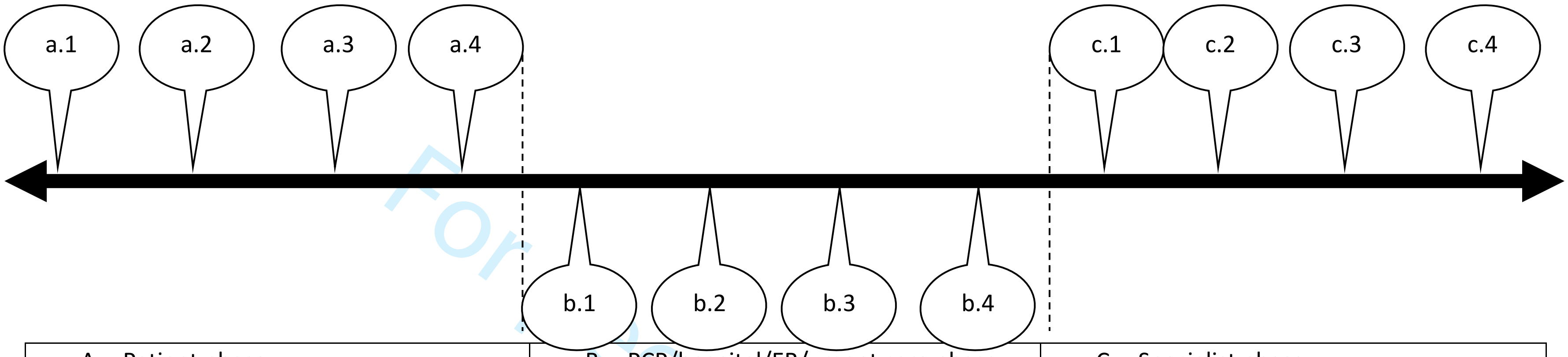

A. Patient phase	B. PCP/hospital/ER/urgent care phase	C. Specialist phase
a.1. Not being alarmed by the symptoms as they appeared	b.1. Being treated for common, more benign conditions	c.1. Difficulty finding an appointment to see the specialist
a.2. Explaining symptoms as caused by other conditions	b.2. Not offered diagnostic imaging	c.2. Poor delivery of information by the provider
a.3. Prolonging self-managing of symptoms	b.3. Not being informed of abnormal findings on tests	c.3. Not offered indicated molecular testing
a.4. Seeking care only as symptoms became severe	b.4. Poor communication by providers leaving the patient with uncertainty	c.4. Steering patients to receive unnecessary chemotherapy

For peer review only

Appendix 1. Interview guides.

For peer review only

Semi-Structured Interview Guide

The study has two aims: Aim 1: Explore pitfalls, suboptimal experiences, and discriminatory practices in cancer diagnosis pathways for African Americans with metastatic lung cancer and oncogenic alterations. Aim 2: Identify etiologies of health disparity in cancer diagnosis for African Americans with metastatic lung cancer.

Cancer diagnosis

a. Tell me about your cancer diagnosis story, starting from when you noticed your first symptoms.

b. Patient phase
   - i. What symptoms did you have that led to the cancer diagnosis?
   - ii. How did the symptoms change over time before diagnosis?
   - iii. Did anyone else notice your symptoms?
   - iv. What did you think was the cause of your symptoms?
   - v. How concerned were you about your symptoms and why?
   - vi. What prompted you to seek medical care the first time?
   - vii. Where did you seek care for the first time? Who else did you see?
   - viii. How long do you think your symptoms were present before you sought care?
   - ix. In your opinion, what contributed to that time (delay) between symptoms starting and seeking medical care?

c. PCP phase
   - i. Describe your first interaction with your healthcare providers when you presented with symptoms.
   - ii. What took place during the visit?
   - iii. How did the provider explain your symptoms?
   - iv. What were the steps taken to investigate your symptoms?
   - v. What was the follow-up plan?
   - vi. Were you offered any treatment for your symptoms? What was it?
   - vii. Were you referred anywhere else? To whom?
   - viii. How long after your doctor's visit did you get your first imaging test?
   - ix. How was the decision made to get an imaging test?
   - x. How were the findings on the imaging tests explained to you?
   - xi. Was there any other tests ordered? What are they?
   - xii. How much wait time did you have between your doctor's visit and getting the tests?
   - xiii. In your opinion, what contributed to that time (delay) between seeking medical care and getting imaging tests?

d. Specialty care phase
   - i. How was the decision made that you should see a specialist?
   - ii. Describe the interactions with the specialists (pulmonologist, surgeon, radiologist, etc.) that led to getting a biopsy to confirm the diagnosis.
   - iii. How were the results explained to you?
   - iv. What other tests or procedures did you have early on?
   - v. Share about your first interaction with the oncologist.
   - vi. How much wait time did you have between receiving the cancer diagnosis and seeing an oncologist?
   - vii. How was the *diagnosis* explained to you? What did they say about the disease you have?

For peer review only

viii. How was the *prognosis* explained to you? What did they say about what to expect will happen?
ix. What did they explain about the treatment options?
x. How was the decision made to get molecular testing?
xi. How were the tests explained to you?
xii. How were the results explained to you?
xiii. What treatment did you receive first?
xiv. How was the decision made to get a specific treatment?
xv. What other treatments have you received?
xvi. How much wait time did you have between your oncologist visit and starting treatment?

e. Before cancer
   i. How was your health before cancer?
   ii. Share about your lifestyle before cancer.
   iii. Have you smoked? If so, have you been offered smoking cessation?
   iv. How much did you smoke? If significant amount, have you been offered lung cancer screening?
   v. Have you had exposure to any carcinogen?
   vi. Share about encounters with healthcare before cancer.
   vii. What health issues did you deal with before cancer?
   viii. Did you have a PCP? Tell me about them? if not, why?

f. Reflections on etiologies of potential delays
   i. You mentioned, [example of possible inadequate/delay/suboptimal], from your vantage point, why do you think this happened?
   ii. What can be done to avoid such pitfalls from happening to other people who maybe in similar positions to yours?
   iii. (rephrase questions of focus group to ask patients/caregivers to identify factors and structures contributed to less than optimal outcomes for them or other people in their situation?)

Demographics

- What is your age?
  - Under 18
  - 18-24
  - 25-44
  - 45-64
  - Over 65
- What is your gender?
  - Male
  - Female
  - Other (please specify)
  - Prefer not to say
- What is your race/ethnicity?
  - White
  - Hispanic/Latino
  - Black/African American
  - Native American/American Indian
  - Asian/Pacific Islander
  - Other

For peer review only

- What is the highest level of school you've completed?
  - Less than a high school diploma
  - High school diploma or equivalent
  - Some college
  - Bachelor's degree
  - Master's degree
  - Doctorate
  - Other (please specify)
- What is your current employment status?
  - Employed Full-time (40 hours per week)
  - Employed Part-time (Less than 40 hours per week)
  - Unemployed
  - Student
  - Retired
  - Self-employed
  - Unable to work
- What is your home zip code?
- What is your marital status?
  - Single (Never married)
  - Married
  - Divorced
  - Widowed
- What is your household income?
  - Below $10K
  - $10K – $50K
  - $50K – $100K
  - $100K - $150K
  - Over $150K
- Cancer date of diagnosis:____
- Type of cancer:____
- Stage at time of diagnosis:____
- Stage now if not IV at time of diagnosis:____

For peer review only

Health Care Providers and Community Advocates

- We define cancer health disparity as the health difference, on the basis of one or more health outcomes, that adversely affects black/african americans. Specifically, We are interested in Identifying etiologies of health disparity around cancer diagnosis for African Americans with advanced lung cancer and oncogenic alterations.
- We want you help understanding the factors that cause this disparity. We are using the NIMHD model for health disparity. According to this framework, we examining the issue across 5 domains and 4 levels. See figure.

Domains of Influence (*Over the Lifecourse*)	Levels of Influence*			
	**Individual**	**Interpersonal**	**Community**	**Societal**
**Biological**	Biological Vulnerability and Mechanisms	Caregiver–Child Interaction<br>Family Microbiome	Community Illness Exposure<br>Herd Immunity	Sanitation<br>Immunization<br>Pathogen Exposure
**Behavioral**	Health Behaviors<br>Coping Strategies	Family Functioning<br>School/Work Functioning	Community Functioning	Policies and Laws
**Physical/Built Environment**	Personal Environment	Household Environment<br>School/Work Environment	Community Environment<br>Community Resources	Societal Structure
**Sociocultural Environment**	Sociodemographics<br>Limited English<br>Cultural Identity<br>Response to Discrimination	Social Networks<br>Family/Peer Norms<br>Interpersonal Discrimination	Community Norms<br>Local Structural Discrimination	Social Norms<br>Societal Structural Discrimination
**Health Care System**	Insurance Coverage<br>Health Literacy<br>Treatment Preferences	Patient–Clinician Relationship<br>Medical Decision-Making	Availability of Services<br>Safety Net Services	Quality of Care<br>Health Care Policies
**Health Outcomes**	Individual Health	Family/ Organizational Health	Community Health	Population Health

This is the cancer diagnosis pathway.

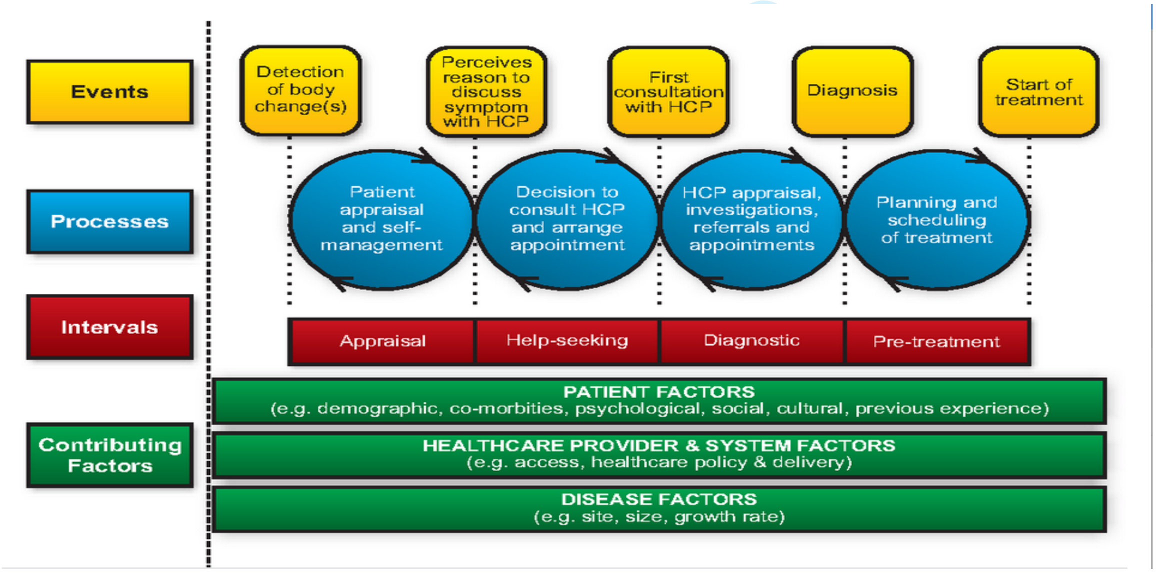

1. What are factors and structures that lead to delays in detecting first symptoms?

2. What are factors and structures that lead to inappropriate appraisal of symptoms?
3. What are factors and structures that lead to spending longer time in self-management phase as opposed to seeking help?
4. What are factors and structures that lead to not perceiving reasons to discuss symptoms with health care providers in a timely way?
5. What are factors and structures that lead to delays in consulting health care providers?
6. What are factors and structures that lead to not being able to arrange for appointments?
7. What are factors that lead to inadequate appraisal of symptoms by the health care provider on early visits?
8. What are factors and structures that lead to not ordering appropriate investigations in a timely manner?
9. What are factors and structures that lead to not arranging for referrals once suspicion of cancer become high (i.e., delays in getting to be seen by a specialist)?
10. What are factors and structures that lead to failing to obtain molecular testing
11. What are factors and structures that lead to not getting molecular testing?
12. What are factors and structures that lead to delays in getting molecular testing?
13. What are factors and structures that lead to delays in starting appropriate targeted therapy?

Caregiver's interviews are similar but rephrased to get the perspectives of the significant other on the health/experience of the patient.

Demographics

- What is your age?
  - Under 18
  - 18-24
  - 25-44
  - 45-64
  - Over 65
- What is your gender?
  - Male
  - Female
  - Other (please specify)
  - Prefer not to say
- What is your race/ethnicity?
  - White
  - Hispanic/Latino
  - Black/African American
  - Native American/American Indian
  - Asian/Pacific Islander
  - Other
- What is the highest level of school you've completed?
  - Less than a high school diploma
  - High school diploma or equivalent
  - Some college

For peer review only

- Bachelor's degree
- Master's degree
- Doctorate
- Other (please specify)

- What is your current employment status?
  - Employed Full-time (40 hours per week)
  - Employed Part-time (Less than 40 hours per week)
  - Unemployed
  - Student
  - Retired
  - Self-employed
  - Unable to work
- What is your home zip code?
- What is your marital status?
  - Single (Never married)
  - Married
  - Divorced
  - Widowed
- What is your household income?
  - Below $10K
  - $10K – $50K
  - $50K – $100K
  - $100K - $150K
  - Over $150K

For peer review only

Standards for Reporting Qualitative Research (SRQR)*

http://www.equator-network.org/reporting-guidelines/srqr/

Title and abstract

	Page/line no(s).
**Title** - Concise description of the nature and topic of the study Identifying the study as qualitative or indicating the approach (e.g., ethnography, grounded theory) or data collection methods (e.g., interview, focus group) is recommended	1
**Abstract** - Summary of key elements of the study using the abstract format of the intended publication; typically includes background, purpose, methods, results, and conclusions	3

Introduction

**Problem formulation** - Description and significance of the problem/phenomenon studied; review of relevant theory and empirical work; problem statement	5
**Purpose or research question** - Purpose of the study and specific objectives or questions	6

Methods

**Qualitative approach and research paradigm** - Qualitative approach (e.g., ethnography, grounded theory, case study, phenomenology, narrative research) and guiding theory if appropriate; identifying the research paradigm (e.g., postpositivist, constructivist/ interpretivist) is also recommended; rationale**	6
**Researcher characteristics and reflexivity** - Researchers' characteristics that may influence the research, including personal attributes, qualifications/experience, relationship with participants, assumptions, and/or presuppositions; potential or actual interaction between researchers' characteristics and the research questions, approach, methods, results, and/or transferability	7
**Context** - Setting/site and salient contextual factors; rationale**	6
**Sampling strategy** - How and why research participants, documents, or events were selected; criteria for deciding when no further sampling was necessary (e.g., sampling saturation); rationale**	6
**Ethical issues pertaining to human subjects** - Documentation of approval by an appropriate ethics review board and participant consent, or explanation for lack thereof; other confidentiality and data security issues	6
**Data collection methods** - Types of data collected; details of data collection procedures including (as appropriate) start and stop dates of data collection and analysis, iterative process, triangulation of sources/methods, and modification of procedures in response to evolving study findings; rationale**	6

For peer review only - http://bmjopen.bmj.com/site/about/guidelines.xhtml

**Data collection instruments and technologies** - Description of instruments (e.g., interview guides, questionnaires) and devices (e.g., audio recorders) used for data collection; if/how the instrument(s) changed over the course of the study	6
**Units of study** - Number and relevant characteristics of participants, documents, or events included in the study; level of participation (could be reported in results)	7
**Data processing** - Methods for processing data prior to and during analysis, including transcription, data entry, data management and security, verification of data integrity, data coding, and anonymization/de-identification of excerpts	7
**Data analysis** - Process by which inferences, themes, etc., were identified and developed, including the researchers involved in data analysis; usually references a specific paradigm or approach; rationale**	7
**Techniques to enhance trustworthiness** - Techniques to enhance trustworthiness and credibility of data analysis (e.g., member checking, audit trail, triangulation); rationale**	7

Results/findings

**Synthesis and interpretation** - Main findings (e.g., interpretations, inferences, and themes); might include development of a theory or model, or integration with prior research or theory	7-11
**Links to empirical data** - Evidence (e.g., quotes, field notes, text excerpts, photographs) to substantiate analytic findings	7 (tables)

Discussion

**Integration with prior work, implications, transferability, and contribution(s) to the field** - Short summary of main findings; explanation of how findings and conclusions connect to, support, elaborate on, or challenge conclusions of earlier scholarship; discussion of scope of application/generalizability; identification of unique contribution(s) to scholarship in a discipline or field	11-12
**Limitations** - Trustworthiness and limitations of findings	

Other

**Conflicts of interest** - Potential sources of influence or perceived influence on study conduct and conclusions; how these were managed	13
**Funding** - Sources of funding and other support; role of funders in data collection, interpretation, and reporting	13

*The authors created the SRQR by searching the literature to identify guidelines, reporting standards, and critical appraisal criteria for qualitative research; reviewing the reference lists of retrieved sources; and contacting experts to gain feedback. The SRQR aims to improve the transparency of all aspects of qualitative research by providing clear standards for reporting qualitative research.

For peer review only - http://bmjopen.bmj.com/site/about/guidelines.xhtml

**The rationale should briefly discuss the justification for choosing that theory, approach, method, or technique rather than other options available, the assumptions and limitations implicit in those choices, and how those choices influence study conclusions and transferability. As appropriate, the rationale for several items might be discussed together.

Reference:

O'Brien BC, Harris IB, Beckman TJ, Reed DA, Cook DA. **Standards for reporting qualitative research: a synthesis of recommendations.** *Academic Medicine*, Vol. 89, No. 9 / Sept 2014 DOI: 10.1097/ACM.0000000000000388

For peer review only

For peer review only - http://bmjopen.bmj.com/site/about/guidelines.xhtml

BMJ Open

Factors Leading To Disparity In Lung Cancer Diagnosis Among Black/ African American Communities in the United States: A Qualitative Study

Journal:	*BMJ Open*
Manuscript ID	bmjopen-2023-073886.R2
Article Type:	Original research
Date Submitted by the Author:	15-Sep-2023
Complete List of Authors:	Thuo , Nicholas; University of Washington Seattle Campus<br>Martins, Tanimola; University of Exeter, Primary Care; University of Exeter , Primary Care<br>Manley Jr. , Eugene ; LUNGevity Foundation<br>Standifer, Maisha ; Morehouse School of Medicine<br>Sultan , Dawood ; Mercer University<br>Faris , Nick ; Baptist Memorial Health Care Corp<br>Hill , Angela ; University of South Florida<br>Thompson, Matthew; University of Washington, Department of Family Medicine<br>Jeremiah, Rohan ; University of Illinois at Chicago,<br>Al Achkar, Morhaf; University of Washington, Family Medicine ;
**Primary Subject Heading**:	Oncology
Secondary Subject Heading:	Public health
Keywords:	Public health < INFECTIOUS DISEASES, Adult oncology < ONCOLOGY, Respiratory tract tumours < ONCOLOGY

SCHOLARONE™
Manuscripts

I, the Submitting Author has the right to grant and does grant on behalf of all authors of the Work (as defined in the below author licence), an exclusive licence and/or a non-exclusive licence for contributions from authors who are: i) UK Crown employees; ii) where BMJ has agreed a CC-BY licence shall apply, and/or iii) in accordance with the terms applicable for US Federal Government officers or employees acting as part of their official duties; on a worldwide, perpetual, irrevocable, royalty-free basis to BMJ Publishing Group Ltd ("BMJ") its licensees and where the relevant Journal is co-owned by BMJ to the co-owners of the Journal, to publish the Work in this journal and any other BMJ products and to exploit all rights, as set out in our licence.

The Submitting Author accepts and understands that any supply made under these terms is made by BMJ to the Submitting Author unless you are acting as an employee on behalf of your employer or a postgraduate student of an affiliated institution which is paying any applicable article publishing charge ("APC") for Open Access articles. Where the Submitting Author wishes to make the Work available on an Open Access basis (and intends to pay the relevant APC), the terms of reuse of such Open Access shall be governed by a Creative Commons licence – details of these licences and which Creative Commons licence will apply to this Work are set out in our licence referred to above.

Other than as permitted in any relevant BMJ Author's Self Archiving Policies, I confirm this Work has not been accepted for publication elsewhere, is not being considered for publication elsewhere and does not duplicate material already published. I confirm all authors consent to publication of this Work and authorise the granting of this licence.

For peer review only

Factors Leading To Disparity In Lung Cancer Diagnosis Among Black/ African American Communities in the United States: A Qualitative Study

Nicholas Thuo[1], Tanimola Martins[2], Eugene Manley, Jr.[3], Maisha Standifer[4], Dawood H. Sultan[5], Nick Faris[6], Angela Hill[7], Matthew Thompson [1], Rohan D. Jeremiah[8], Morhaf Al Achkar[1]

1. Department of Family Medicine, University of Washington, Seattle, WA, USA

2. University of Exeter Medical School, Exter, UK

3. LUNGevity Foundation, Chicago, IL, USA

4. Morehouse School of Medicine, Satcher Health Leadership Institute, Atlanta, GA, USA

5. Mercer University, Atlanta, Georgia, USA

6. Baptist Memorial Health System, Memphis, TN, USA

7. University of South Florida, Tampa, FL, USA

8. University of Illinois Chicago, College of Nursing, Chicago, IL, USA

Corresponding author

Morhaf Al Achkar

Department of Medicine, University of Washington, Seattle, WA;

Tel: +2068009480

Email: alachkar@uw.edu

Keywords: Lung cancer, disparity, sub-optimal experiences, NIMHD model for health disparity

For peer review only - http://bmjopen.bmj.com/site/about/guidelines.xhtml

Abstract word count: 298

Word Count: 3393

Tables: 3

Figures: 1

References: 38

For peer review only

For peer review only - http://bmjopen.bmj.com/site/about/guidelines.xhtml

ABSTRACT

Objective: This study has two objectives: first, to explore the diagnostic experiences of Black/African American (BAA) patients with lung cancer to pinpoint pitfalls, suboptimal experiences, and instances of discrimination leading to disparities in outcomes compared to patients of other ethnic backgrounds, especially white patients. The second objective is to identify the underlying causes contributing to health disparities in the diagnosis of lung cancer among BAA patients.

Methods: We employed a phenomenological research approach, guiding in-depth interviews with patients self-identifying as BAA diagnosed with lung cancer, as well as caregivers, healthcare professionals, and community advocates knowledgeable about BAA experiences with lung cancer. We performed thematic analysis to identify experiences at patient, primary care and specialist levels. Contributing factors were identified using the National Institute of Minority Health and Health Disparities (NIHMD) health disparity model.

Results: From March to November 2021, we conducted individual interviews with 19 participants, including 9 patients/caregivers and 10 providers/advocates. Participants reported recurring and increased pain before seeking treatment, treatment for non-cancer illnesses, delays in diagnostic tests and referrals, poor communication and bias when dealing with specialists and primary care providers. Factors contributing to suboptimal experiences included reluctance by insurers to cover costs, provider unwillingness to conduct comprehensive testing, provider bias in recommending treatment, high healthcare costs, and lack of healthcare facilities and qualified staff to provide necessary support. However, some participants reported positive experiences due to their insurance, availability of services, and having an empowered support structure.

Conclusions: BAA patients and caregivers encountered suboptimal experiences during their care. The NIMHD model is a useful framework to organize factors contributing to these experiences that may be leading to health disparities. Additional research is needed to fully capture the extent of these experiences and identify ways to improve BAA patient experiences in the lung cancer diagnosis pathway.

For peer review only

STRENGTHS AND LIMITATIONS OF THIS STUDY

- Strengths of this study included leveraging the perspectives of lung cancer survivors, caregivers, and frontline healthcare providers to provide diverse, real-life insights.
- Robust frameworks, including the cancer diagnosis pathway and NIMHD research framework, were used to organize the findings.
- The voices of BAA patients were prioritized to provide transparent, authentic perspectives.
- Limitations included a highly educated, insured population and a small sample size, potentially missing the views of diverse and underserved patients.

KEY MESSAGES

What is already known on this topic:

Black/African Americans (BAAs) have the highest morbidity and mortality rates for most cancers compared to any other racial group. Addressing the root causes of sub-optimal experiences for BAAs in cancer diagnosis pathways can help mitigate such disparities.

What this study adds:

Our study delves into the experiences of lung cancer patients, caregivers, and healthcare providers along the diagnostic and treatment pathways, highlighting the factors contributing to sub-optimal experiences.

How this study might affect research, practice, or policy:

A deeper understanding of these experiences and the factors influencing them could lead to positive changes in provider-patient interactions, identify areas for improvement in provider training and health systems, and improve cancer diagnosis and treatment pathways for BAAs.

For peer review only - http://bmjopen.bmj.com/site/about/guidelines.xhtml

INTRODUCTION

Lung cancer disproportionately affects Black/African American (BAA) communities in the United States (US).(1-5) In 2022, BAA had a lower estimated 5-year survival rate (20%) compared to Whites (22%).(5) BAA in the US have a lower rate of early-stage detection for lung cancer, at only 16% (compared to 20% among all races), with 53% being diagnosed at distant locations (compared to 49% for non-Hispanic Whites). (5) BAAs are less likely than patients from other racial/ethnic groups to be staged during diagnosis, and to receive curative treatment at the most optimal point in the course of the disease. (2, 6, 7) These disparities appear to be unrelated to health insurance, as BAA Medicare beneficiaries with lung cancer experience higher mortality rates than non-Hispanic Whites.(8)

The NIMHD research framework offers guidance on considering the domains and levels of influence of factors that may account for why lung cancer disproportionately affects BAAs.(9) Differences in nicotine metabolism, which are linked to genetics and heredity, have been identified as a potential biological vulnerability.(1, 10, 11). Documented behavioral factors include smoking patterns, exposure to secondhand smoke, and care avoidance due to beliefs and fears.(1, 12) Environmental factors, including greater exposure to radon and indoor pollutants, living near smelting factories, and working in coal mines, have been identified as potential contributing factors to these disparities.(13) Lower educational attainment, rurality, poverty, and limited access to healthcare due to employment are significant implicated sociocultural factors.(14-16) Research further indicates that systemic racism leading to inequitable access to quality healthcare is the critical factor for the gap in outcomes when a BAA develops lung cancer.(17, 18)

Routine use of molecular testing and targeted cancer therapy for late-stage non-small cell lung cancer (NSCLC) has significantly reduced mortality rates.(19, 20) Patients who receive molecular testing within 60 days of diagnosis have better survival rates compared to those who do not.(21) Studies have found that BAAs have similar rates of oncogenic alterations as non-Hispanic Whites and at least one in every three BAA patients have an actionable mutation.(22) Nevertheless, BAAs tend to receive molecular testing or targeted treatment less frequently than Whites.(21) Even in Medicare populations, compared to non-Hispanic Whites, BAA patients over 65 years old with NSCLC had lower odds ratios of 0.53-0.63 for receiving targeted therapy.(23, 24) White patients with NSCLC were more likely to receive next-generation sequencing, compared to BAA patients (50.1% vs 39.8%, $p<0.0001$).(25) Limited access to testing among BAA patients may be contributing to disparities in outcomes.(26) Studies have shown that when given access to care, BAA lung cancer patients have the same outcomes as White patients.(22, 27)

Extensive literature exists on the roots of disparities and how to eliminate them for patients with early-stage lung cancers.(2, 28-30) However, little is known about the etiologies of disparity for patients with late-stage lung cancer. While it is possible that etiologies of disparities in treating metastatic diseases share some commonalities with early-stage cancers, they may also have unique attributes contributing to the complex and long-term treatment paradigm of metastatic

For peer review only - http://bmjopen.bmj.com/site/about/guidelines.xhtml

diseases. This study aimed to achieve two objectives: the first was to explore the diagnostic pathways for BAAs with lung cancer to identify pitfalls, suboptimal experiences, and discriminatory practices occurred. The second objective was to identify the etiologies of health disparity in cancer diagnosis for BAAs with lung cancer.

METHODS

Study Setting, Population, and Design

We utilized a phenomenological research design to guide qualitative interviews conducted with patients, caregivers, healthcare professionals, and community advocates.(31) Patient and caregiver interviews provided patient-level accounts to explore experiences and identify diagnostic pathways and challenges encountered, from which we identified suboptimal experiences during the diagnostic phase. Healthcare professional and community advocate interviews produced reflections and observations to contextualize possible causes of observed challenges for individuals in BAA communities. The study was co-designed and conducted with the help of a community advisory group (CAG) called Project RADICAL - Racial DIsparities in CAncer of the Lung - formed in 2020. The CAG, consisting of patients, caregivers, lung cancer researchers, and community advocates, met biweekly to develop the study protocol, inform study methods and interview guides, and interpret findings. The University of Washington's Institutional Review Board approved the study.

Patient and Public Involvement

We used a purposive sampling strategy to identify participants who met the inclusion criteria, which included patients aged 18 years or older who self-identified as BAA and had been diagnosed with metastatic lung cancer in the United States. Caregivers were individuals providing primary care for a BAA lung cancer patient. Healthcare providers (HCPs) and community advocates were practicing physicians providing oncology or primary care services, or advocates for BAA communities. To identify participants, we used snowball sampling methods through the community network of Project RADICAL CAG members, collaborators' networks in cancer organizations, online support groups such as ALK-positive, and academic list servers focused on disparities.

Data Collection

The research team developed the interview guides using the Anderson's model of pathways to cancer treatment.(32) Based on the findings of our previous work(31), the model was refined to organize patients' diagnostic experiences into three phases: 1) patient phase (from first symptom recognition to the first doctor's visit in primary care), 2) primary care phase (from the first primary care visit to presumptive diagnosis based on imaging), and 3) secondary care phase (from referrals to biopsy until identifying actionable genes and starting treatment).(33) The patient and caregiver interviews began with open-ended questions about their experiences with a cancer diagnosis. HCP and community advocate interviews aimed to identify factors contributing to suboptimal experiences along the diagnosis pathway (see Appendix 1 for interview guides). At the end of the interviews, participants shared their demographics, including the race they identify

For peer review only - http://bmjopen.bmj.com/site/about/guidelines.xhtml

with. An experienced qualitative researcher (MA) conducted all interviews via phone or video conference, with most conducted via Zoom. Interviews were audio recorded and transcribed verbatim. Participants received a $50 gift card for participating in the 30-60 minute interviews.

Analysis

An experienced qualitative researcher (NT) coded the interviews using Dedoose (Dedoose Inc., Manhattan Beach, California) for analysis. The senior author (MA) provided peer debriefing and supervised NT's coding. Transcripts were analysed using inductive and deductive strategies, with codes developed highlighting suboptimal experiences and factors leading to disparity. The NIMHD research framework was used to organize the emerging factors contributing to disparity, and patient and caregiver narratives were triangulated with community advocates and HCPs' reflections. Findings were presented to the CAG and co-authors, and inputs were used to iteratively develop themes.

Researchers' Characteristics and Reflexivity

The research team brings diverse backgrounds, experiences, and perspectives to this qualitative study. MA is a male, PhD-trained qualitative researcher, identifying as an Arab immigrant, with personal experience living with advanced lung cancer. MS is a female, PhD-trained qualitative researcher, identifying as BAA, and has served as a caregiver for a BAA patient who succumbed to advanced lung cancer. NT, an MPH-trained and experienced qualitative researcher, identifies as Black African from Kenya. TM is a male, PhD-trained qualitative researcher, identifying as Black African from Nigeria. RDJ is a male, PhD-trained researcher with expertise in qualitative research, identifying as BAA. DHS is a male, PhD-trained health service researcher, identifying as BAA. NF holds a Master of Divinity and serves as a research manager and health equity leader; he identifies as a White male. AH is a female, Pharm.D with experience in disparity research, identifying as BAA, and has had a family member who died of advanced lung cancer. EMJ is a male, PhD-trained molecular and cell biologist and researcher, identifying as BAA, and has studied lung cancer therapeutics and is a patient advocate. MT is a male, PhD-trained family doctor and researcher, who has also experienced the loss of a family member to lung cancer.

RESULTS

The demographic characteristics of the participants are presented in Table 1. Participants were aged 25–64 years old, with 55% female,78% self-identified as BAA, and 58% were patients or caregivers. At the time of the interview, 94% were in full-time employment. We will describe the themes that emerged in two main areas, namely suboptimal experiences and factors leading to disparities.

For peer review only - http://bmjopen.bmj.com/site/about/guidelines.xhtml

Table 1. Participants' demographics.

ID	Age Group	Gender	Race /Ethnicity	Education	Zipcode	Marital status	income (000 $)	Role
101	25-44	Female	BAA	Doctorate	11203	Single	<50	Patient
102	45-64	Female	BAA	some college	33705	Married	50-100	Caregiver
103	45-64	Male	BAA	Doctorate	46268	Married	100-150	Researcher
104	>65	Female	BAA	Doctorate	60621	Divorced	50-100	Patient*
105	>65	Female	BAA	Masters	60652	Married	50-100	Caregiver
106	25-44	Female	BAA	Bachelors	10701	Single	100-150	Patient
107	45-64	Female	BAA	Bachelors	46234	Married	100-150	Patient
108	25-44	Female	BAA	Bachelors	46268	Married	50-100	Advocate
109	45-64	Male	BAA	some college	91367	Single	50-100	Patient
110	45-64	Male	BAA	Masters	46278	Married	100-150	caregiver
111	45-64	Female	BAA	Doctorate	46214	Married	>150	caregiver
112	Unknown	Male	BAA	Doctorate	Unknown	Married	>150	oncologist
113	25-44	Female	BAA	Doctorate	31008	Married	>150	Provider
114	45-64	Female	BAA	Doctorate	15146	Married	>150	Provider
115	45-64	Male	White	Doctorate	43452	Married	>150	Provider
116	45-64	Male	White	Doctorate	80111	Married	>150	Provider*
117	45-64	Male	White	Doctorate	98115	Married	>150	provider
118	45-64	Female	White	Master's	41042	Single	$50k-$100k	Nurse Navigator
119	45-64	Female	BAA	Doctorate	33579	Married	>150	caregiver

* Indicates participants who were retired, all other participants were in full-time employment

For peer review only - http://bmjopen.bmj.com/site/about/guidelines.xhtml

Suboptimal experiences along the diagnosis pathways

We organized the suboptimal experiences within the patient phase, the PCP/hospital/ER/urgent care phase, and the specialist phase. Figure 1 depicts suboptimal experiences throughout the diagnosis pathway. Table 2 includes supportive quotes.

Patient Phase

- **Not being alarmed by the symptoms as they appeared.** Some patients did not seek care right away as they were not concerned about the symptoms either because of their mild nature or the failure to appraise their seriousness.
- **Explaining symptoms as caused by other conditions.** Patients and caregivers at times attributed symptoms to less serious health conditions, such as common cold and allergies, delaying seeking medication consultation.
- **Prolonging self-managing of symptoms.** Patients resorted to home remedies or over-the-counter treatment for cancer-related symptoms (e.g., cough and pain) instead of seeking medical care.
- **Seeking care only as symptoms became severe.** The threshold to seek medical advice was not crossed until symptoms were too serious to ignore or especially bothersome (e.g., severe pain).

PCP/Hospital/ER/Urgent Care Phase

- **Being treated for common, more benign conditions.** Patients frequently received treatment for more common and less serious conditions, such as sinus infections or acid reflux. Patients then ignored the symptoms without clear instructions or a follow-up plan.
- **Delayed or not being offered diagnostic imaging.** Some patients were not offered diagnostic tests due to the HCP's low levels of suspicion for lung cancer.
- **Not being informed of abnormal findings on tests.** Some patients were not informed about abnormal findings on their diagnostic tests and were left without any follow-up. On occasions, patients were not informed about the findings or the plan and they discovered them by reading the handouts.
- **Communicated information poorly and left with uncertainty.** Patients were kept uninformed regarding the plans of care. Sometimes they were not told how they could receive help if they needed urgent care or had questions.

Secondary Care Phase

- **Difficulty finding an appointment to see the specialist.** Some patients, especially in underserved areas, had difficulty finding timely appointments to see specialists.
- **Poor delivery of information by the HCP.** Some patients were hassled during their care, receiving sub-par communication or little guidance to make decisions.
- **Not receiving molecular testing.** At times, patients were not offered molecular testing—a standard of care—and as a result, missed an opportunity to receive novel treatments.

For peer review only - http://bmjopen.bmj.com/site/about/guidelines.xhtml

- **Feeling steered to receive unnecessary chemotherapy.** Some patients felt they were pushed to take the only option of chemotherapy, while other more-novel treatment options were not discussed.

For peer review only

For peer review only - http://bmjopen.bmj.com/site/about/guidelines.xhtml

Table 2. Supportive Quotes for The Suboptimal Experiences Along The Diagnosis Pathways

Patient phase	PCP/hospital/ER/urgent care phase	Specialist phase
**Not being alarmed by the symptoms as they appeared.**	**Being treated for common, more benign conditions.**	**Difficulty finding appointment to see the specialist.**
Time went on and cough never got better, but it did not seem to get worse. [patient (107)]	They'll say, "Hey, it's sinus or allergies," they'll give them an antibiotic. [Nurse navigator (118)]	If you live in a rural community and you're the only doctor in that community and the nearest specialist is 2 hours or 45 minutes away that can have an effect on your referral process and your ability to refer at times. [Provider (113)]
	My primary care doctor diagnosed me with having allergies as well as acid reflux. I was treated with Protonix. I was treated with Claritin, over-the-counter stuff and eventually I would just ignore it. [patient (107)]	
**Explaining symptoms as caused by other conditions.**	**Delayed or not being offered diagnostic imaging.**	**Poor delivery of information by the provider.**
They had similar symptoms before and they resolved on their own without seeking help or with conservative measures; may be that if they have ignored in the past that can lead to worst consequence for them. [Provider (106)]	I went to another doctor and the first thing the doctor asked me "had you had an x-ray?" I was like, "No. Nobody sent me for an x-ray." [Patient (106)]	It was just another bad experience where I was sitting in the doctors for my appointment. He was perpetually on the telephone talking to other people as I was sitting there and then kind of squeezing in like when he was on hold or between calls telling me something really quick. [Patient (109)]
She had a cough for quite a while and we thought that it was allergies. Then, unfortunately, she was in a head-on collision with a drunk driver and it broke the transverse process of her C7 vertebrae. [Caregiver (104)]	You can't do chest CTs in every person, that's not a reasonable approach. But I think that's, in part, where some diagnostic delays happen is that perhaps they didn't have a high enough pretest probability to do the subsequent downstream test. [Provider (117)]	I would think knowing that I am also in a medical field that you would have at least tried to explain to me. I did not think that I should have had to be like, I'm not leaving until somebody explains what happens. I do not think that I should have had to do that. [Patient (101)]
**Prolonging self-managing of symptoms.**	**Not being informed of abnormal findings on tests.**	**Not receiving molecular testing.**
He could not go to sleep at night so he would be taking NyQuil and it was more of a consistent thing. [caregiver (102)]	When I looked at that hospital discharge summary and saw that there were lung nodules there. I had not seen that before or ever been told or followed up about that. [caregiver (102)]	I had one or two patients diagnosed with lung cancer who went to a smaller local facility for their follow-up, and I brought them back into the office to go through things. And I said, "Well, I don't see any molecular testing on your biopsy."

For peer review only - http://bmjopen.bmj.com/site/about/guidelines.xhtml

		You've lost that opportunity [Thoracic oncologist (115)]
**Seeking care only as symptoms became severe.**	**Communicated information poorly and left with uncertainty.**	**Feeling steered to receive unnecessary chemotherapy.**
One night I woke up because I felt like my left side of the chest was closing down on me, I felt that my left side was not getting enough air in. [Patient (101)]	Nobody talked to her about the mass, but she was reading her orders and she was like, "What is this in my orders?" [Caregiver (111)]	They were very pushy with the chemotherapy which made me even more skeptical. I was just saying, "Wait a minute. I would like a second opinion." I had texted the doctor and said, "they want to do chemo immediately." She said, "No, let us wait. I will get him in next week and we can do a genetic test that can see if chemo is the best for him or targeted medicine." That was the first time I had heard about targeted medicine. [Caregiver (102)]
	So who do I call? Which doctor's office do I call? If I'm having a problem, do I go to the ER? Do I go to Urgent Care? Do I go to the pulmonologist because my cousin goes to one? Or do I go to my primary care doctor? [Provider (113)]	

For peer review only - http://bmjopen.bmj.com/site/about/guidelines.xhtml

For peer review only

Factors Leading To Disparity In Lung Cancer Diagnosis

Herein, we describe the factors that lead to suboptimal experiences along the pathways to diagnosis and treatment for BAA patients. These factors work at multiple individual, interpersonal, community, and societal levels. Tables 3 includes supportive quotes.

Individual Factors

- **Not seeking timely care.** Some patients reported an aversion to seeking timely medical care for fear of finding serious illnesses or having to deal with adverse outcomes.
- **Inadequate reporting of relevant symptoms**. The patients' experiences with disease symptoms influence their decisions on which symptoms to report. They may not feel comfortable reporting certain symptoms and may understate the details.
- **Logistic constraints to accessing healthcare.** Patients living in poorer areas tend to have poorer healthcare outcomes due to affordability and income issues. At times, patients live far from the health facility and are unable to access transportation or assistance to travel for treatment.
- **Avoiding medical care due to a previous encounter involving racial discrimination**. Perceptions of the HCP based on previous experiences often lead to mistrust and hesitancy among patients to avoid possible dismissals or maltreatment.
- **Cultural identity**. People are often influenced by their community's behavior and religious beliefs regarding healthcare.
- **Lack of health literacy**. At times, patients are unable to appreciate the severity of a diagnosis or are unaware of what to do when they experience health changes.
- **Not knowing which HCP to contact**. Information on HCPs and their specialties are not readily available, making it hard for the patient to determine who to reach out to and where.

Interpersonal Factors

- **Lack of compassionate family support.** For some individuals, weaker family ties lead to socioeconomic isolation, which obscures the management of their healthcare needs.
- **Doctors' schedules not accommodating patients.** In certain areas, the doctors' offices or health facilities are only open during weekdays, when many people are at work and are therefore unable to access healthcare.
- **Societal pressure to seek non-medical approaches.** It is often observed that the patient's social networks encourage the use of alternative treatment methods, including herbal and non-regulated remedies. As a result, some patients may choose spiritual practices or herbs over medical care.
- **Patients' surroundings normalize tolerating symptoms.** Patients seldom internalize the need to endure the disease and fear the loss of dignity through their dependence on others, especially where the symptoms are common around them.
- **HCPs indulging in discriminatory practices**. It has been observed that HCPs tend to neglect, miss, or dismiss symptoms reported by BAA patients, especially pain. Furthermore, they may not execute appropriate referrals.

For peer review only - http://bmjopen.bmj.com/site/about/guidelines.xhtml

- **Patients anticipating prejudice and discrimination**. Patients are concerned about how HCPs may treat them based on their personal attributes, especially race. Others, especially individuals who used to smoke, avoid seeking medical care due to the fear of being blamed for their illness and the associated stigma of causing cancer.

Community Factors

- **Cultural practices concerning healthcare.** Certain therapies may be preferred in a community, for example, obtaining painkillers instead of identifying the cause of the ailment.
- **Availability of clinical services.** There is a dearth of hospitals, diagnostic facilities, primary care providers (PCPs), and specialists within certain communities and areas. This is especially true for smaller communities living in poor areas.
- **Disjointed clinical services**. Referrals and visits to different departments and hospitals may force patients to drop out of treatment and follow up on results. Furthermore, communicating the results to PCPs after visiting the emergency room is left to the patient and not communicated directly, which often leads to missed opportunities for follow-up on diagnosis and treatment.
- **Delays in obtaining appointments**. Although booking systems for hospitals are now online, navigating these systems is complex and coupled with organizational constraints in the availability of appropriate staff. A patient's diagnosis may be delayed due to a lack of appointment slots.
- **Organizational demands on HCPs to see more patients.** For profitable practice, some hospitals and clinics set patient and charge goals for HCPs, which leads to poor service quality. Also, forcing HCPs to attend to more patients within a stipulated time may overwhelm them, reducing their quality of care.

Societal Factors

- **Working in lower-paying jobs.** The relegation of certain communities to low-paying jobs limits their access to resources for managing health and disease impacts. This economic disempowerment leaves personal healthcare needs competing for priority with one's family's needs. Often, family commitments deny the patient the appropriate time to seek treatment.
- **Cost of receiving care**. Seeking diagnosis and treatment is costly for individuals, especially when they face job insecurity and can take limited time off from work. This issue, coupled with the inflated costs of medical care, makes healthcare services inaccessible or undesirable.
- **Limited or variable insurance coverage**. Limited options within Medicaid coverage, cost of insurance, and the inability to obtain insurance led to suboptimal access to timely diagnosis and treatment. Besides, the unwillingness of insurance companies to pay for certain services, either in full or part, may limit one's options for quality care.
- **Policies and laws determining investment in healthcare**. The existing policies and laws related to healthcare and resources available to communities affect access, education, and living conditions for the community.

For peer review only - http://bmjopen.bmj.com/site/about/guidelines.xhtml

- **Limited management using telehealth**. Only using telehealth and the absence of physical testing and comprehensive workups lead to missed diagnoses.
- **Practitioner's inexperience**. Underprivileged communities are often subjected to care by undertrained practitioners and a shortage of specialists. A lack of adequate skill and experience to manage chronic diseases may lead to lower suspicion or missing critical symptoms.
- **Complex health systems not designed for vulnerable patients**. Moving through complex online hospital systems and departments to book and access essential services requires internet access either by computer or phone, which can be challenging for many.

For peer review only

Table 3. Supportive Quotes for The Factors Leading to Disparity in Lung Cancer Diagnosis

Individual Factors	Interpersonal Factors	Community Factors	Societal Factors
**Not seeking timely care.**	**Lack of compassionate family support.**	**Cultural practices concerning health care.**	**Working in lower-paying jobs.**
Yeah. I mean, I think it's related to, "I hope that things will get better!" It can also be related to a form of denial So, in fact, they know it might be lung cancer and are not going in as a form of denial. [Provider (117)]	In the majority in a community, you're more likely to have a wider network within that community. If you have a cough, you're more likely to be talking to somebody about it and somebody said, " You should get it checked out." If you are more isolated within the community because of race or economic issues [you won't have] that network [provider (115)]	Some people take their symptoms more seriously than others. I don't know whether this is more or less among African Americans, but to the extent to which symptoms are minimized or ignored, that's more personal or cultural. [provider (117)]	A lot in African-American and Hispanic Communities do not have insurance, rather because they cannot afford healthcare insurance. Because they are making minimum wage jobs or it just not on the priority list of "I have never been sick [Advocate (108)]
**Inadequate reporting of relevant symptoms.**	**Doctors' schedules not accommodating patients.**	**Availability of clinical services.**	**Cost of receiving care**.
What's important to the patient and to the doctor are two different things. So, they may not consider their shortness of breath and issue because they've been having it over a period of time. [Provider (113)]	A lot of jobs, you know, the timing, like the doctor's offices, closes at five o'clock, and they normally take their last patient at 4.30. [advocate(108)]	I think it's also where is the setting of the low-dose CT scans. Most of them are in hospitals, or some patients just won't go to a hospital for care. [Provider (114)]	They try to wait it out because they have a job, they can't take off work without taking PTL, or just they're doing something else for their family. [nurse navigator (118)]
**Logistic constraints to accessing healthcare.**	**Societal pressure to seek non-medical approaches.**	**Disjointed clinical services**.	**Limited or variable insurance coverage**.
If I want to get somebody screened for lung cancer, they may have to travel to a hospital 30 minutes away and not have even the gas money. If I want to get somebody for a biopsy and for specialty care, they're potentially looking at traveling an hour.' [provider (115)]	Sometimes patients will say, "well you know, God heals and God will heal me" or they'll say "I want to try an herbal natural remedy. [provider(113)]	Then with a diagnosis just sending her home with that order, I think was irresponsible. Somebody should have said, "Hey, do you know you have this on your lung", and nobody did.. [caregiver(111)]	Well, this is really being driven by the insurance company. Your doctor really making some of these decisions. The insurance company made it for them. And that doesn't fit with your "medical condition" at the moment [caregiver (110)].
**Avoiding medical care due to a previous encounter involving racial discrimination**.	**The patient's surroundings normalize tolerating symptoms.**	**Delays in obtaining appointments**.	**Policies and laws determining investment in healthcare**.

For peer review only - http://bmjopen.bmj.com/site/about/guidelines.xhtml

People have shared that they felt their expression of their pain level wasn't taken as seriously, because there might be some unconscious biases on the part of the healthcare provider about what they think about Black people and their threshold for pain tolerance. '[109]	I think sometimes people don't think things are serious because it's all perception. So, if you have a family member, who let's say, everybody in your household smokes and everybody complains of shortness of breath, you probably think that it's normal to be short of breath. [113]	Just figuring that out the next step of scheduling an appointment having to get on the phone and wait to schedule an appointment. It's not as easy as getting on to your computer sometimes, depending on the Health Care system.' [113]	It relates to what resources are available in your area. If you live in a rural community and you're the only doctor in that community and the nearest specialist is 2 hours away that can have an effect on your referral process [113]
**Cultural identity**.	**Hcps indulging in discriminatory practices**.	**Organizational demands on hcps to see more patients.**	**Limited management using telehealth**.
Sometimes it is just simply cultural beliefs. They don't want to admit that they have a problem. I mean, that could be something as simple as they have a pain somewhere. You know, i'll just take Tylenol or I'm coughing I won't go to the doctor, or they'll say things like you know. [provider (113)]	So, at the doctor level and, across cultures, they see an African-American person and they may or may not be obese, but there's so much emphasis on "You need to lose weight. You need to exercise. You need to eat right." And again, might be not investigating the extent by which the drinking and the smoking. [caregiver (119)]	If you have a new patient and someone telling you have to see patients every 15 minutes and you're at the end of the day or the middle of day and you're just trying to get through it. The provider can feel rushed and glazed over the patient's symptoms' [provider (113)]	He ended up going to get some antibiotics via telemedicine from a doctor. But I think I noticed more more persistent coughing. [caregiver (102)]
**Lack of health literacy**.	**Patients anticipating prejudice and discrimination**.		**Practitioner's inexperience**.
If there's no understanding of medicine, medical illness would be deemed as something annoying, like a cough. Not understanding risk factors for disease. Based on either smoking or family history or other people with those symptoms, not understanding when to seek care. [provider (114)]	It was awful. If he had taken the opportunity to send me for an x-ray and I had great insurance. It's not like I was like, hey, let's penny-pinch. I do not want to do this, or I do not want to do that." I followed all of the instructions that he had given me, down to the tee and it was never an option for him to send me for an x-ray. [patients (106)]		I truly believe that if you recognize something you order it in a timely manner, but if you don't recognize it, I think part of it is recognition of the problem if you don't recognize it, it can delay you'. [provider (113)]
**Not knowing which HCP to contact**.			**Complex health systems not designed for vulnerable patients**.
Finding a doctor is so messed up. You get no information on a doctor. You are given a list of names and you are supposed to pick one. Well, how am I supposed to pick them based on the name? That is the first barrier. [researcher (103)]			You have been in a hospital before. It is almost impossible to navigate a hospital because parking is difficult. Getting in the maze. Knowing where to go and where you cannot go is difficult. [researcher (103)]

For peer review only

DISCUSSION

This study is one of the first to report on the suboptimal experiences of BAA patients along the lung cancer care pathway. These experiences were identified by BAA patients, caregivers, and HCPs. The study found that some patients delayed seeking care, while others had their concerns ignored or were not offered molecular testing despite qualifying for it. Factors contributing to these suboptimal experiences included difficulties with insurance coverage, provider unwillingness to conduct comprehensive testing, provider bias in recommending treatment, high healthcare costs, and a lack of healthcare facilities and qualified staff to provide necessary tests and support. The study captured firsthand the individual and caregiver experiences, which were corroborated by community advocates and some healthcare provider experiences, particularly those with the lived experience of being BAAs.

Our findings indicate that at least some of the patients in the study, all BAA, experienced delays in both the diagnosis and initiation of treatment. Some patients received treatment for other common diseases before tests revealed their lung cancer. These delays are consistent with the 2021 State of Lung Cancer (SOLC) report, which found that BAAs patients were 16% less likely to receive an early diagnosis and 7% more likely to go untreated compared to their White American counterparts.(34) HCPs' actions in offering diagnostic testing play a critical role in patient experiences. Positive relationships with HCPs can motivate patients to seek care, but we found that delays were sometimes caused by patients' concerns about discrimination from providers who may not refer them for timely diagnosis and targeted treatment. The literature shows that BAA patients often perceive communication with physicians as less supportive, less collaborative, and less informative.(35) Our findings on communication and information sharing align with this, as we observed instances of critical information being withheld, findings being communicated in ways that were difficult for patients to understand, and information on available treatment options not being shared.

The factors we identified as contributing to delays in lung cancer diagnosis may also lead to poorer outcomes throughout the cancer care process.(36) BAA patients presenting at later stages of illness may experience delays for several reasons, such as limited health literacy and self-assessment of symptoms. Previous experiences with discrimination may also impact patients' decisions about seeking care. Additionally, patients have reported feeling that their providers did not consider their symptoms to be serious, resulting in the treatment of other possible infections instead of additional testing.(37, 38) On the other hand, patients in our study reported using various approaches to overcome the barriers they faced in their healthcare journey, including utilizing their knowledge of health systems, seeking information on diagnostic and treatment options, and engaging with their insurance companies and HCPs to access comprehensive tests and treatment options. This underscores the importance of patient empowerment in advocating for their rights with insurance companies and HCPs, as well as the significance of receiving sufficient information from healthcare providers to make informed decisions about their healthcare. Patient navigators may also assist patients and families in navigating the health system and overcoming socioeconomic challenges. Addressing racism and prejudices through multilevel strategies, including retraining doctors, engaging policymakers, and educating nonclinical staff on prejudice, systemic biases, and racism, is crucial.

For peer review only - http://bmjopen.bmj.com/site/about/guidelines.xhtml

This project helped mapping the range of suboptimal experiences, which can then be included in future surveys to screen patients for these occurrences. Future research should aim to collect experiences of disparity from a more representative population and develop community-led interventions targeting individual and systemic factors perpetuating disparity. Evidence-based research methods should also focus on collecting patient perspectives relevant to experiences of racism, prejudice, and perceived injustices.

CONCLUSION

This study revealed suboptimal experiences and factors contributing to the disparities in lung cancer care among BAAs. Delays in diagnosis and initiation of treatment were reported, and patients faced various barriers, including insurance coverage, provider bias, and lack of healthcare facilities. BAA patients' empowerment and engagement with HCPs and insurance companies were important in navigating these barriers. Multilevel strategies are needed to address racism and prejudices in the healthcare system. Future research should focus on developing culturally-tailored community-centered interventions and evidence-based initiatives to collect, analyze, and disseminate patient perspectives on experiences of racism and perceived injustices. Although there were limitations to the study, the triangulation of perspectives and the use of robust frameworks strengthened the findings.

For peer review only

For peer review only - http://bmjopen.bmj.com/site/about/guidelines.xhtml

CONTRIBUTORSHIP STATEMENT

The listed authors, Nicholas Thuo (NT), Tanimola Martins (TM), Eugene Manley, Jr. (EMJ), Maisha Standifer (MS), Dawood H. Sultan (DHS), Nick Faris (NF) Angel Hill (AH), Matthew Thompson (MT), Rohan D. Jeremiah (RDJ), and Morhaf Al Achkar (MA) contributed to the conception, design, acquisition of data, or analysis and interpretation of data, drafting the article or critically revising it for important intellectual content, and final approval of the published version. NT and MA conceptualized this article and wrote the first draft along. NT, MA, TM, EMJ, NF, RDJ, MS, DHS, AH, and MT contributed to the revision and additional changes for the article to accurately reflect the research findings. MA affirms that the manuscript is an accurate representation of the issues covered, that there are no significant omissions or discrepancies. All authors have full responsibility for the work and decision to publish it.

COMPETING INTERESTS

All authors report no competing interests.

SOURCE OF FUNDING:

Funding: This research is linked to the CanTest Collaborative, which is funded by Cancer Research UK [C8640/A23385]. The project was also partially funded by a gift from LUNGevity Foundation.

DATA SHARING STATEMENT

The authors confirm that the data supporting the findings of this study are available within the article and its supplementary materials. The data will become available immediately after publication, with no end date for anyone who wishes access.

ETHICAL APPROVAL

Ethics approval was obtained from the University of Washington (UW) Human Subjects Division Reference number STUDY00005438.

For peer review only - http://bmjopen.bmj.com/site/about/guidelines.xhtml

BIBLIOGRAPHY

1. Ryan BM. Lung cancer health disparities. Carcinogenesis. 2018;39(6):741-51.
2. Soneji S, Tanner NT, Silvestri GA, Lathan CS, Black W. Racial and ethnic disparities in early-stage lung cancer survival. Chest. 2017;152(3):587-97.
3. Robbins HA, Engels EA, Pfeiffer RM, Shiels MS. Age at cancer diagnosis for blacks compared with whites in the United States. Journal of the National Cancer Institute. 2015;107(3):dju489.
4. Xu G, Cui P, Zhang C, Lin F, Xu Y, Guo X, et al. Racial disparities in bone metastasis patterns and targeted screening and treatment strategies in newly diagnosed lung cancer patients. Ethnicity & health. 2022;27(2):329-42.
5. American CancerSociety . Cancer Facts & Figures for African Americans 2022-2024. 2022.
6. Lathan CS, Neville BA, Earle CC. The effect of race on invasive staging and surgery in non–small-cell lung cancer. Journal of Clinical Oncology. 2006;24(3):413-8.
7. Hardy D, Liu CC, Xia R, Cormier JN, Chan W, White A, et al. Racial disparities and treatment trends in a large cohort of elderly black and white patients with nonsmall cell lung cancer. Cancer. 2009;115(10):2199-211.
8. Onega T, Duell EJ, Shi X, Demidenko E, Goodman DC. Race versus place of service in mortality among medicare beneficiaries with cancer. Cancer: Interdisciplinary International Journal of the American Cancer Society. 2010;116(11):2698-706.
9. Alvidrez J, Castille D, Laude-Sharp M, Rosario A, Tabor D. The national institute on minority health and health disparities research framework. American Journal of Public Health. 2019;109(S1):S16-S20.
10. Dempsey DA, Meyers MJ, Oh SS, Nguyen EA, Fuentes-Afflick E, Wu AH, et al. Determination of tobacco smoke exposure by plasma cotinine levels in infants and children attending urban public hospital clinics. Archives of pediatrics & adolescent medicine. 2012;166(9):851-6.
11. Haiman CA, Stram DO, Wilkens LR, Pike MC, Kolonel LN, Henderson BE, et al. Ethnic and racial differences in the smoking-related risk of lung cancer. New England Journal of Medicine. 2006;354(4):333-42.
12. Ho JT, Tyndale RF, Baker TB, Amos CI, Chiu A, Smock N, et al. Racial disparities in intensity of smoke exposure and nicotine intake among low-dependence smokers. Drug and Alcohol Dependence. 2021;221:108641.
13. Samet JM, Avila-Tang E, Boffetta P, Hannan LM, Olivo-Marston S, Thun MJ, et al. Lung cancer in never smokers: clinical epidemiology and environmental risk factors. Clinical Cancer Research. 2009;15(18):5626-45.
14. Jonnalagadda S, Lin JJ, Nelson JE, Powell CA, Salazar-Schicchi J, Berman AR, et al. Racial and ethnic differences in beliefs about lung cancer care. Chest. 2012;142(5):1251-8.
15. Williams CD, Salama JK, Moghanaki D, Karas TZ, Kelley MJ. Impact of race on treatment and survival among US veterans with early-stage lung cancer. Journal of Thoracic Oncology. 2016;11(10):1672-81.
16. Hendryx M, O'Donnell K, Horn K. Lung cancer mortality is elevated in coal-mining areas of Appalachia. Lung Cancer. 2008;62(1):1-7.
17. Zheng L, Enewold L, Zahm SH, Shriver CD, Zhou J, Marrogi A, et al. Lung cancer survival among black and white patients in an equal access health system. Cancer Epidemiology and Prevention Biomarkers. 2012;21(10):1841-7.

For peer review only - http://bmjopen.bmj.com/site/about/guidelines.xhtml

18. Brzezniak C, Satram-Hoang S, Goertz H-P, Reyes C, Gunuganti A, Gallagher C, et al. Survival and racial differences of non-small cell lung cancer in the United States Military. Journal of general internal medicine. 2015;30(10):1406-12.
19. Palazzo LL, Sheehan DF, Tramontano AC, Kong CY. Disparities and trends in genetic testing and erlotinib treatment among metastatic non–small cell lung cancer patients. Cancer Epidemiology and Prevention Biomarkers. 2019;28(5):926-34.
20. Li T, Kung H-J, Mack PC, Gandara DR. Genotyping and genomic profiling of non–small-cell lung cancer: implications for current and future therapies. Journal of Clinical Oncology. 2013;31(8):1039.
21. Kehl KL, Lathan CS, Johnson BE, Schrag D. Race, poverty, and initial implementation of precision medicine for lung cancer. JNCI: Journal of the National Cancer Institute. 2019;111(4):431-4.
22. Cote ML, Haddad R, Edwards DJ, Atikukke G, Gadgeel S, Soubani AO, et al. Frequency and type of epidermal growth factor receptor mutations in African Americans with non-small cell lung cancer. Journal of Thoracic Oncology. 2011;6(3):627-30.
23. Palazzo LL, Sheehan DF, Tramontano AC, Kong CY. Disparities and Trends in Genetic Testing and Erlotinib Treatment among Metastatic Non–Small Cell Lung Cancer PatientsGenetic Testing and Erlotinib Treatment. Cancer Epidemiology, Biomarkers & Prevention. 2019;28(5):926-34.
24. Lynch JA, Berse B, Rabb M, Mosquin P, Chew R, West SL, et al. Underutilization and disparities in access to EGFR testing among Medicare patients with lung cancer from 2010–2013. BMC cancer. 2018;18(1):1-13.
25. Bruno DS, Hess LM, Li X, Su EW, Zhu YE, Patel M. Racial disparities in biomarker testing and clinical trial enrollment in non-small cell lung cancer (NSCLC). Wolters Kluwer Health; 2021.
26. Harrison S, Judd J, Chin S, Ragin C. Disparities in lung cancer treatment. Current oncology reports. 2022;24(2):241-8.
27. Campbell JD, Lathan C, Sholl L, Ducar M, Vega M, Sunkavalli A, et al. Comparison of prevalence and types of mutations in lung cancers among black and white populations. JAMA oncology. 2017;3(6):801-9.
28. Evans III N, Grenda T, Alvarez NH, Okusanya OT. Narrative review of socioeconomic and racial disparities in the treatment of early stage lung cancer. Journal of Thoracic Disease. 2021;13(6):3758.
29. Morris AM, Rhoads KF, Stain SC, Birkmeyer JD. Understanding racial disparities in cancer treatment and outcomes. Journal of the American College of Surgeons. 2010;211(1):105-13.
30. Wisnivesky JP, McGinn T, Henschke C, Hebert P, Iannuzzi MC, Halm EA. Ethnic disparities in the treatment of stage I non–small cell lung cancer. American journal of respiratory and critical care medicine. 2005;171(10):1158-63.
31. Alhazmi AA, Kaufmann A. Phenomenological qualitative methods applied to the analysis of cross-cultural experience in novel educational social contexts. Frontiers in Psychology. 2022;13:785134.
32. Walter F, Webster A, Scott S, Emery J. The Andersen Model of Total Patient Delay: a systematic review of its application in cancer diagnosis. Journal of health services research & policy. 2012;17(2):110-8.

For peer review only - http://bmjopen.bmj.com/site/about/guidelines.xhtml

33. Al Achkar M, Suchsland MZ, Walter FM, Neal RD, Goulart BH, Thompson MJ. Experiences along the diagnostic pathway for patients with advanced lung cancer in the USA: a qualitative study. BMJ open. 2021;11(4):e045056.
34. American Lung Cancer Association. State of lung cancer 2021. 2021.
35. Gordon HS, Street Jr RL, Sharf BF, Kelly PA, Souchek J. Racial differences in trust and lung cancer patients' perceptions of physician communication. Journal of clinical oncology. 2006;24(6):904-9.
36. Zuniga PVS, Ost DE. Impact of delays in lung cancer treatment on survival. Chest. 2021;160(5):1934-58.
37. Lubuzo B, Ginindza T, Hlongwana K. Exploring barriers to lung cancer patient access, diagnosis, referral and treatment in Kwazulu-Natal, South Africa: The health providers' perspectives. Translational Lung Cancer Research. 2019;8(4):380.
38. Carter-Harris L, Hermann CP, Draucker CB. Pathways to a lung cancer diagnosis. Journal of the American Association of Nurse Practitioners. 2015;27(10):576-83.

For peer review only

Figure1. Suboptimal experiences throughout the diagnosis pathway.

For peer review only

For peer review only - http://bmjopen.bmj.com/site/about/guidelines.xhtml

Figure1. suboptimal experiences throughout the diagnosis pathway.

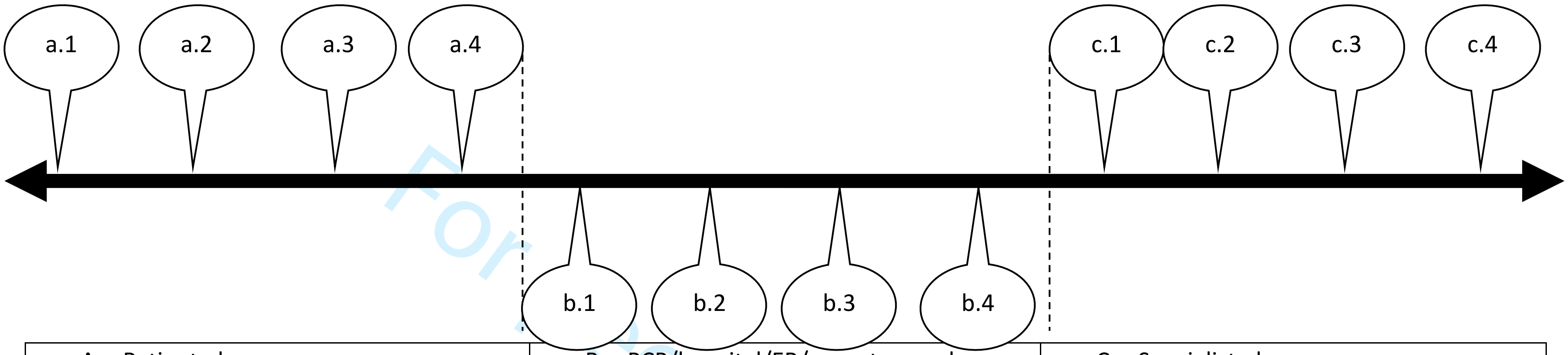

A. Patient phase	B. PCP/hospital/ER/urgent care phase	C. Specialist phase
a.1. Not being alarmed by the symptoms as they appeared	b.1. Being treated for common, more benign conditions	c.1. Difficulty finding an appointment to see the specialist
a.2. Explaining symptoms as caused by other conditions	b.2. Not offered diagnostic imaging	c.2. Poor delivery of information by the provider
a.3. Prolonging self-managing of symptoms	b.3. Not being informed of abnormal findings on tests	c.3. Not offered indicated molecular testing
a.4. Seeking care only as symptoms became severe	b.4. Poor communication by providers leaving the patient with uncertainty	c.4. Steering patients to receive unnecessary chemotherapy

For peer review only

Appendix 1. Interview guides.

For peer review only

Semi-Structured Interview Guide

The study has two aims: Aim 1: Explore pitfalls, suboptimal experiences, and discriminatory practices in cancer diagnosis pathways for African Americans with metastatic lung cancer and oncogenic alterations. Aim 2: Identify etiologies of health disparity in cancer diagnosis for African Americans with metastatic lung cancer.

Cancer diagnosis

a. Tell me about your cancer diagnosis story, starting from when you noticed your first symptoms.

b. Patient phase
   i. What symptoms did you have that led to the cancer diagnosis?
   ii. How did the symptoms change over time before diagnosis?
   iii. Did anyone else notice your symptoms?
   iv. What did you think was the cause of your symptoms?
   v. How concerned were you about your symptoms and why?
   vi. What prompted you to seek medical care the first time?
   vii. Where did you seek care for the first time? Who else did you see?
   viii. How long do you think your symptoms were present before you sought care?
   ix. In your opinion, what contributed to that time (delay) between symptoms starting and seeking medical care?

c. PCP phase
   i. Describe your first interaction with your healthcare providers when you presented with symptoms.
   ii. What took place during the visit?
   iii. How did the provider explain your symptoms?
   iv. What were the steps taken to investigate your symptoms?
   v. What was the follow-up plan?
   vi. Were you offered any treatment for your symptoms? What was it?
   vii. Were you referred anywhere else? To whom?
   viii. How long after your doctor's visit did you get your first imaging test?
   ix. How was the decision made to get an imaging test?
   x. How were the findings on the imaging tests explained to you?
   xi. Was there any other tests ordered? What are they?
   xii. How much wait time did you have between your doctor's visit and getting the tests?
   xiii. In your opinion, what contributed to that time (delay) between seeking medical care and getting imaging tests?

d. Specialty care phase
   i. How was the decision made that you should see a specialist?
   ii. Describe the interactions with the specialists (pulmonologist, surgeon, radiologist, etc.) that led to getting a biopsy to confirm the diagnosis.
   iii. How were the results explained to you?
   iv. What other tests or procedures did you have early on?
   v. Share about your first interaction with the oncologist.
   vi. How much wait time did you have between receiving the cancer diagnosis and seeing an oncologist?
   vii. How was the *diagnosis* explained to you? What did they say about the disease you have?

For peer review only

viii. How was the *prognosis* explained to you? What did they say about what to expect will happen?
ix. What did they explain about the treatment options?
x. How was the decision made to get molecular testing?
xi. How were the tests explained to you?
xii. How were the results explained to you?
xiii. What treatment did you receive first?
xiv. How was the decision made to get a specific treatment?
xv. What other treatments have you received?
xvi. How much wait time did you have between your oncologist visit and starting treatment?

e. Before cancer
   i. How was your health before cancer?
   ii. Share about your lifestyle before cancer.
   iii. Have you smoked? If so, have you been offered smoking cessation?
   iv. How much did you smoke? If significant amount, have you been offered lung cancer screening?
   v. Have you had exposure to any carcinogen?
   vi. Share about encounters with healthcare before cancer.
   vii. What health issues did you deal with before cancer?
   viii. Did you have a PCP? Tell me about them? if not, why?

f. Reflections on etiologies of potential delays
   i. You mentioned, [example of possible inadequate/delay/suboptimal], from your vantage point, why do you think this happened?
   ii. What can be done to avoid such pitfalls from happening to other people who maybe in similar positions to yours?
   iii. (rephrase questions of focus group to ask patients/caregivers to identify factors and structures contributed to less than optimal outcomes for them or other people in their situation?)

Demographics

- What is your age?
  - Under 18
  - 18-24
  - 25-44
  - 45-64
  - Over 65
- What is your gender?
  - Male
  - Female
  - Other (please specify)
  - Prefer not to say
- What is your race/ethnicity?
  - White
  - Hispanic/Latino
  - Black/African American
  - Native American/American Indian
  - Asian/Pacific Islander
  - Other

For peer review only

- What is the highest level of school you've completed?
  - Less than a high school diploma
  - High school diploma or equivalent
  - Some college
  - Bachelor's degree
  - Master's degree
  - Doctorate
  - Other (please specify)
- What is your current employment status?
  - Employed Full-time (40 hours per week)
  - Employed Part-time (Less than 40 hours per week)
  - Unemployed
  - Student
  - Retired
  - Self-employed
  - Unable to work
- What is your home zip code?
- What is your marital status?
  - Single (Never married)
  - Married
  - Divorced
  - Widowed
- What is your household income?
  - Below $10K
  - $10K – $50K
  - $50K – $100K
  - $100K - $150K
  - Over $150K
- Cancer date of diagnosis:____
- Type of cancer:____
- Stage at time of diagnosis:____
- Stage now if not IV at time of diagnosis:____

For peer review only

Health Care Providers and Community Advocates

- We define cancer health disparity as the health difference, on the basis of one or more health outcomes, that adversely affects black/african americans. Specifically, We are interested in Identifying etiologies of health disparity around cancer diagnosis for African Americans with advanced lung cancer and oncogenic alterations.
- We want you help understanding the factors that cause this disparity. We are using the NIMHD model for health disparity. According to this framework, we examining the issue across 5 domains and 4 levels. See figure.

Domains of Influence (Over the Lifecourse)	Levels of Influence*: Individual	Interpersonal	Community	Societal
Biological	Biological Vulnerability and Mechanisms	Caregiver–Child Interaction<br>Family Microbiome	Community Illness Exposure<br>Herd Immunity	Sanitation<br>Immunization<br>Pathogen Exposure
Behavioral	Health Behaviors<br>Coping Strategies	Family Functioning<br>School/Work Functioning	Community Functioning	Policies and Laws
Physical/Built Environment	Personal Environment	Household Environment<br>School/Work Environment	Community Environment<br>Community Resources	Societal Structure
Sociocultural Environment	Sociodemographics<br>Limited English<br>Cultural Identity<br>Response to Discrimination	Social Networks<br>Family/Peer Norms<br>Interpersonal Discrimination	Community Norms<br>Local Structural Discrimination	Social Norms<br>Societal Structural Discrimination
Health Care System	Insurance Coverage<br>Health Literacy<br>Treatment Preferences	Patient–Clinician Relationship<br>Medical Decision-Making	Availability of Services<br>Safety Net Services	Quality of Care<br>Health Care Policies
Health Outcomes	Individual Health	Family/ Organizational Health	Community Health	Population Health

This is the cancer diagnosis pathway.

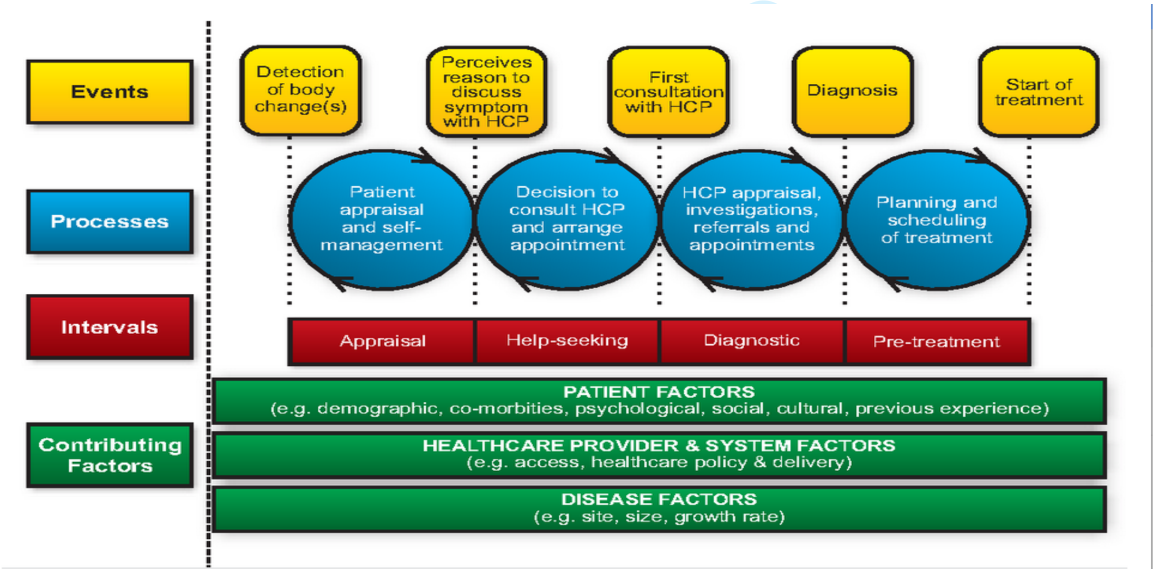

1. What are factors and structures that lead to delays in detecting first symptoms?

2. What are factors and structures that lead to inappropriate appraisal of symptoms?
3. What are factors and structures that lead to spending longer time in self-management phase as opposed to seeking help?
4. What are factors and structures that lead to not perceiving reasons to discuss symptoms with health care providers in a timely way?
5. What are factors and structures that lead to delays in consulting health care providers?
6. What are factors and structures that lead to not being able to arrange for appointments?
7. What are factors that lead to inadequate appraisal of symptoms by the health care provider on early visits?
8. What are factors and structures that lead to not ordering appropriate investigations in a timely manner?
9. What are factors and structures that lead to not arranging for referrals once suspicion of cancer become high (i.e., delays in getting to be seen by a specialist)?
10. What are factors and structures that lead to failing to obtain molecular testing
11. What are factors and structures that lead to not getting molecular testing?
12. What are factors and structures that lead to delays in getting molecular testing?
13. What are factors and structures that lead to delays in starting appropriate targeted therapy?

Caregiver's interviews are similar but rephrased to get the perspectives of the significant other on the health/experience of the patient.

Demographics

- What is your age?
  - Under 18
  - 18-24
  - 25-44
  - 45-64
  - Over 65
- What is your gender?
  - Male
  - Female
  - Other (please specify)
  - Prefer not to say
- What is your race/ethnicity?
  - White
  - Hispanic/Latino
  - Black/African American
  - Native American/American Indian
  - Asian/Pacific Islander
  - Other
- What is the highest level of school you've completed?
  - Less than a high school diploma
  - High school diploma or equivalent
  - Some college

For peer review only

  - Bachelor's degree
  - Master's degree
  - Doctorate
  - Other (please specify)
- What is your current employment status?
  - Employed Full-time (40 hours per week)
  - Employed Part-time (Less than 40 hours per week)
  - Unemployed
  - Student
  - Retired
  - Self-employed
  - Unable to work
- What is your home zip code?
- What is your marital status?
  - Single (Never married)
  - Married
  - Divorced
  - Widowed
- What is your household income?
  - Below $10K
  - $10K – $50K
  - $50K – $100K
  - $100K - $150K
  - Over $150K

For peer review only

Standards for Reporting Qualitative Research (SRQR)*

http://www.equator-network.org/reporting-guidelines/srqr/

Title and abstract

	Page/line no(s).
**Title** - Concise description of the nature and topic of the study Identifying the study as qualitative or indicating the approach (e.g., ethnography, grounded theory) or data collection methods (e.g., interview, focus group) is recommended	1
**Abstract** - Summary of key elements of the study using the abstract format of the intended publication; typically includes background, purpose, methods, results, and conclusions	3

Introduction

**Problem formulation** - Description and significance of the problem/phenomenon studied; review of relevant theory and empirical work; problem statement	5
**Purpose or research question** - Purpose of the study and specific objectives or questions	6

Methods

**Qualitative approach and research paradigm** - Qualitative approach (e.g., ethnography, grounded theory, case study, phenomenology, narrative research) and guiding theory if appropriate; identifying the research paradigm (e.g., postpositivist, constructivist/ interpretivist) is also recommended; rationale**	6
**Researcher characteristics and reflexivity** - Researchers' characteristics that may influence the research, including personal attributes, qualifications/experience, relationship with participants, assumptions, and/or presuppositions; potential or actual interaction between researchers' characteristics and the research questions, approach, methods, results, and/or transferability	7
**Context** - Setting/site and salient contextual factors; rationale**	6
**Sampling strategy** - How and why research participants, documents, or events were selected; criteria for deciding when no further sampling was necessary (e.g., sampling saturation); rationale**	6
**Ethical issues pertaining to human subjects** - Documentation of approval by an appropriate ethics review board and participant consent, or explanation for lack thereof; other confidentiality and data security issues	6
**Data collection methods** - Types of data collected; details of data collection procedures including (as appropriate) start and stop dates of data collection and analysis, iterative process, triangulation of sources/methods, and modification of procedures in response to evolving study findings; rationale**	6

For peer review only - http://bmjopen.bmj.com/site/about/guidelines.xhtml

**Data collection instruments and technologies** - Description of instruments (e.g., interview guides, questionnaires) and devices (e.g., audio recorders) used for data collection; if/how the instrument(s) changed over the course of the study	6
**Units of study** - Number and relevant characteristics of participants, documents, or events included in the study; level of participation (could be reported in results)	7
**Data processing** - Methods for processing data prior to and during analysis, including transcription, data entry, data management and security, verification of data integrity, data coding, and anonymization/de-identification of excerpts	7
**Data analysis** - Process by which inferences, themes, etc., were identified and developed, including the researchers involved in data analysis; usually references a specific paradigm or approach; rationale**	7
**Techniques to enhance trustworthiness** - Techniques to enhance trustworthiness and credibility of data analysis (e.g., member checking, audit trail, triangulation); rationale**	7

Results/findings

**Synthesis and interpretation** - Main findings (e.g., interpretations, inferences, and themes); might include development of a theory or model, or integration with prior research or theory	7-11
**Links to empirical data** - Evidence (e.g., quotes, field notes, text excerpts, photographs) to substantiate analytic findings	7 (tables)

Discussion

**Integration with prior work, implications, transferability, and contribution(s) to the field** - Short summary of main findings; explanation of how findings and conclusions connect to, support, elaborate on, or challenge conclusions of earlier scholarship; discussion of scope of application/generalizability; identification of unique contribution(s) to scholarship in a discipline or field	11-12
**Limitations** - Trustworthiness and limitations of findings	

Other

**Conflicts of interest** - Potential sources of influence or perceived influence on study conduct and conclusions; how these were managed	13
**Funding** - Sources of funding and other support; role of funders in data collection, interpretation, and reporting	13

*The authors created the SRQR by searching the literature to identify guidelines, reporting standards, and critical appraisal criteria for qualitative research; reviewing the reference lists of retrieved sources; and contacting experts to gain feedback. The SRQR aims to improve the transparency of all aspects of qualitative research by providing clear standards for reporting qualitative research.

For peer review only

**The rationale should briefly discuss the justification for choosing that theory, approach, method, or technique rather than other options available, the assumptions and limitations implicit in those choices, and how those choices influence study conclusions and transferability. As appropriate, the rationale for several items might be discussed together.

Reference:

O'Brien BC, Harris IB, Beckman TJ, Reed DA, Cook DA. **Standards for reporting qualitative research: a synthesis of recommendations.** *Academic Medicine*, Vol. 89, No. 9 / Sept 2014 DOI: 10.1097/ACM.0000000000000388

For peer review only
